# Curcumin in the Treatment of Various Diseases: Mechanism of Action, Bioavailability, Therapeutic Effects, and Current Clinical Trials

**DOI:** 10.1002/mco2.71124

**Published:** 2026-11-05

**Authors:** Lin Wang, Qifei Pan, Wenjuan Long, Xinyao Li, Jianqin Mao, Sitong Zhou, Zihui Wang, Xuelian Zou, Yuqing She, Mingqin Shi, Yang Zhou, Jiarong Du, Zhongyang Hu, Xiaoman Lv, Yishu Chen, Dongdong Qin

**Affiliations:** ^1^ Key Laboratory of Traditional Chinese Medicine for Prevention and Treatment of Neuropsychiatric Diseases Yunnan University of Chinese Medicine Kunming Yunnan China; ^2^ College of Pharmaceutical Science Dali University Dali Yunnan China

**Keywords:** bioavailability, curcumin, mechanism of action, therapeutic effects, various diseases

## Abstract

Curcumin is a naturally occurring polyphenolic compound with anti‐inflammatory, antioxidant, antiapoptotic, and anticancer properties. Numerous studies have demonstrated that curcumin can regulate multiple molecular targets, including signaling pathways such as Toll‐like receptor 4 (TLR4)/myeloid differentiation primary response 88 (MyD88)/nuclear factor kappa B (NF‐κB), Janus kinase (JAK)/signal transducer and activator of transcription (STAT), PTEN‐induced kinase 1 (PINK1)/Parkin, Parkin, phosphoinositide 3‐kinase (PI3K)/protein kinase B (Akt), mitogen‐activated protein kinase (MAPK), and nuclear factor erythroid 2‐related factor 2 (Nrf2)/antioxidant response element (ARE). Notably, curcumin exhibits therapeutic potential in a variety of diseases, including neurological disorders, cardiovascular diseases, cancer, inflammatory diseases, and autoimmune diseases. Its mechanisms of action are associated with the suppression of inflammation, regulation of mitochondrial dysfunction, inhibition of apoptosis, attenuation of oxidative stress, and enhancement of neuroplasticity. However, the extremely low oral bioavailability of curcumin limits its application in disease therapy. This review systematically summarizes recent advances in curcumin research, including its pharmacological properties, molecular mechanisms of action, and nanodelivery systems, such as nanoparticles, liposomes, nanoemulsions, polymeric micelles, and exosomes, which have been developed to enhance curcumin bioavailability. Furthermore, this review provides an in‐depth overview of the therapeutic benefits of curcumin in neurological disorders, cardiovascular diseases, cancer, inflammatory diseases, and autoimmune diseases. It focuses on evidence from in vitro studies, in vivo experiments, and potential clinical applications, while also discusses the therapeutic potential, current challenges, and future perspectives of curcumin in disease management.

## Graphical abstract

Curcumin, a naturally occurring polyphenol, exhibits significant anti‐inflammatory and antioxidant capabilities. This review elucidates the specific molecular mechanisms by which curcumin exerts therapeutic effects on various diseases, including neurological disorders, cardiovascular diseases, cancers, inflammatory disorders, and autoimmune diseases, and outlines multiple strategies to improve its bioavailability. By compiling the therapeutic advantages of curcumin across these diverse diseases, we highlight its broad pharmacological potential, substantiated by in vitro, in vivo, and clinical studies, with the goal of offering evidence for curcumin's potential as a novel therapy for these diseases.

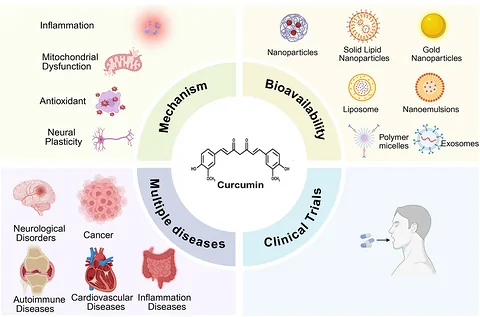

## 1 Introduction

Turmeric, a key member of the ginger family, originated in India and is widely used in culinary applications, traditional medicine, and dye production [1]. Historical records indicate that the use of turmeric in Indian Vedic culture dates to approximately 2500 BC. In Ayurvedic medicine, turmeric is valued for its diverse health benefits, including wound healing and the treatment of inflammatory conditions, skin diseases, and digestive disorders [2, 3]. Curcumin, a natural polyphenolic compound extracted from the turmeric rhizome, is composed of curcumin, demethoxycurcumin, and bisdemethoxycurcumin (BDMC) [4]. Owing to its diverse pharmacological properties—including anti‐inflammatory [5], antioxidant, and neuroprotective effects—curcumin has garnered significant attention in medical research [6, 7]. However, despite this therapeutic potential, factors such as low water solubility, poor stability, and rapid metabolic degradation result in low oral bioavailability, which hinders its development and clinical application [8]. Consequently, current research efforts are directed toward the development of novel drug delivery systems, including lipid nanoparticle formulations, lipid carriers, nanoemulsions (NEs), polymeric micelles, and exosomes, among others [9, 10, 11], to enhance the pharmacokinetic profiles and efficacy of curcumin.

Curcumin, the primary bioactive component of turmeric, modulates multiple signaling pathways—such as NF‐κB (nuclear factor kappaB), mitogen‐activated protein kinase (MAPK), Wnt/β‐catenin, and the phosphatidylinositol 3‐kinase/protein kinase B (PI3K/AKT) cascade—which play essential roles in inflammatory responses, apoptosis, and oxidative stress [12, 13, 14, 15, 16]. These signaling pathways significantly contribute to the pathogenesis of neurological disorders [17], cancer [18], cardiovascular diseases [19], inflammatory conditions [20], and autoimmune diseases [21]. In addition, sustained microglial activation mediated by pattern recognition receptors, including Toll‐like receptors (TLRs) and the NLRP3 inflammasome, can induce neuronal injury through the release of inflammatory mediators and oxidative stress [22]. At present, research on curcumin has primarily focused on elucidating its therapeutic targets across a variety of diseases [23]. Although the interactions of curcumin with various molecular targets have been extensively reported, a comprehensive synthesis of the specific mechanisms underlying its therapeutic effects in diverse diseases is still lacking. A deeper understanding of these mechanisms is crucial for informing the development of curcumin‐based therapeutic strategies and their eventual clinical application.

In summary, curcumin has attracted significant research interest owing to its long history in traditional medicine and its substantial therapeutic potential in biomedical research. Its structural versatility, broad pharmacological activities, and low bioavailability constitute key avenues for future investigation. With ongoing elucidation of its molecular mechanisms and continued advancements in drug delivery systems, curcumin is expected to become a core therapeutic agent for the treatment of various diseases, including neurological disorders, cancer, cardiovascular diseases, inflammatory conditions, and autoimmune diseases. This review provides a comprehensive synthesis of the specific mechanisms underlying the modulatory effects of curcumin on these conditions. Furthermore, strategies for enhancing curcumin bioavailability—focusing on novel nanoparticle formulations—are evaluated, while curcumin treatment modalities and clinical efficacy are also assessed. Ultimately, this review seeks to establish a solid theoretical foundation for curcumin research as well as to facilitate the development of novel treatments for diseases, as illustrated in graphic abstract.

## 2 Pharmacological Properties of Curcumin

Curcumin is a hydrophobic polyphenolic compound abundantly present in the rhizome of turmeric, comprising between 5 and 6% of its content. Turmeric is primarily distributed across Southeast Asia, particularly in India, where its rhizome is utilized as a food additive and spice due to its yellow pigment and aromatic volatile oils [24]. Curcumin exhibits a wide range of pharmacological properties, including anti‐inflammatory, antioxidant, antirheumatic, antitumor, antifibrotic, antidiabetic, antiobesity, cardioprotective, antithrombotic, lipid‐modulating, and antiviral effects [24, 25, 26]. Research has shown that curcumin exerts ameliorative effects on neurological diseases via the targeting of multiple signaling pathways [27]. Additionally, clinical studies have demonstrated that curcumin is nontoxic to humans at doses of up to 8 g per day over 3 months, indicating that it is safe even at high dosages [28].

Despite the significant safety and therapeutic potential of curcumin in various diseases, its application is limited by low bioavailability [29]. Pharmacokinetic studies have reported that the oral bioavailability of curcumin is only approximately 1% [30], primarily attributable to its low aqueous solubility, poor absorption, and rapid metabolic transformation [24, 31]. Notably, curcumin is prone to degradation under alkaline conditions, decomposing within 30 min at pH > 7. Its degradation occurs more slowly under acidic conditions, with approximately 20% being decomposed within 1 h [32]. Furthermore, most orally administered curcumin is rapidly metabolized in the liver and plasma, with a large proportion being excreted in its unchanged form [33, 34].

Over recent years, novel approaches have been explored to enhance the bioavailability and biocompatibility of curcumin in vivo, including the use of liposomes, micelles, and advanced nanoparticle formulations. These strategies have aimed at overcoming challenges associated with poor solubility, inadequate absorption, and rapid metabolism. For instance, Yang et al. [35] reported that nanoparticles derived from curcumin–bovine serum albumin (BSA) exhibit sustained release characteristics, thereby prolonging drug half‐life and reducing dosing frequency, while simultaneously increasing blood–brain barrier permeating ability. These nanoparticles exert anti‐inflammatory and neuroprotective effects by inhibiting macrophage M1 polarization and blocking inflammatory signaling pathways [35].

In another study, the curcumin NE exerted cardioprotective effects through inhibition of angiotensin‐converting enzyme (ACE) activity and pronounced antioxidant activity [36]. To prevent curcumin degradation in the gastric environment, a colloidal delivery system loaded with curcumin was developed, which reduced its release in the stomach, enabling it to reach the intestine intact and exert therapeutic effects at inflammatory sites [37]. Lin et al. [38] prepared curcumin‐loaded cationic liposomes and observed that these liposomal formulations inhibited tumor angiogenesis. Yeh et al. [39] investigated the therapeutic potential of curcumin in osteoarthritis by encapsulating curcumin within liposomes with stable particle characteristics. The results demonstrated that curcumin effectively reduced macrophage differentiation, removed mineral deposits in chondrocytes, and decreased the production of proinflammatory cytokines.

Studies indicate that curcumin possesses the potential to prevent and treat neurological disorders, cancer, cardiovascular diseases, inflammatory conditions, and autoimmune diseases through its anti‐inflammatory, antioxidant, and antiapoptotic properties, as well as by enhancing neuroplasticity [7, 40, 41, 42, 43]. Curcumin exerts neuroprotective effects by downregulating the expression of proinflammatory cytokines (e.g., IL‐1β, IL‐6, TNF‐α) and the transcription factor NF‐κB [43]. One study reported the protective effects of curcumin in apolipoprotein E‐deficient mice, showing that curcumin attenuated the progression of aortic aneurysms in atherosclerotic mice, reduced macrophage infiltration and the production of proinflammatory mediators, and inhibited the activation of the extracellular signal‐regulated kinase (ERK) signaling pathway, while simultaneously increasing superoxide dismutase (SOD) levels [44]. Recognized as a potent natural antioxidant, curcumin significantly increases glutathione (GSH) levels and reduces the production of reactive oxygen species (ROS), which effectively alleviates neuronal cytotoxicity [45]. Neural cell apoptosis is a primary mechanism underlying degeneration following brain injury. Notably, studies have indicated that curcumin exerts strong antiapoptotic effects in the brain [46] by increasing the expression of BCL‐2 and reducing that of BAX and Caspase‐3, thereby providing neuroprotection against ischemic damage. Importantly, this mechanism has been replicated in the N2a cell model of ischemic stroke [47]. Furthermore, curcumin treatment was found to induce a concentration‐dependent downregulation of antiapoptotic proteins and upregulation of proapoptotic proteins. These findings suggest that curcumin represents a novel therapeutic approach for inhibiting synovial fibroblast proliferation in rheumatoid arthritis (RA) [48]. Studies have also reported that enhanced neuroplasticity can promote recovery from neurological disorders [17]. Increased levels of brain‐derived neurotrophic factor (BDNF) contribute to the enhancement of neuroplasticity, modulating the expression of plasticity‐related genes, repairing neuronal damage, and alleviating depressive symptoms [42]. In the following section, we summarize the potential mechanisms by which curcumin exerts its therapeutic effects on neurological disorders, cancer, cardiovascular diseases, inflammatory conditions, and autoimmune diseases.

## 3 The Mechanism Underlying the Effects of Curcumin

Curcumin is a polyphenolic compound extracted from the rhizome of curcuma longa (turmeric) and exhibits a wide range of pharmacological activities, including anti‐inflammatory, antioxidant, antiapoptotic, and neuroprotective effects. In recent years, curcumin has been extensively investigated, and numerous studies have demonstrated its therapeutic potential in various pathological conditions, including neurological disorders, cardiovascular diseases, cancer, inflammatory diseases, and autoimmune diseases. Current evidence indicates that curcumin exerts its therapeutic effects through multiple molecular mechanisms, including the suppression of inflammatory responses, amelioration of mitochondrial dysfunction, modulation of signaling pathways, such as PI3K/Akt, MAPK, and Nrf2/ARE, and promotion of neuroplasticity. Therefore, as a naturally derived bioactive compound with multitarget properties, curcumin holds considerable potential as a promising candidate for the development of therapeutic agents against various diseases. Based on recent advances in this field, this section provides an overview of the major molecular mechanisms underlying the biological effects of curcumin.

### 3.1 The Mechanism by Which Curcumin Exerts its Anti‐Inflammatory Effects

Inflammation is a complex physiological and pathological process, typically serving as an adaptive response triggered by harmful stimuli such as infection and tissue injury to maintain organismal homeostasis. Its severity is largely dependent on the nature of the stimulus or injury, disease stage, and duration, and it may lead to immune cell recruitment, tissue damage, and even cell death. Acute inflammation promotes tissue repair and pathogen clearance, whereas chronic inflammation drives disease progression through a feed‐forward loop involving damage‐associated molecular patterns and sustained immune activation [49]. These inflammatory states are characterized by the excessive production of proinflammatory cytokines (such as IL‐1β, IL‐6, and TNF‐α), chemokines, and ROS [50, 51, 52]. Notably, the NLRP3 inflammasome is a pivotal molecular complex that amplifies cascade responses by promoting the maturation of IL‐1β and IL‐18 and inducing pyroptosis, serving as a primary and synergistic driver in a variety of chronic inflammation‐associated diseases. Dysregulated activation of NLRP3 contributes to the progression of conditions such as RA, neurological disorders, and atherosclerosis [53].

Due to its multitarget actions and favorable safety profile, curcumin is considered a promising agent for the modulation of inflammation [54]. Curcumin has been shown to exert immunomodulatory effects on macrophages, inhibiting M1 polarization while promoting the anti‐inflammatory M2 phenotype. These effects occur primarily through the inhibition of the NF‐κB/NLRP3 and JAK/STAT signaling pathways, alongside the downregulation of proinflammatory cytokines (such as IL‐6 and TNF‐α) and the upregulation of anti‐inflammatory mediators, such as TGF‐β and IL‐10 [54]. Collectively, these findings underscore the therapeutic potential of curcumin in alleviating inflammation through immunomodulatory mechanisms (Figure 1).

**FIGURE 1:**
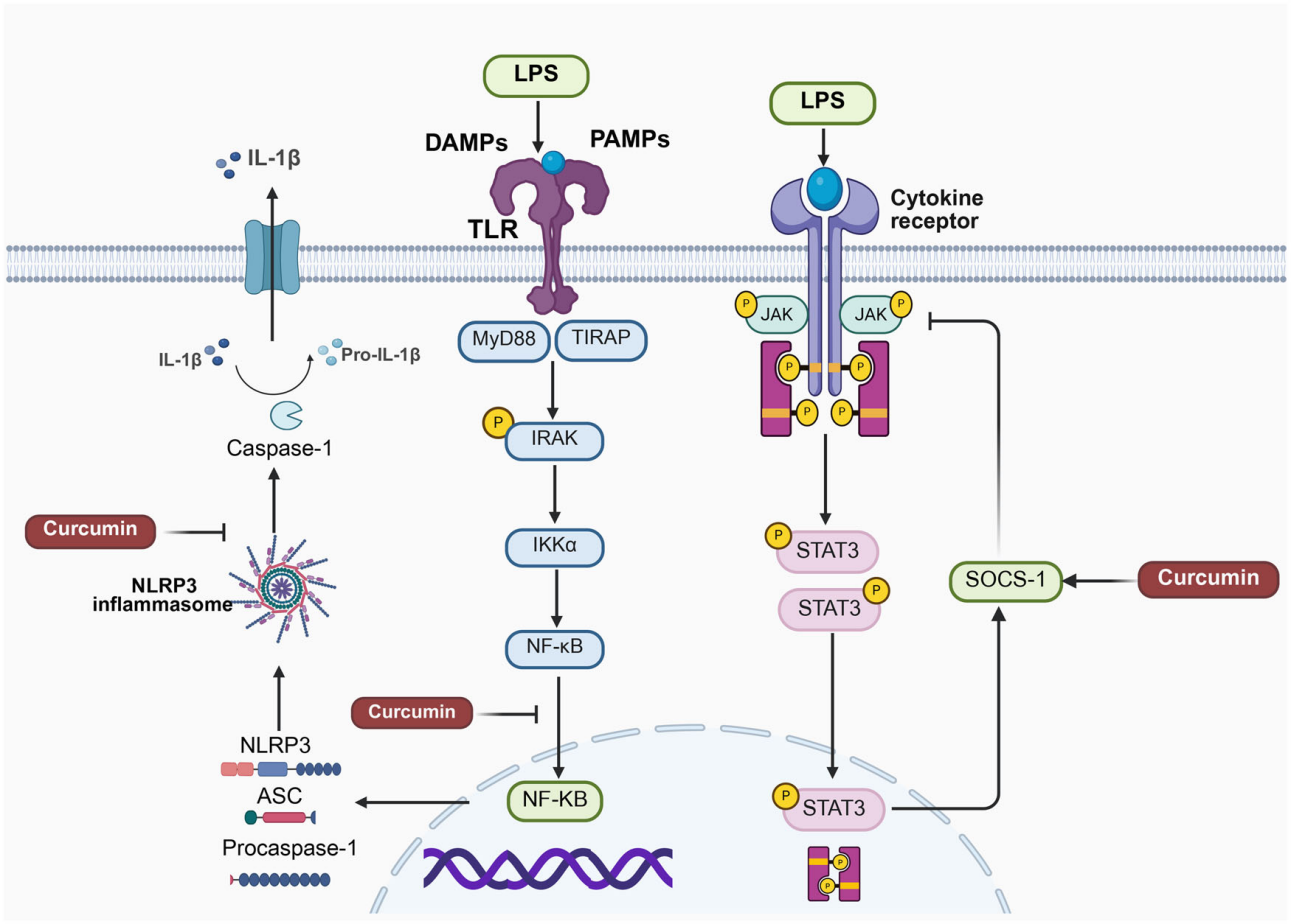
Anti‐inflammatory mechanisms of curcumin. Lipopolysaccharide (LPS) is recognized by Toll‐like receptor 4 (TLR4), leading to the recruitment of myeloid differentiation primary response 88 (MyD88) and TIRAP and the subsequent activation of the MyD88‐dependent signaling pathway. MyD88 activation promotes the phosphorylation of IRAK, which, in turn, activates IKKα and triggers NF‐κB signaling. Curcumin inhibits the TLR4/MyD88/NF‐κB signaling pathway, thereby modulating inflammatory responses, suppressing immune cell activation and the production of proinflammatory factors under various diseases conditions, and consequently exerting anti‐inflammatory and tissue‐protective effects. Under LPS stimulation, the JAK2/STAT3 signaling pathway is upregulated, resulting in the increased secretion of proinflammatory mediators. Curcumin modulating inflammatory signaling by upregulating the protein expression of suppressor of cytokine signaling 1 (SOCS‐1), which inhibits the phosphorylation of JAK2/STAT3.

#### 3.1.1 TLR4/MyD88/NF‐κB Signaling Pathway

The TLR family consists of transmembrane receptors that detect the presence of pathogens by recognizing PAMPs or DAMPs, subsequently activating innate immune responses to defend the host [55]. Upon activation, TLRs induce the release of cytokines and initiate intracellular stress responses [56]. In mammals, TLRs also induce the expression of effector molecules, including inducible nitric oxide synthase (iNOS) and antimicrobial peptides, that kill microbial pathogens [57]. NF‐κB acts as an effector transcription factor for TLR4. Notably, NF‐κB is a key modulator of inflammatory responses in neurons and microglia, an effect mediated via the upregulation of proinflammatory cytokine expression. Upon binding to lipopolysaccharide (LPS), TLR4 recruits adaptor proteins, such as myeloid differentiation factor 88 (MyD88) and TIR domain‐containing adaptor protein (TIRAP), thereby activating the MyD88‐dependent pathway [58]. This activation leads to the phosphorylation of interleukin‐1 receptor‐associated kinase (IRAK), which subsequently activates the IκB kinase (IKK) complex. NF‐κB, maintained in an inactive state through its interaction with IκB, is released following the IKK‐mediated phosphorylation of IκB. This process, which can be stimulated by macrophages and inflammatory cytokines, enables NF‐κB to drive the production of proinflammatory factors [59].

Neurological injury is often accompanied by microglia‐mediated inflammatory responses, with the TLR4/MyD88/NF‐κB signaling pathway playing a vital role in this process. The activation of TLRs can promote the production of cytokines, adhesion molecules, and chemokines [60]. The absence of TLR4, which is predominantly expressed in microglia, can induce a shift in the polarization of these cells from the M1 to the M2 phenotype, facilitating their migration and subsequently alleviating the progression of neuroinflammation while simultaneously improving neurological function [61]. Zhang et al. treated microglia with curcumin to evaluate its anti‐inflammatory effects and found that it inhibits the TLR4/NF‐κB signaling pathway. This inhibition reduced the levels of proinflammatory factors (e.g., iNOS, IL‐6, IL‐1β), while upregulating those of anti‐inflammatory mediators (e.g., arginase 1 [Arg‐1], IL‐4, IL‐10, CD206), thus modulating microglial polarization and exerting neuroprotective effects [14]. Moreover, administering curcumin via intraperitoneal injection inhibits the neuroinflammatory cascade, thereby alleviating neuronal damage and behavioral deficits, an effect mediated through the attenuation of TLR4/MyD88/NF‐κB signaling pathway activation [62]. In models of cerebral ischemia, the activation of the NLRP3 inflammasome triggers the cleavage of Caspase‐1, which, in turn, cleaves gasdermin D (GSDMD), pro‐IL‐1β, and pro‐IL‐18, generating the respective active forms (GSDMD‐N, IL‐1β, and IL‐18). In a mouse model of middle cerebral artery occlusion (MCAO), curcumin treatment alleviated postischemic white matter damage and improved sensorimotor dysfunction through the inhibition of the NLRP3/NF‐κB signaling pathway [63]. While neuronal integrity and neurological function are compromised in neurological disorders, neuroinflammation further exacerbates their progression. Studies involving TLR4 knockout mice indicated that curcumin administration following neural injury promotes a microglial phenotypic shift toward the M2 state, thus reducing neuroinflammation, likely via the inhibition of the TLR4/MyD88/NF‐κB signaling pathway. These findings highlight the potential of curcumin as a therapeutic agent for alleviating neuroinflammatory responses in neurological disorders [52].

Coronary microembolization (CME)‐induced local myocardial inflammation is one of the major contributors to myocardial injury and subsequent progressive cardiac dysfunction. Following CME, TLR4 activates the NF‐κB signaling pathway via MyD88, promoting the release of inflammatory mediators such as TNF‐α and IL‐1β, ultimately resulting in cardiomyocyte injury and apoptosis [19]. In this context, previous studies have reported that inhibitors of the TLR4/MyD88/NF‐κB signaling pathway exert protective effects against CME‐induced myocardial injury in vivo [64]. Liu et al. [65] found in a rat CME model that CME significantly elevated serum cardiac Troponin I levels, increased inflammatory cytokine expression, and enhanced the myocardial cell apoptosis index, leading to marked deterioration of cardiac function. Curcumin attenuated myocardial inflammatory responses and exerted cardioprotective effects by inhibiting the TLR4/MyD88/NF‐κB signaling pathway. It has also been reported that TLR4/MyD88 expression is markedly upregulated in cancer and contributes to tumor metastasis; and curcumin treatment significantly suppresses cancer cell proliferation and migration, potentially through inhibition of the TLR4/MyD88 signaling pathway [66]. In a mouse model of ulcerative colitis (UC), curcumin alleviated disease symptoms, reduced inflammation, and restored mucosal barrier function by upregulating ZO‐1 and Claudin expression. Its anti‐inflammatory effects were mediated through the TLR4/MyD88/NF‐κB pathway, accompanied by suppression of proinflammatory cytokines and macrophage activation [67]. Another study investigated the protective effects of curcumin in sodium monoiodoacetate‐induced osteoarthritis, and the results demonstrated that curcumin reduced the expression of proinflammatory factors and alleviated joint swelling and cartilage damage by inhibiting the TLR4/MyD88/NF‐κB signaling pathway [68].

Collectively, these data establish the TLR4/MyD88/NF‐κB signaling pathway as a key regulator of inflammation, playing an essential role in ameliorating neurological disorders, cancer, cardiovascular diseases, inflammatory conditions, and autoimmune diseases.

#### 3.1.2 JAK/STAT Signaling Pathway

The JAK/STAT pathway is a major mediator of cytokine signaling, playing a critical role in initiating innate immunity, coordinating adaptive immune processes, and ultimately mediating inflammatory and immune responses [69]. This pathway is frequently activated in neurological conditions, cancer, cardiovascular diseases, inflammatory conditions, and autoimmune diseases [70]. Diverse cytokines associated with autoimmune inflammation, such as IL‐6, IL‐10, and IFN‐γ, transduce intracellular signals through the JAK/STAT pathway [71]. Notably, the levels of IFN‐γ and IL‐6, the most potent activators of this pathway, are elevated in inflammatory diseases [70].

In a rat model of PD induced by alpha‐synuclein (α‐Syn), inhibition of the JAK/STAT pathway suppresses microglial activation and the production of proinflammatory cytokines and chemokines, thereby reducing neuroinflammation [72]. Research has shown that enhancing the expression of triggering receptor expressed on myeloid cells 2 effectively suppresses the phosphorylation of JAK2 and STAT3. This promotes the polarization of microglia from the M1 to the M2 phenotype and decreases the synthesis and release of inflammatory cytokines [73], thereby alleviating cognitive deficits in mice [74]. Additionally, under LPS stimulation, the downregulation of the JAK/STAT signaling pathway significantly reduces the secretion of proinflammatory mediators from microglia [75]. Suppressor of cytokine signaling 1 (SOCS‐1) acts as a negative regulator of STAT3, inhibiting IL‐6 signaling and interfering with the migration of immune cells in the CNS [76]. Curcumin exerts anti‐inflammatory effects via the modulation of the JAK/STAT signaling pathway. Under LPS stimulation, JAK2/STAT3 signaling in microglia rapidly increases, whereas curcumin treatment inhibits this pathway by upregulating SOCS‐1 protein levels, which enhances IL‐10 and IL‐4 expression in microglia. These changes indicate that curcumin mediates anti‐inflammatory activity through SOCS‐1, which inhibits JAK2/STAT3 phosphorylation, thereby alleviating neural damage [77]. A different study demonstrated that curcumin inhibits the JAK2/STAT3 signaling pathway, reducing inflammation following cerebral ischemia and providing neuroprotective effects. Ham et al. [78] found that cancer‐associated fibroblasts (CAFs) can secrete cytokines such as IL‐6 and CCL2 to activate the JAK2/STAT3 signaling pathway in gastric cancer cells, thereby enhancing resistance to 5‐fluorouracil (FU) chemotherapy. Following curcumin treatment, phosphorylation of JAK2/STAT3 and the downstream expression of the antiapoptotic protein BCL‐2 were significantly inhibited, which in turn increased the chemosensitivity of gastric cancer cells and attenuated CAF‐induced chemoresistance. Studies have shown that in an ovarian cancer rat model, curcumin treatment significantly reduced IL‐6 levels and inhibited JAK2 expression as well as STAT3 phosphorylation, thereby suppressing tumor cell proliferation and promoting apoptosis [79]. Zhao et al. [80] investigated the mechanism of curcumin in a mouse model of colitis by examining JAK2/STAT3/SOCS signaling‐mediated dendritic cell (DC) activation. The results demonstrated that curcumin restored immune homeostasis and effectively ameliorated colitis by modulating the JAK2/STAT3/SOCS pathway and inhibiting DC activation. In addition, curcumin significantly inhibited the proliferation, migration, and invasion of human osteosarcoma cells in a dose‐dependent manner. It induced G0/G1 cell cycle arrest and apoptosis through suppression of the JAK2/STAT3 pathway, thereby inhibiting tumor growth [81]. It has also been reported that oncostatin M induces the expression of MMP‐1 and MMP‐3 in chondrocytes via activation of the JAK/STAT signaling pathway. Curcumin treatment markedly inhibited STAT phosphorylation and blocked JAK/STAT signal transduction, leading to the downregulation of MMP gene expression [82].

These findings suggest that curcumin exerts anti‐inflammatory and chondroprotective effects by inhibiting the JAK/STAT signaling pathway [83].

### 3.2 Curcumin Modulates Mitochondrial Dysfunction

Mitochondria, double‐membrane organelles present in all eukaryotic cells, use electrochemical energy from reducing equivalents for the production of adenosine triphosphate (ATP) [84]. These organelles are responsible for providing energy in the form of ATP for diverse cellular processes. Disruption or damage to mitochondria can lead to alterations in cellular bioenergetics, redox balance, and mitochondrial dynamics. Furthermore, mitochondria participate in ROS metabolism, calcium signaling, and apoptosis [85], playing a critical role in regulating synaptic plasticity, cancer cell proliferation, cardiomyocyte homeostasis, and inflammatory responses. Consequently, mitochondrial dysfunction is recognized as a major contributor to the pathogenesis of multiple diseases [86]. Specifically, changes in mitochondrial function activate several neuropathological processes, including the dysregulation of Ca^2+^ homeostasis, oxidative stress, mitophagy, mitochondrial DNA mutations, and apoptosis [87]. Despite the varying initial causes of these signals, the resulting tissue injury and disease initiation and progression generally involve similar underlying mechanisms. This mechanistic overlap emphasizes the importance of these mechanisms as common targets for intervention strategies [88]. A comprehensive understanding of the processes underlying mitochondrial dysfunction allows the development of interventions capable of slowing the progression of neurological disorders, cancer, cardiovascular diseases, inflammatory conditions, and autoimmune diseases [89]. Curcumin acts on multiple targets, resulting in the mitigation of mitochondrial dysfunction and enhance mitochondrial function. It has been shown that curcumin reduces mitochondrial dysfunction and fission while enhancing fusion‐related processes, biogenesis, and the expression of synaptic proteins. Curcumin also protects mitochondrial structure and helps maintain mitochondrial function [90]. Jiang et al. demonstrated that curcumin significantly lowers intracellular ROS levels, thereby alleviating oxidative damage [91]. Below, we review the mechanisms underlying the dysregulation of Ca^2+^ homeostasis, mitophagy, and apoptosis, while assessing recent research advances regarding the impact of curcumin on these pathways (Figure 2).

**FIGURE 2:**
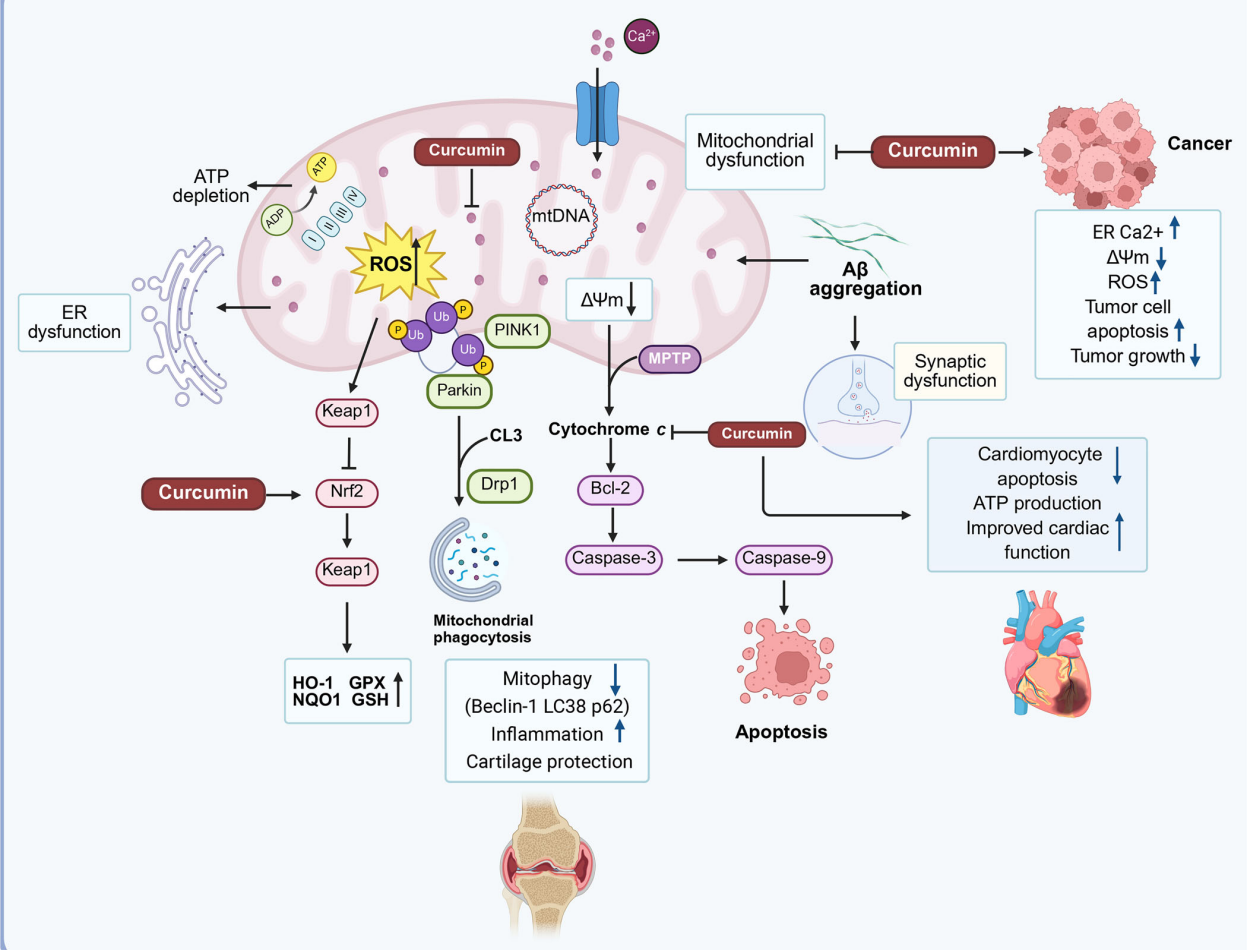
Mechanisms by which curcumin modulates mitochondrial dysfunction. The accumulation of Aβ is a critical initiating factor in Alzheimer's disease (AD), leading to Ca^2+^ overload in mitochondria and the disruption of mitochondrial homeostasis, endoplasmic reticulum dysfunction, the inhibition of the electron transport chain complexes, synaptic dysfunction, the depletion of mitochondrial DNA, and the loss of mitochondrial membrane potential (∆Ψm). This cascade results in the release of cytochrome *c* and subsequent apoptosis. These events ultimately result in cytochrome *c* release and apoptosis. A similar mechanism also occurs in cancer, where it induces tumor cell apoptosis and inhibits tumor growth. Curcumin has been shown to attenuate apoptosis, alleviate mitochondrial dysfunction, and protect against neuronal injury, while also exerting cardioprotective effects. Increased oxidative stress contributes to mitochondrial dysfunction, further elevating reactive oxygen species (ROS) levels within mitochondria. Curcumin exerts its protective effects by modifying Keap1, facilitating the binding of nuclear factor erythroid 2‐related factor 2 (Nrf2) to antioxidant response elements (ARE), thereby inhibiting Nrf2 activation and upregulating the expression of heme oxygenase‐1 (HO‐1), glutathione peroxidase (GPx), NAD(P)H: quinone oxidoreductase 1 (NQO1), and glutathione (GSH), which mitigates oxidative damage. Additionally, Parkin, in conjunction with PINK1, is involved in the clearance of damaged mitochondria through mitophagy, leading to mitochondrial dysfunction. Curcumin promotes Parkin/PINK1‐mediated mitophagy. In autoimmune diseases, curcumin promotes PINK1/Parkin‐mediated mitophagy, upregulates Beclin‐1, p62, and LC3B expression, enhances the removal of damaged mitochondria, inhibits Caspase‐3 activation, restores mitochondrial membrane potential, and maintains cell survival, thereby protecting chondrocytes and alleviating inflammatory injury.

#### 3.2.1 Dysregulation of Ca^2+^ Homeostasis

Ca^2+^ plays a critical role in numerous neuronal activities, such as the activation of signal transduction, the regulation of transcription, and the initiation of synaptic transmission. Ca^2+^ homeostasis at the plasma membrane is primarily attributed to the selective permeability properties of the plasma membrane, ATP‐dependent dynamic functions, and the sodium‐calcium exchanger system that actively pumps Ca^2+^ ions out of the cell [92, 93]. Additionally, the endoplasmic reticulum and mitochondria function as intracellular Ca^2+^ reservoirs, buffering cytosolic Ca^2+^ concentrations and maintaining them at low levels. However, when the integrity of the cell membrane or the function of ATP‐dependent Ca^2+^‐ATPase pumps/uniport systems is compromised, intracellular Ca^2+^ levels may rise. Persistent elevation of intracellular Ca^2+^ can disrupt endoplasmic reticulum function and induce neurotoxicity, manifested as mitotic arrest, the cessation of ATP synthesis, and excessive activation of calcium‐dependent hydrolases [94, 95]. Such mechanisms ultimately impair neuronal function and lead to neuronal cell death. This underscores the importance of maintaining intracellular Ca^2+^ homeostasis for neuronal survival. Neurons heavily rely on mitochondria for energy production and Ca^2+^ buffering, making them particularly susceptible to mitochondrial dysfunction. The accumulation of β‐amyloid (Aβ) constitutes a primary etiological event in Alzheimer's disease (AD), inducing mitochondrial Ca^2+^ overload and disrupting mitochondrial homeostasis [96]. One study indicated that the pathological activation of Ca^2+^‐dependent proteases and protein phosphatases may lead to neuronal damage in MS [97]. Notably, curcumin has been shown to modulate intracellular calcium release in SH‐SY5Y neuronal cells, which was associated with improved neuronal survival rates [98]. In addition, studies have shown that mitochondrial dysfunction is a critical event in the development of cardiotoxicity. Curcumin can effectively preserve mitochondrial membrane integrity and restore mitochondrial function, thereby alleviating cardiomyocyte injury and exerting cardioprotective effects [99]. Zheng et al. [100] found that curcumin promoted calcium release from the endoplasmic reticulum into the cytoplasm while inhibited calcium efflux, leading to a decrease in mitochondrial membrane potential (MMP), depletion of ATP, release of cytochrome *c*, and activation of Caspase‐3. Ultimately, these events promote tumor cell apoptosis and suppress tumor growth.

#### 3.2.2 PINK1/Parkin Signaling Pathway

PTEN‐induced kinase 1 (PINK1) and the E3 ubiquitin–protein ligase Parkin are critical factors in the regulation of mitophagy and mitochondrial biogenesis [101]. Under physiological conditions, PINK1 is imported into mitochondria and is subsequently degraded through proteolysis. Following mitochondrial depolarization and the loss of membrane potential, PINK1 rapidly accumulates on the outer mitochondrial membrane (OMM), where it recruits Parkin, leading to the initiation of mitophagy [102, 103]. Research has shown that PINK1/Parkin interact with mitochondrial genes, a process associated with the regulation of mitochondrial biogenesis and the maintenance of homeostasis [104, 105]. Following mitochondrial damage, PINK1 undergoes rapid autophosphorylation and recruits Parkin; the subsequent interaction with P62 and LC3 facilitates the tethering of damaged mitochondria to autophagosomes, ensuring the lysosomal degradation of impaired organelles by proteolytic enzymes. In patients with AD, mitochondrial function is impaired, while the levels of PINK1 are significantly reduced, underscoring the fundamental role of this protein in AD pathogenesis [106]. Moreover, in Aβ‐induced mouse models of AD, *PINK1* gene deletion promotes mitochondrial fission and fusion, which exacerbates mitochondrial dysfunction. In vitro studies have shown that PINK1‐knockdown PC12 cells exhibit increased vulnerability to Aβ‐induced cytotoxicity, suggesting that PINK1/Parkin pathway activity is essential for the preservation of neuronal viability [107]. Studies have shown that curcumin facilitates PINK1/Parkin‐mediated mitophagy, thereby preserving mitochondrial function [108]. In zebrafish exposed to 6‐hydroxydopamine (6‐OHDA) to induce neurotoxicity, curcumin treatment reduced the expression of dynamin‐related protein 1 (DRP1) while increasing that of Mitofusin 2, thus improving mitochondrial dynamics. Additionally, curcumin upregulated the PINK1/Parkin pathway, thereby contributing to the maintenance of mitochondrial homeostasis, effectively exerting neuroprotective effects [109]. Recent studies have reported that curcumin provides protection for osteoarthritis by activating PINK1/Parkin‐mediated mitophagy, thereby upregulating mitophagy‐related markers including Beclin‐1, p62, and LC3B. This process facilitates the clearance of damaged mitochondria, preserves mitochondrial integrity, and exerts protective effects on cartilage tissue [110]. Cao et al. [111] demonstrated that curcumin alleviates hydrogen peroxide‐induced oxidative stress, intestinal epithelial barrier injury, and mitochondrial damage in a mitophagy‐dependent manner via the PINK1/Parkin signaling pathway. These findings elucidate a potential molecular mechanism by which curcumin mitigates oxidative stress‐induced mitochondrial dysfunction.

#### 3.2.3 Apoptosis

Mitochondria participate in apoptosis through various pathways. In vertebrates, the loss of selective permeability in the OMM—precipitated by proapoptotic BCL‐2 family activity and concurrent organelle fission—results in mitochondrial outer membrane permeabilization (MOMP) [112]. This allows the efflux of intermembrane space proteins, such as cytochrome *c*, into the cytosol, thereby promoting caspase activation. Recent studies have found that mitochondrial fission and apoptosis are closely linked. During the apoptotic process, mitochondria undergo extensive fragmentation driven by increased recruitment of DRP1, a process crucial for its positive regulatory role in BAX/BAK‐mediated MOMP [113, 114]. Although the proapoptotic function of DRP1 is distinct from its mitochondrial structure regulatory activity, the resulting mitochondrial fragmentation likely also influences the permeability of the OMM [115]. Conversely, mitochondrial fusion is associated with the protection of cells from apoptotic death, whereas the activation of apoptosis results in the inhibition of fusion activity [116]. The regulatory network comprising BCL‐2 and proteins associated with mitochondrial dynamics may contribute to the pathogenesis of neurological disorders, cancer, cardiovascular diseases, inflammatory conditions, and autoimmune diseases. Specifically, the core components of programmed cell death include the caspase cascade, the assembly of apoptotic protease activating factor‐1 (Apaf‐1) complexes, and the heterodimerization of BCL‐2 family proteins. Additionally, mitochondria function as a central node in apoptosis through the release of apoptosis‐inducing factors, such as cytochrome *c* and apoptosis‐inducing factor [117, 118]. In this cascade, the translocation of cytochrome *c* to the cytosol induces the formation of the Apaf‐1 complex, resulting in the activation of Caspase‐9 and, subsequently, other caspases, ultimately leading to apoptosis.

In vitro cultures of rat cortical neuronal cells treated with tert‐butyl hydroperoxide (t‐BHP) exhibit significant neuronal damage, characterized by the loss of MMP (ΔΨm), the efflux of cytochrome *c* from mitochondria into the cytosol, and the subsequent activation of Caspase‐3 and PARP cleavage, ultimately inducing apoptosis. However, curcumin intervention significantly reversed these pathological conditions, indicating that curcumin attenuates t‐BHP‐induced cortical neuronal apoptosis and mitigates mitochondrial dysfunction, resulting in neuroprotection [119]. Furthermore, Zhang et al. investigated the neuroprotective effects of curcumin in glutamate‐treated rat PC12 cells and found that curcumin reduced cytochrome *c* release, the BAX/BCL‐2 ratio, and Caspase‐3 activity, thereby alleviating cytotoxicity. These findings support that curcumin exerts an inhibitory effect on the apoptotic cascade [120]. A subsequent in vitro study found that Aβ impairs mitochondrial dynamics, reduces mitochondrial biogenesis, and diminishes both synaptic activity and mitochondrial function. Notably, pretreatment with curcumin promoted mitochondrial fusion while suppressing fission‐related processes, resulting in improved mitochondrial function and cell survival rates. These findings suggest that curcumin may prevent mitochondrial dysfunction and serve as a promising drug for the treatment of neurological disorders [121]. Mitochondria play a crucial role in maintaining myocardial tissue homeostasis. Mitochondrial dysfunction ultimately leads to cardiomyocyte death and further contributes to cardiovascular impairment. Sabet et al. [99] investigated the effects of bevacizumab on mitochondria and cardiomyocytes in isolated rat hearts and evaluated curcumin as a mitochondria‐ and cardio‐protective agent. The results demonstrated that curcumin significantly alleviated bevacizumab‐induced mitochondrial toxicity and inhibited apoptosis, suggesting that curcumin may help mitigate mitochondrial damage and cardiotoxicity. Zhang et al. [122] aimed to investigate the protective effects of curcumin in a rat model of cardiac arrest induced by ventricular fibrillation. Their findings showed that curcumin improved myocardial dysfunction caused by ischemia–reperfusion (I/R) injury and restored cardiopulmonary function. In addition, mitochondrial structure and energy metabolism were improved, with enhanced ATP production and suppression of mitochondrial apoptotic pathways, including downregulation of cytochrome *c*, Caspase‐3, and uncoupling protein 2. An in vitro study revealed that curcumin exerted a significant synergistic anticancer effect against malignant melanoma cells. Curcumin induced ROS production and mitochondrial dysfunction, ultimately leading to apoptosis and programmed necrosis. This process was accompanied by upregulation of proapoptotic proteins including Bax, p53, and Caspase‐3, as well as an increased Bax/Bcl‐2 ratio [123]. In addition, in an LPS‐induced chondrocyte model of osteoarthritis, curcumin treatment markedly restored MMP, upregulated Beclin‐1 and LC3‐II expression, inhibited Caspase‐3 activation, and modulated Bcl‐2 expression. These effects promoted the clearance of damaged mitochondria and suppressed apoptosis, thereby maintaining mitochondrial homeostasis and chondrocyte survival and exerting protective effects against osteoarthritis [124].

### 3.3 Curcumin Modulates the PI3K/AKT Signaling Pathway

The PI3K/AKT signaling pathway is a crucial intracellular signaling cascade involved in the transduction of signals from cytokines and the extracellular matrix (ECM) [125]. This pathway is essential for a wide range of cellular functions, including cell proliferation, adhesion, migration, invasion, metabolism, and survival [126, 127]. PI3K is present in numerous cell types and undergoes activation by receptor tyrosine kinases (RTKs), G protein‐coupled receptors (GPCRs), and Ras family proteins on the cell membrane [128]. Activated PI3K catalyzes the conversion of phosphatidylinositol 4,5‐bisphosphate (PI (4,5) P2​) into the second messenger phosphatidylinositol 3,4,5‐trisphosphate (PIP3), which subsequently binds to downstream targets [129, 130]. The generation of PIP3 is critical for the recruitment of AKT and its subsequent activation by RTKs.

AKT consists of three isoforms, each containing an N‐terminal Pleckstrin homology (PH) domain, a central kinase domain, and a C‐terminal regulatory domain. The isolated PH domains of all AKT subtypes bind PIP3 with similar affinity. This binding sequesters AKT at the membrane, enabling its phosphorylation at Threonine 308 (Thr308) by 3‐phosphoinositide‐dependent protein kinase 1 (PDK1), also recruited by PIP3, and at Serine 473 (Ser473) by mammalian target of rapamycin complex 2 (mTORC2), events that significantly increase AKT activity [131, 132, 133]. Phosphorylated AKT (p‐AKT) subsequently directly or indirectly activates key downstream effector molecules, such as mTOR, GSK‐3β, and actin‐related proteins, thereby impacting cell growth and survival. Concurrently, mTOR interacts directly with p70 ribosomal S6 kinase 1/2 (P70S6K1/2), eukaryotic translation initiation factor 4E‐binding proteins (4E‐BPs), and other proteins involved in translational regulation, initiating protein synthesis, transcription, cell growth, and proliferation [134].

The PI3K/AKT signaling pathway plays a significant role in the pathogenesis of neurological disorders, cancer, cardiovascular diseases, inflammatory conditions, and autoimmune diseases [135]. This pathway functions as a key regulator of neuronal survival and is capable of modulating both neurogenesis and synaptic plasticity [136]. Activation of PI3K/AKT pathway signaling promotes neuronal survival under conditions of hypoxia and in in vitro models of neuronal death [137]. Numerous studies have demonstrated that inhibition of the PI3K/AKT pathway exacerbates ischemic neuronal death, while its activation significantly improves outcomes in acute brain injury [138]. Furthermore, AKT is considered an important therapeutic target, promoting neuronal survival in systemic neurological diseases, especially those involving ischemia, where excitotoxicity serves as a major cause of neuronal death [139]. AKT suppresses excitotoxicity and proapoptotic signaling through the phosphorylation of substrates such as BAD, forkhead transcription factors, and proline‐rich AKT substrates, thereby exerting neuroprotective effects [140]. GSK‐3β, a direct substrate of AKT, participates in synaptogenesis and microglial activation; however, its hyperactivation results in excitotoxic neuronal injury [141]. Such overactivity facilitates synaptic abnormalities and promotes tau hyperphosphorylation, leading to the formation of neurofibrillary tangles, a pathological hallmark of AD [142]. Notably, p‐AKT phosphorylates GSK‐3β, inhibiting its activity and exerting neuroprotective effects.

Curcumin has been widely reported to exert beneficial effects in the treatment of neurological disorders. In the 1‐methyl‐4‐phenyl‐1,2,3,6‐tetrahydropyridine (MPTP)‐induced PD mouse model, curcumin protects dopaminergic neurons from damage through the activation of the PI3K/AKT pathway, resulting in neuroprotective effects [143]. Reports indicate that curcumin alleviates brain edema and reduces neuronal death following injury, improves neurological function, and increases both autophagy and AKT phosphorylation levels. Meanwhile, the PI3K inhibitor LY294002 was found to reverse the neuroprotective effects of curcumin after brain injury, suggesting that curcumin mediates its effects through the PI3K/AKT signaling pathway [144]. Similarly, in a rat model of traumatic brain injury, curcumin administration activated the PI3K/AKT pathway, which supported the recovery of neurological function [145]. Moreover, in APP/PS1 double transgenic mice, curcumin reduces Aβ generation, enhances Aβ clearance, and induces autophagy. In these mice, curcumin triggers autophagy via the inhibition of the PI3K/AKT/mTOR signaling pathway, effectively improving memory and cognitive functions [146]. The PI3K/Akt pathway has been shown to be involved in the development of cardiovascular diseases, where Akt regulates cardiovascular functions related to cardiac growth and survival, contractile performance, and coronary angiogenesis [147]. Chen et al. [148] established a mouse model of myocardial I/R injury and demonstrated that curcumin treatment improved cardiac function by increasing ejection fraction and fractional shortening, while reduced left ventricular end‐systolic diameter and left ventricular end‐systolic volume, thereby protecting the heart from I/R‐induced heart failure. This protective effect was primarily mediated through activation of the PI3K/Akt pathway and suppression of I/R‐induced autophagy and apoptosis in cardiomyocytes. Wei et al. [149] reported that curcumin attenuated high glucose–induced cardiomyocyte apoptosis by inhibiting NADPH oxidase–mediated oxidative stress. This cardioprotective effect was likely mediated by PI3K/Akt‐related signaling pathways. Curcumin acted on multiple aspects of cancer progression, including initiation, metastasis, angiogenesis, and tumor growth. The PI3K/Akt pathway was a key therapeutic target in cancer due to its role in tumor initiation, proliferation, and cell survival [150]. In one study, curcumin downregulated Akt and mTOR expression to inhibit the proliferation of non‐small cell lung cancer cells, thereby inducing two distinct forms of cell death, namely, apoptosis and autophagy [151]. In human umbilical vein endothelial cells (HUVECs), curcumin was found to inhibit the PI3K/Akt/mTOR signaling pathway, induce apoptosis, and suppress migration and angiogenesis in hepatocyte growth factor‐treated cells. Furthermore, in experimental mouse models, curcumin inhibited hepatocyte growth factor‐stimulated tumor growth, increased E‐cadherin expression, and decreased CD34 and vascular endothelial growth factor (VEGF) expression. Collectively, these findings indicated that curcumin suppresses epithelial–mesenchymal transition and angiogenesis by blocking the PI3K/Akt/mTOR pathway [152]. Additionally, curcumin alleviated joint inflammation in arthritic rats, inhibited synovial angiogenesis and abnormal fibroblast proliferation, and exerted its effects by suppressing the expression of proinflammatory cytokines TNF‐α and IL‐6, as well as inhibiting activation of the PI3K/Akt signaling pathway [153]. Hui et al. [154] investigated the ability of curcumin to alleviate systemic lupus erythematosus (SLE) in two different mouse models. Their results showed that curcumin reduced neutrophil migration and modulated the PI3K/Akt signaling pathway to suppress inflammatory cytokine secretion, thereby improving SLE. This study provides a novel therapeutic target and strategy for the clinical application of curcumin.

Collectively, these findings suggest that curcumin alleviates various diseases through the modulation of the PI3K/AKT signaling pathway (Figure 3).

**FIGURE 3:**
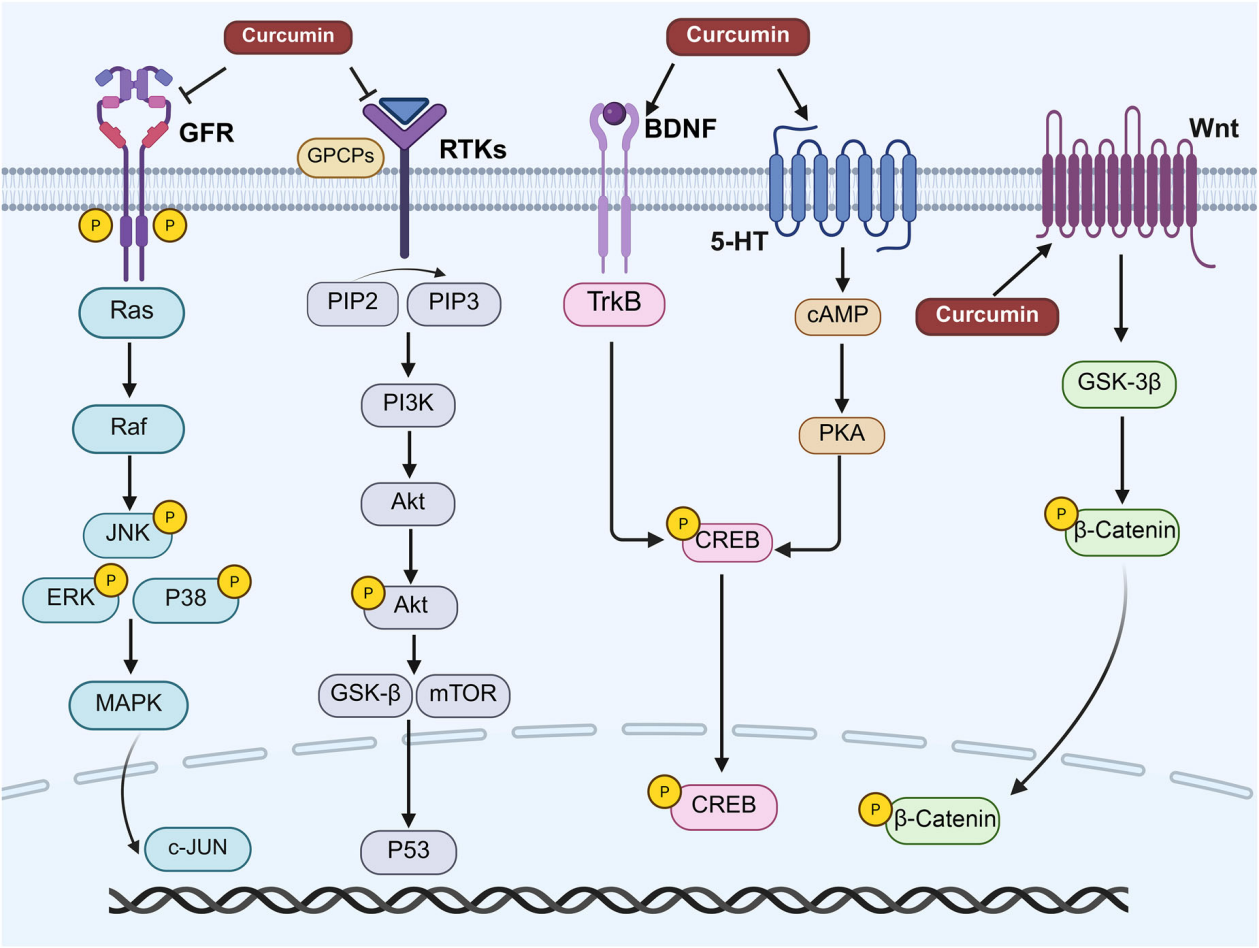
Mechanisms by which curcumin modulates the PI3K/AKT and MAPK pathways. Phosphoinositide 3‐kinase (PI3K) can be activated by protein tyrosine kinases and G‐protein‐coupled receptors (GPCRs). Once activated, PI3K converts phosphatidylinositol 4,5‐bisphosphate (PIP2) to phosphatidylinositol 3,4,5‐trisphosphate (PIP3), leading to the recruitment of AKT from the cytoplasm to the membrane, where it undergoes phosphorylation. Phosphorylated AKT activates mammalian target of rapamycin (mTOR) and glycogen synthase kinase‐3 beta (GSK‐3β). Curcumin alleviates cellular injury and inflammatory responses by inhibiting the PI3K/AKT signaling pathway and simultaneously modulating the MAPK signaling pathway. The binding of brain‐derived neurotrophic factor (BDNF) to its receptor, TrkB, results in CREB phosphorylation. Curcumin enhances CREB phosphorylation, thereby promoting BDNF‐mediated transcriptional activity. Furthermore, curcumin activates the cAMP/protein kinase A (PKA)/CREB signaling pathway through the enhancement of 5‐HT1A receptor activity, which increases neurotransmitter release. Curcumin also influences the Wnt/β‐catenin signaling pathway, promoting GSK‐3β‐mediated phosphorylation of β‐catenin, thereby contributing to neuronal repair.

### 3.4 Curcumin Modulates the MAPK Signaling Pathway

MAPKs are critical signaling molecules within eukaryotic signal transduction networks. This pathway responses to multiple diverse stimuli, linking extracellular signals with fundamental cellular signals, thereby coordinating cellular responses and intercellular communications [155]. Consequently, the regulation of MAPK activity has profound biological implications across numerous cellular processes [156], including neural differentiation, mitosis, and apoptosis. Besides transcription factors such as c‐Jun, c‐Fos, Elk‐1, and c‐Myc, MAPK substrates include membrane‐associated proteins and cytoplasmic proteins, such as kinases, cytoskeletal components, and phospholipase A2 [157]. The diversity of these target proteins indicates that MAPKs play a central role in coordinating both short‐ and long‐term cellular changes triggered by extracellular stimuli. Based on the specificity of the activating extracellular stimuli and amino acid sequence homology, MAPKs are primarily classified into three main categories: ERKs, c‐Jun N‐terminal kinases/stress‐activated protein kinases (JNK/SAPKs), and p38 MAPK [158]. Furthermore, hormones, growth factors, and cytokines accelerate MAPK phosphorylation and activation in resting cells.

Research indicates that the dysregulation of the MAPK signaling pathway is associated with the pathogenesis of neurological disorders, impacting key functions such as synaptic plasticity, learning, and memory. Elevated MAPK can lead to augmented tau hyperphosphorylation, Aβ accumulation, and memory impairments. Notably, the activity of MAPK phosphatase 1 (MKP‐1), which acts as an antagonistic regulator of MAPK via the dephosphorylation of its tyrosine and threonine residues, is downregulated in AD. The overexpression of MKP‐1 in transgenic AD mouse models has been shown to reduce Aβ production, prevent the development of neuroinflammatory plaques, diminish synaptic defects, and improve cognitive function. Zhu et al. demonstrated that inhibiting the p38 MAPK signaling pathway significantly enhances learning, memory, and motor abilities in rats following ischemic brain injury, thereby supporting neuronal recovery [159]. These findings suggest that modulating MAPK represents an effective strategy against neurological disorders. Curcumin has been shown to modulate the MAPK signaling pathway, establishing a molecular link between its neuroprotective properties and cellular activities related to neurological disorders. For example, curcumin has been found to mitigate brain injuries induced by chronic intermittent hypoxia, including brain edema and neuronal death, through the regulation of the p38 MAPK pathway [160]. The MAPK/ERK pathway, activated by glial cells during the progression of neurological disorders, participates in the transmission of cellular signals and plasticity changes, such as those related to learning and memory; these kinases serve as pivotal regulatory factors for cell proliferation and survival [161]. A separate study confirmed that curcumin reduces the release of proinflammatory cytokines, inhibits the MAPK/ERK signaling pathway, and suppresses microglial proliferation. These findings highlight the capacity of curcumin to modulate the MAPK/ERK pathway, which yields marked neuroprotective effects [162]. To enhance these effects, Huang et al. encapsulated curcumin in nanoparticles and demonstrated that curcumin reduces neuronal apoptosis through the modulation of the p38 MAPK pathway [163]. Similarly, studies have indicated that curcumin effectively alleviates neuronal damage, blood–brain barrier disruption, and brain edema by inhibiting the p38 MAPK signaling pathway, thereby improving neurological function [164]. Jeong et al. [165] established a rat model of myocardial I/R injury via ligation of the left anterior descending coronary artery. Curcumin was administered prior to ligation, and the results demonstrated that curcumin significantly reduced myocardial infarct size and effectively protected against myocardial injury by inhibiting the MAPK pathway, including p38 and JNK signaling. In another study, the effects of curcumin on human cardiac fibroblasts, fibroblast proliferation, cell cycle distribution, and collagen deposition were evaluated. The results showed that curcumin inhibited TGF‐β1‐induced proliferation, differentiation, and collagen synthesis in cardiac fibroblasts, potentially through suppression of the p38 MAPK signaling pathway [166]. Li et al. [167] investigated the effects of curcumin on thrombosis and platelet aggregation, and it was found that curcumin inhibited platelet aggregation and thrombus formation by suppressing the MAPK signaling pathway, reducing thromboxane A2 production and platelet granule release. These findings suggested potential applications in the prevention and treatment of atherosclerosis and thrombosis‐related cardiovascular diseases. Some studies have shown that curcumin promotes ROS production and upregulates p38 MAPK phosphorylation, thereby inducing apoptosis in chemotherapy‐resistant lung cancer cells and exerting antitumor effects [168]. In a study on head and neck cancer cells, curcumin activated AMP‐activated protein kinase α and p38 MAPK and upregulated peroxisome proliferator‐activated receptor gamma coactivator 1‐alpha (PGC‐1α), thereby suppressing cancer cell proliferation [169]. In a mouse model of colitis induced by dinitrobenzene sulfonic acid, curcumin inhibited activation of the p38 MAPK and NF‐κB signaling pathways and reduced IL‐1β levels, thereby alleviating intestinal inflammation [170]. In an asthma mouse model, curcumin modulated the activity of secretory phospholipase A2, reduced COX‐2 and prostaglandin D2 levels, and ultimately attenuated airway inflammation through regulation of p38 MAPK, ERK, and JNK signaling pathways [171]. Xu et al. demonstrated that curcumin significantly inhibited ERK and p38 phosphorylation as well as c‐Fos expression, thereby suppressing the MAPK/AP‐1 signaling pathway [172]. This led to reduced expression of matrix metalloproteinase‐9 (MMP‐9) and proinflammatory cytokines, resulting in alleviation of synovial inflammation, inhibition of cartilage and bone destruction, and delayed progression of RA.

In summary, these data suggest that curcumin may ameliorate various diseases through its modulatory effects on the MAPK pathway (Figure 3).

### 3.5 Curcumin Modulates the Nrf2/ARE Signaling Pathway

Oxidative stress, characterized by the excessive accumulation of ROS and reactive nitrogen species (RNS) within cells, plays a central role in the etiology of neurological disorders, cancer, cardiovascular diseases, inflammatory conditions, and autoimmune diseases. Oxidative stress in the brain can compromise the integrity of the hippocampus and amygdala; notably, the dentate gyrus of the hippocampus regulates structural plasticity, regeneration/remodeling capacity, and neurogenesis [173]. Consequently, oxidative damage in the brain can weaken remodeling capabilities, alter structural plasticity, and affect cell proliferation and neurogenesis, collectively disrupting or destroying synaptic neurotransmission and neural circuits. Specifically, these complex oxidative stress‐induced changes in the brain can lead to the impairment of cognitive and motor functions and significantly hinder neuronal tissue regeneration, culminating in irreversible tissue damage. Thus, the regulation of oxidative stress represents an effective therapeutic strategy. Although a low level of ROS is continuously generated in cells, excessive ROS production induced by pathological stimuli or failure of antioxidant defense systems is a major cause of various vascular dysfunctions. Increasing evidence indicates that oxidative stress is a key driver of endothelial dysfunction. Moreover, oxidative stress may trigger vascular inflammation and injury by activating transcription factors, upregulating adhesion molecules, stimulating chemokine production, and promoting inflammatory cell recruitment [162, 174]. It has been reported that ROS facilitates signal transduction and promotes cancer cell proliferation, differentiation, migration, and invasion [175]. However, at high concentrations, ROS can instead impair cancer cell survival due to damage to DNA, lipids, and proteins. Previous studies have demonstrated that elevated ROS levels can induce apoptosis in multiple myeloma and colorectal cancer cells. Therefore, increasing intracellular ROS levels in cancer cells beyond a tolerable threshold to induce cell death has emerged as a promising anticancer therapeutic strategy [176, 177]. In recent years, increasing attention has been paid to the role of oxidative stress in inflammatory and autoimmune diseases. For example, in RA, oxidative stress not only promotes activation of inflammatory pathways but also contributes to joint tissue degeneration, thereby accelerating disease progression and exacerbating clinical symptoms [178, 179]. Activated immune cells, including neutrophils and macrophages, release large amounts of ROS in response to proinflammatory cytokines. In addition, enhanced ROS activity can activate MMPs and promote osteoclast differentiation, both of which accelerate bone resorption and joint tissue damage [180, 181]. Curcumin is recognized for its potent antioxidant properties, which function through dual mechanisms. On the one hand, its phenolic structure endows it with the ability to directly scavenge ROS and RNS, such as superoxide anions and hydroxyl radicals [182, 183]. On the other hand, curcumin enhances cellular tolerance to oxidative damage by activating intracellular signaling pathways, reducing lipid peroxidation, increasing GSH levels, and promoting protein synthesis [184]. This capacity of curcumin to alleviate oxidative stress emphasizes its therapeutic potential against cancer, cardiovascular diseases, inflammatory conditions, and autoimmune diseases (Figure 2).

The Nrf2/ARE signaling pathway functions as a primary regulator of cellular defense against oxidative damage. Under conditions of oxidative stress, Nrf2 translocates to the nucleus and binds to the antioxidant response element (ARE). Acting as a transcription factor, Nrf2 regulates the expression of genes encoding antioxidants and detoxifying enzymes [185]. Studies suggest that curcumin facilitates Nrf2 binding to ARE by modifying the reactive thiol groups of cysteine in Kelch‐like ECH‐associated protein 1 (Keap1). Typically, the ubiquitination of Nrf2 by Keap1 and its subsequent proteasomal degradation prevent the activation of this pathway. Interaction with curcumin results in the modification of cysteine residues in Keap1, inducing conformational changes that render the inhibitory effect of Keap1 on Nrf2 ineffective [186]. Subsequently, the expression of genes encoding heme oxygenase‐1 (HO‐1), GSH peroxidase (GPx), and NAD(P)H quinone oxidoreductase 1 (NQO1) is upregulated, resulting in increased concentrations of antioxidant enzymes and GSH and the consequent mitigation of oxidative damage [187]. One study showed that curcumin reduces brain damage induced by MCAO in rats by upregulating Nrf2 and its downstream target gene *HO‐1*, providing new targets and a rationale for the treatment of cerebral ischemia [188]. Rajakrishnan et al. reported that curcumin significantly reverses lipid peroxidation and brain lipid modifications while increasing GSH levels, thus demonstrating neuroprotective effects in brain injury [189]. Additionally, curcumin was shown to activate the Nrf2/ARE pathway and upregulate the expression of antioxidant enzymes such as HO‐1 and GPx, which enhanced cellular antioxidant defenses [190]. A recent study reported that curcumin mitigates oxidative stress induced by traumatic brain injury, reducing the production of the lipid peroxidation marker malondialdehyde (MDA) and enhancing the activity of antioxidant enzymes such as SOD and GPx. Specifically, curcumin promotes the translocation of Nrf2 from the cytoplasm to the nucleus, consequently upregulating the expression of genes encoding HO‐1 and NQO‐1 and activating the Nrf2/ARE pathway. In one study, curcumin exerted therapeutic effects against prostate cancer by epigenetically activating Nrf2 transcription and subsequently triggering downstream antioxidant signaling pathways [191]. Zhang et al. [192] demonstrated that curcumin promoted nuclear translocation of Nrf2 via the AKT pathway and enhanced the expression of downstream antioxidant factors, including HO‐1 and glutamate–cysteine ligase catalytic subunit. These effects reduced ROS accumulation in diabetic cardiomyocytes, alleviated mitochondrial damage, and contributed to the treatment of diabetic cardiomyopathy. Another study showed that curcumin activated Nrf2 while concurrently inhibited the downstream cGAS–STING–NF‐κB signaling cascade, thereby effectively suppressing inflammatory activation, migration, and invasion of RA synovial fibroblasts and promoting apoptosis [193]. These findings contribute to the elucidation of how curcumin activates the Nrf2/ARE signaling pathway and identify the mechanism by which curcumin exerts antioxidant effects.

### 3.6 Curcumin Enhances Neural Plasticity

Neural plasticity is a key characteristic of the mammalian brain, defined as the inherent dynamic ability of the CNS to mature, respond to experiences, undergo structural and functional changes, and adapt following injury [194]. Notably, neural plasticity facilitates the development of neural circuits and the formation of learning and memory. Numerous studies have reported that the enhancement of neural plasticity promotes recovery from neurological disorders [195]. For instance, long‐term potentiation of synaptic transmission in the hippocampus results in the improvement of memory and cognitive function in AD patients [196]. Furthermore, increased levels of BDNF contribute to the enhancement of neural plasticity through the regulation of plasticity‐related genes, the mitigation of neuronal damage, and the reduction of depressive symptoms [197]. Research has demonstrated that curcumin treatment increases dendritic spine density in the hippocampus, a change that potentiates neural signal transmission and synaptic plasticity [198]. Overall, neural plasticity is involved in the pathogenesis of neurological disorders, and curcumin enhances neural plasticity by modulating key signaling pathways, providing a foundation for targeted therapies for these conditions (Figure 3).

#### 3.6.1 BDNF/TrkB/CREB Signaling Pathway

BDNF represents an extensively studied neurotrophic factor in the nervous system and functions as a major regulator of neural plasticity. This factor maintains neuronal survival and regeneration through the regulation of dendritic spine morphology and adult neurogenesis in the hippocampus [199]. BDNF activity involves binding to and activating the tropomyosin receptor kinase B (TrkB), an interaction that governs cellular processes essential for the development and maintenance of brain function [200]. The neuroprotective effects of BDNF are primarily mediated through the regulation of the BDNF/TrkB signaling pathway [201]. BDNF modulates neural plasticity by regulating multiple intracellular signaling pathways necessary for neuronal health and function [202]. Furthermore, the activity of the cyclic adenosine monophosphate (cAMP) response element‐binding protein (CREB) in neurons has been linked to numerous intracellular processes, including proliferation, differentiation, neurogenesis, and neuronal plasticity [203]. BDNF signals through two classes of membrane receptors: Trk and p75 neurotrophin receptor (p75NTR). The high‐affinity binding of mature BDNF to TrkB activates downstream signaling pathways. In a rat model of chronic unpredictable mild stress (CUMS), curcumin treatment reversed the decline in BDNF expression and ERK phosphorylation levels in the hippocampus, which resulted in the improvement of depressive‐like behavior and cognitive deficits [204]. A separate study in mice demonstrated that curcumin activates the CREB/BDNF signaling pathway, mitigating cadmium‐induced neurotoxicity and promoting hippocampal neurogenesis, thus ameliorating cognitive impairments. These findings indicate that curcumin promotes neuroregeneration through the modulation of the CREB/BDNF signaling pathway [205]. In rats exposed to acrylamide, the levels of both phosphorylated CREB (p‐CREB) and BDNF were significantly reduced. The administration of curcumin markedly reversed these effects in the hippocampus, effectively alleviating cognitive deficits [206]. Similarly, Shi et al. demonstrated that curcumin protects against acrylaldehyde‐induced neurotoxicity in HT22 mouse hippocampal neurons. Notably, this effect was exerted through the activation of the BDNF/TrkB signaling pathway, thereby establishing a rationale for the use of curcumin in the treatment of neurological disorders [207]. Additionally, curcumin has shown protective effects on rat hippocampal neurons through the upregulation of the BDNF/TrkB signaling pathway [208]. Meanwhile, curcumin administration significantly increased BDNF and p‐CREB protein levels in the dentate gyrus of mice. These changes resulted in the reduction of memory impairment caused by d‐galactose and were associated with the modulation of cell proliferation and neuroblast differentiation [209].

#### 3.6.2 CAMP/PKA Signaling Pathway

cAMP is a small hydrophilic nucleotide second messenger generated from ATP by adenylate cyclase. This molecule regulates various signaling pathways through its action on cAMP‐dependent protein kinase A (PKA) [210]. This signaling is essential for biological processes such as cell differentiation, adhesion, apoptosis, survival, and proliferation [211]. Fluctuations in cAMP concentrations can impact neuronal functions, leading to the onset of neurological disorders [212]. The PKA family of serine/threonine kinases exists as a tetramer composed of two catalytic subunits (PKA‐C) and two regulatory subunits (PKA‐R). The PKA‐C subunits contain an active site for ATP binding and hydrolysis, which is required for its kinase activity. PKA is the key mediator of cAMP signaling; consequently, abnormalities in its function result in neurodevelopmental disorders affecting learning, memory, cognition, motor function, coordination, pain perception, and social behavior [213]. The binding of cAMP to the PKA‐R subunit induces a conformational change that results in the release of the PKA‐C subunit, enabling it to phosphorylate target proteins, such as CREB, and activate downstream signaling pathways [214, 215] involved in the regulation of transcription, metabolism, and cell growth, among other cellular activities. The binding of neurotransmitters, hormones, or signaling molecules to GPCRs can alter intracellular cAMP concentrations, resulting in PKA activation. Activated PKA phosphorylates CREB, allowing it to bind to cAMP response elements in target gene promoters and enhancing BDNF transcription. This pathway plays a pivotal role in the neural plasticity associated with neurological disorders. Studies in rats have shown that curcumin mitigates the dichloroacetic acid‐induced synaptic plasticity impairment and neurotoxicity in the hippocampus via the activation of the cAMP/PKA/CREB signaling pathway, thereby improving cognitive, learning, and memory functions, and exerting neuroprotective effects [216]. The 5‐HT1A receptor, located on the somas and dendrites of serotonergic neurons in the dorsal raphe nucleus, influences cAMP/PKA activity postsynaptically [217]. In an in vitro model of cortisol‐induced neuronal injury, curcumin administration reduced neuronal toxicity and enhanced neural plasticity through the modulation of the 5‐HT1A receptor and the subsequent activation of the cAMP/PKA/CREB signaling pathway [218]. Furthermore, research has shown that curcumin derivatives display antidepressant‐like behaviors associated with increased BDNF levels in the hippocampus. For instance, Li et al. found that curcumin increases cAMP/PKA/pCREB/BDNF protein expression in the hippocampus of mice, suggesting that its antidepressant effects are associated with this signaling axis [219]. Collectively, the beneficial effects of curcumin on neurological disorders are attributed to its regulatory influence on the cAMP/PKA signaling pathway.

#### 3.6.3 Wnt/β‐Catenin Signaling Pathway

Substantial evidence supports that the Wnt/β‐catenin pathway is required for normal brain development, particularly for the regulation of neuronal functions within the CNS [220]. In the CNS, the Wnt pathway coordinates several neuronal processes, including differentiation, neurogenesis, synaptogenesis, and neuroprotection [221, 222, 223]. Given that the Wnt/β‐catenin pathway is essential for synaptic plasticity, which is required for cognitive functions such as learning and memory, its dysfunction is associated with the cognitive impairments observed in neurological disorders. Wnt proteins are necessary for synaptogenesis and the modulation of neurotransmission, both pre‐ and postsynaptically [224]. Additionally, the Wnt/β‐catenin pathway is critical for the formation of the blood–brain barrier and the maintenance of its integrity and functionality. Importantly, the disruption of this barrier is a notable pathogenic feature of neurological disorders [225]. In a healthy CNS, Wnt/β‐catenin signaling serves as a microenvironmental sensor that maintains the balance between cell survival and apoptosis [226]. Specifically, the binding of Wnt ligand and their agonists to frizzled receptors and low‐density lipoprotein receptor‐related protein 5/6 (LRP5/6) inhibits the glycogen synthase kinase‐3β (GSK‐3β)‐mediated phosphorylation of β‐catenin and its subsequent proteasomal degradation. This inhibition triggers Wnt pathway activation, allowing stabilized β‐catenin to accumulate in the cytoplasm and subsequently translocate to the nucleus. Here, it activates the expression of Wnt target genes involved in neuronal survival and plasticity, thereby preserving neuronal integrity. Cancer initiation and progression may be driven by genomic mutations. The Wnt signaling pathway is a key regulator of cell growth, differentiation, and migration, and it plays a critical role in promoting tumor proliferation. Studies have shown that Wnt/β‐catenin signaling is a central pathway governing these processes, and its overexpression can prevent chemotherapy‐induced cancer cell death [193]. In recent years, the Wnt signaling pathway has also been recognized as a crucial regulator of cardiac development and homeostasis. Aberrant activation of this pathway can lead to cardiac hypertrophy, fibrosis, and ultimately heart failure. Evidence suggests that inhibition of Wnt signaling may improve cardiac function and reduce complications associated with cardiovascular diseases [227].

Investigations into the therapeutic potential of curcumin in neurological disorders have identified the modulation of Wnt/β‐catenin signaling as a viable pharmacological strategy. Specifically, Lou et al. indicated that curcumin promotes adult neurogenesis in Aβ1–42 mice by activating the Wnt/β‐catenin pathway, an effect that is abolished following the administration of a Wnt/β‐catenin inhibitor [228]. In a model of intermittent hypoxia‐induced neuronal injury, curcumin treatment significantly ameliorated morphological damage in hippocampal neurons and restored proliferative capacity. These effects were accompanied by the activation of the Wnt/β‐catenin signaling pathway and a reduction in both cytotoxicity and tissue damage, suggesting that curcumin exerts a protective role through the modulation of this pathway [229]. Another study demonstrated that curcumin treatment upregulated the protein expression of Wnt and β‐catenin while downregulating that of GSK‐3β, thereby promoting neuronal differentiation, inducing neurogenesis, and reversing cognitive impairments [230]. In an AD model involving the cotransfection of APPswe and BACE1‐mycHis plasmids into SH‐SY5Y neuroblastoma cells, Zhang et al. found that curcumin treatment inhibited GSK‐3β activity. This inhibition led to the activation of the Wnt/β‐catenin signaling pathway and upregulated the protein expression of β‐catenin and cyclin D1, ultimately providing neuroprotection [231]. Additionally, it has been reported that curcumin significantly improves cognitive deficits following cerebral ischemia by upregulating the Wnt/β‐catenin pathway and stimulating neural stem cell proliferation [232]. Combined, these findings imply that curcumin alleviates neurological disorders by modulating the Wnt/β‐catenin signaling pathway. One study demonstrated that curcumin attenuated vascular intimal hyperplasia and restenosis, thereby exerting cardiovascular protective effects, through lncRNA H19‐mediated activation of the Wnt/β‐catenin signaling pathway. Specifically, curcumin reduced the expression of β‐catenin, c‐Myc, and cyclin D1, thereby inhibiting the proliferation and migration of vascular smooth muscle cells [233]. Emerging evidence indicates that curcumin significantly suppresses the proliferation and invasion of cervical cancer cells, induces G2/M phase cell cycle arrest and apoptosis, thereby inhibiting tumor growth and metastasis. These effects are mediated through downregulation of the Wnt/β‐catenin signaling pathway [234]. Yuan et al. found that curcumin effectively ameliorated osteoarthritis and inhibited Wnt/β‐catenin signaling in chondrocytes, suggesting that curcumin may represent a promising therapeutic candidate and a potential new direction for osteoarthritis treatment [235].

In summary, the therapeutic effects of curcumin in Multiple diseases are mediated via a multifaceted profile that includes anti‐inflammatory activity, the modulation of mitochondrial dysfunction, apoptosis regulation, PI3K/AKT and MAPK signaling pathway modulation, antioxidant effects, and enhancement of neural plasticity. Thus, curcumin remains a viable candidate for advancing the treatment of neurological disorders.

## 4 Multiple Strategies for Improving Curcumin Bioavailability

Despite the potential of curcumin to improve neurological disorders, cancer, cardiovascular diseases, inflammatory conditions, and autoimmune diseases, its clinical translation is hindered by negligible water solubility, chemical instability, and rapid systemic metabolism, which severely limit its oral bioavailability [8]. Following oral administration, curcumin exhibits low bioavailability due to the first‐pass metabolism and, to some extent, intestinal metabolism. In particular, curcumin undergoes glucuronidation and sulfation, resulting in the formation of curcumin glucuronide and sulfate conjugates. These metabolic transformations reduce the levels of free curcumin in the circulation, thereby limiting the ability of the parent compound to reach target tissues at therapeutically effective concentrations and exert its therapeutic effects [236]. Studies have reported that the oral administration of 3.6 g of curcumin results in a peak plasma concentration of only 11.1 nmol/L after 1 h [228]. Additionally, although a portion of curcumin is absorbed by the human body, its therapeutic utility is constrained by rapid circulatory clearance and insufficient targeted delivery efficiency. Notably, the prolonged intake of high doses of curcumin may diminish its health‐promoting effects [228]. To address these challenges, advanced drug delivery systems have been developed to optimize the pharmacodynamic profile of the compound. Nanotechnology‐based platforms—including liposomes and various nanoparticles such as protein–polysaccharide (PRO–POL), poly (lactic‐co‐glycolic acid) (PLGA), solid lipid (SLNs), and gold (AuNPs) variants—have demonstrated potential in improving the stability, targeted delivery, and CNS permeating ability of curcumin. Additionally, innovative formulations such as exosomes, micelles, and cyclodextrin inclusion complexes are being explored to optimize the therapeutic effects of curcumin in neurological disorders, cancer, cardiovascular diseases, inflammatory conditions, and autoimmune diseases. The following section reviews strategies for refining curcumin delivery systems, with a particular focus on nanotechnology‐based methods aimed at improving its oral bioavailability (Figure 4).

**FIGURE 4:**
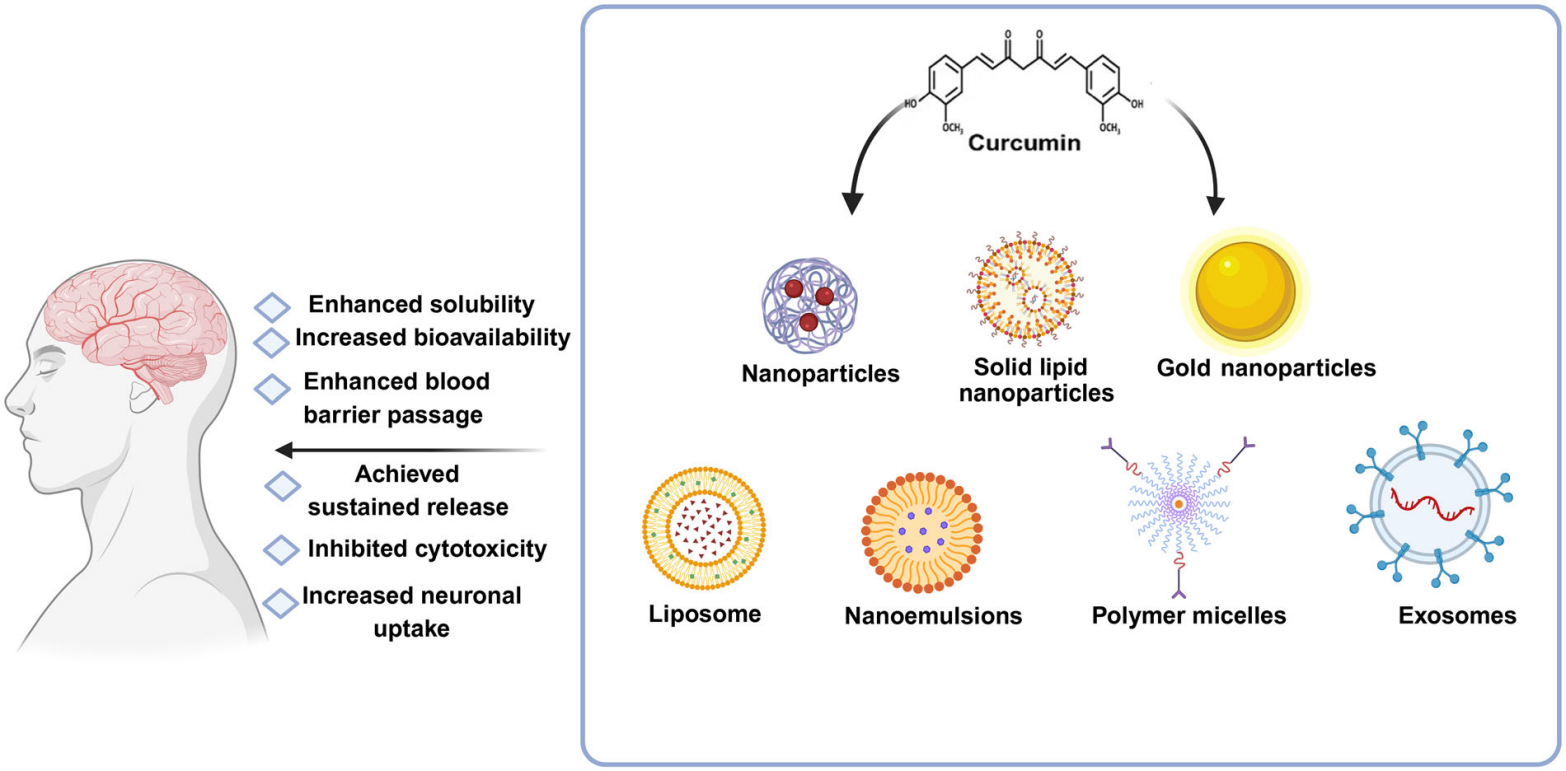
Multiple strategies for improving curcumin bioavailability.

### 4.1 Nanoparticles

#### 4.1.1 Protein–Polysaccharide

Studies have noted that PRO–POL complexes function as effective delivery vehicles for curcumin [8]. Polysaccharides and proteins can be assembled into these carriers via hydrogen bonding, electrostatic interactions, and covalent bonding, which allows for the precise regulation of electrostatic interactions and interparticle repulsion. This structural organization effectively inhibits protein aggregation while enhancing the solubility and stability of the resulting complex [237]. Meng et al. [238] synthesized a poly‐chitosan (CS)–casein hydrophobic peptide conjugate emulsification system employing the Maillard reaction. Their findings indicated that the curcumin‐loaded emulsion exhibited excellent thermal stability, amphiphilic properties, and emulsifying capabilities. Ovalbumin (OVA) is a globular monomeric phospholipid protein abundantly found in egg white. The hydrophobic amino acid residues within OVA bind to the hydrophobic moieties of curcumin, thereby mitigating its low aqueous solubility and associated pharmacokinetic limitations.

In one study, the sol‐oil method was used to prepare curcumin nanoparticles encapsulated within lactoferrin. These nanoparticles enhanced the specificity and efficacy of curcumin in the brain, ultimately augmenting its neuroprotective effects [239]. In other research, Niu et al. prepared curcumin‐encapsulating OVA/carboxymethyl cellulose sodium (CMC) nanoparticles (OVA/CMC NPs) using a combination of complex coacervation and thermal induction. Compared with free curcumin, these OVA/CMC NPs demonstrated superior stability and antioxidant capacity [240]. Furthermore, Yang et al. synthesized CS and BSA nanoparticles (CS–BSA NPs) coated with curcumin for the treatment of AD. Using an in vitro system, they reported that these curcumin‐loaded nanoparticles significantly enhanced the ability of curcumin to cross the BBB, extended the drug's half‐life, and decreased the frequency of administration. These nanoparticles also exhibited anti‐inflammatory and neuroprotective effects [35]. Importantly, leveraging the properties of these CS–BSA NPs may amplify the neural plasticity‐promoting effects of curcumin. In addition, lipid‐modified albumin nanoparticles loaded with curcumin have been shown to increase ROS levels, induce extensive DNA fragmentation and MMP depolarization, thereby promoting apoptosis in MDA‐MB‐231 cells. These nanoparticles also exhibit strong tumor‐suppressive effects in 4T1 tumor‐bearing mouse models [241]. Furthermore, curcumin delivered via albumin‐based nanoparticles has demonstrated significant inhibitory effects on breast cancer cells, highlighting its potential as an effective nanocarrier‐mediated therapeutic strategy [242].

#### 4.1.2 Polylactic Acid‐Glycolic Acid

Polylactic acid‐glycolic acid nanoparticles (PLGA NPs) encapsulating curcumin have been demonstrated to significantly enhance the concentrations of curcumin in splenic and pulmonary tissues. Compared with free curcumin, curcumin‐loaded PLGA NPs (Cur–PLGA NPs) exhibit prolonged retention in the cerebral cortex and hippocampus, with increases of 96% and 83%, respectively [243]. Furthermore, Sanaz et al. reported that Cur–PLGA NPs can mitigate hydrogen peroxide‐induced oxidative stress and prevent GSH depletion, thereby conferring protection against neuronal injury [244]. Paka et al. synthesized GSH‐functionalized nanoparticles (GSH‐NPs) encapsulating curcumin and observed that they displayed significantly enhanced neuroprotective efficacy compared with free curcumin. These nanoparticles evaded lysosomal degradation and enhanced the absorption of the formulation [245]. In another strategy, curcumin‐loaded PLGA NPs were incorporated into a three‐dimensional (3D)‐printed scaffold composed of sodium alginate and gelatin. This formulation was designed for rapid sublingual dissolution, thereby bypassing hepatic first‐pass metabolism, which ordinarily limits bioavailability. The formulation also provides improved stability and reduced dosing frequency. Notably, PLGA NPs exhibit no cytotoxicity and exert significant neuroprotective effects, highlighting their therapeutic potential in neurological disorders [246]. Fan et al. engineered brain‐targeting nanoparticles by conjugating PLGA–polyethylene glycol (PEG) with B6 peptides for curcumin encapsulation. These nanoparticles reduced curcumin particle size, enhanced cellular uptake, and demonstrated excellent blood compatibility. Compared with free curcumin, PLGA–PEG–B6–curcumin nanoparticles significantly improved spatial learning and memory in mice [247].

One study introduced H‐ferritin nanocages (HFn) as a novel nanoformulation for curcumin delivery. The authors reported that HFn–curcumin exhibited superior water dispersibility, lacked cytotoxicity, and effectively penetrated the BBB. In mice, this formulation significantly inhibited microglial proliferation and was associated with improved cognitive function [248]. Anial et al. developed water‐soluble PLGA‐coated curcumin nanoparticles conjugated with Tet‐1 peptides. The resulting PLGA–Tet‐1–curcumin nanoparticles effectively disrupted amyloid aggregation, demonstrated strong antioxidant activity without cytotoxic effects, and showed significantly enhanced neuronal uptake. Consequently, these PLGA–Tet‐1–curcumin nanoparticles constitute a promising strategy for targeted drug delivery to specific brain regions [249]. Nanoparticles used as drug delivery systems can facilitate drug penetration across the BBB through surface modification or functional coating. Heba et al. demonstrated that combining curcumin with zinc oxide nanoparticles (ZnONPs) significantly improved its pharmacokinetic profile and solubility. Compared with curcumin alone, curcumin–ZnONPs exhibited notably enhanced antioxidant activity [250]. Furthermore, the encapsulation of curcumin within PLGA nanoparticles shields it from environmental degradation, including enzymatic breakdown and pH‐related instability, thereby substantially improving its bioavailability, solubility, and chemical stability [251]. Researchers have developed curcumin‐loaded biocompatible PLGA nanoparticles to improve the bioavailability and therapeutic efficacy of curcumin, thereby enhancing its antitumor activity. The results showed that after administration of curcumin nanoparticles, the inhibition rate in gastric cancer cells increased from 7% within 24 h to 69%. In addition, compared with native curcumin, nano‐formulated curcumin significantly increased apoptosis after 72 h of treatment [252]. Leila et al. [253] prepared curcumin‐loaded PLGA nanoparticles using an oil‐in‐water (O/W) emulsion solvent diffusion method. The nanoparticles were subsequently coated with polyethyleneimine and complexed with Bcl‐2 siRNA. The formulations were characterized in terms of zeta potential, particle size distribution, and drug encapsulation efficiency. The results demonstrated enhanced cytotoxicity against T47D cells and reduced Bcl‐2 gene expression, indicating a synergistic anticancer effect between siRNA‐mediated gene silencing and curcumin. Sindhu et al. [254] encapsulated curcumin in a PLGA‐based drug delivery system for targeted delivery to atherosclerotic plaques. The Cur–PLGA nanoparticles exhibited good biocompatibility with THP‐1 monocyte‐derived macrophages and reduced intracellular cholesterol content, with 250 µg/mL identified as the optimal concentration. In addition, Cur–PLGA nanoparticles effectively inhibited the formation of atherosclerotic foam cells. Another study synthesized PLGA atorvastatin curcumin (PLGA–AT–Cur) conjugated nanoparticles. In vivo results demonstrated that oral administration of PLGA–AT–Cur nanoparticles reduced the progression of atherosclerotic lesions in mice. Compared with free curcumin, PLGA–AT–Cur nanoparticles decreased aortic lipid accumulation, oxidative stress, and serum high‐density lipoprotein levels. These findings suggest that PLGA–AT–Cur nanoparticles may have potential therapeutic benefits in cardiovascular disease [255]. In a further study, curcumin and short‐chain fatty acids were coencapsulated in PLGA nanoparticles (Cur–PLGA NPs@SCFAs), achieving an encapsulation efficiency of 66% and sustained release over 48 h. In vitro, Caco‐2 cell assays showed good cytocompatibility, with cell viability exceeding 81.5% at 40 µg/mL. In vivo, high‐dose treatment significantly improved disease symptoms, restored colon length and spleen index, and reduced inflammatory cytokine levels. These findings indicate that the nano‐delivery system provides significant protective effects against intestinal inflammation and barrier dysfunction [256].

#### 4.1.3 Solid Lipid Nanoparticles

Solid lipid nanoparticles (SLNs) are drug delivery systems developed in the early 1990s [229]. Structurally, SLNs consist of a solid hydrophobic core surrounded by a phospholipid monolayer, with surfactants incorporated to enhance stability. Unlike polymeric nanoparticles, SLNs are mainly composed of physiologically compatible lipid components, which reduces the risk of acute and chronic toxicity [229]. Additionally, SLNs exhibit greater physicochemical stability and lower rates of drug leakage issues than liposomes [229]. Curcumin‐loaded SLNs (Cur–SLN) were prepared using a microemulsion technique, yielding an average particle size of 136.4 nm and a drug loading capacity of 10%, allowing for sustained curcumin release over 7 days. Pharmacokinetic evaluation in rats demonstrated that orally administered Cur–SLN achieved significantly higher bioavailability than free curcumin [230].

Ganesan et al. assessed the efficacy of Cur–SLN in BV‐2 microglial cells under LPS‐induced neuroinflammatory conditions. Compared with conventional curcumin, Cur–SLN more effectively reduced nitric oxide and proinflammatory cytokine levels in a dose‐dependent manner. These findings highlight the potential of SLNs to serve as a delivery platform for curcumin in the context of microglia‐mediated neuroinflammation [231]. Furthermore, the intraperitoneal administration of curcumin‐encapsulated SLNs in AD model mice resulted in blood–brain barrier penetration, enabled the modulation of tissue transglutaminase (TG2) subtype expression, promoted cell repair, and reduced neuroinflammation, thereby improving cognitive dysfunction [232]. In an aluminum‐induced AD mouse model, Cur–SLN effectively attenuated aluminum‐induced neurotoxicity by inhibiting lipid peroxidation, restoring antioxidant enzyme activity, and reducing acetylcholinesterase (AChE) levels. Behavioral assessments demonstrated that Cur–SLN (1–50 mg/kg) improved spatial memory more effectively than free curcumin (50 mg/kg). Moreover, Cur–SLN inhibited lipid peroxidation by 97.46% and reduced AChE levels to 73% of the model peak, significantly exceeding the effects observed with free curcumin. These findings suggest that SLNs efficiently preserve and deliver bioactive curcumin, achieving therapeutic effects at substantially lower doses than unencapsulated curcumin [257]. Huang et al. prepared Cur–SLN using an emulsification‐evaporation method, and found that it enhanced both curcumin dispersibility and brain delivery efficiency while alleviating oxidative stress‐induced neuronal apoptosis, thus demonstrating neuroprotective effects [161]. In another study, a temperature‐sensitive Cur–SLN in situ nasal gel was developed for intranasal delivery targeting AD. The formulation demonstrated appropriate particle size, high encapsulation efficiency, and sustained‐release characteristics. In vitro studies revealed that loading curcumin onto nanocarriers and gel formulations reduced its free radical scavenging rate while facilitating prolonged drug release and enhancing brain bioavailability [258]. Prabhu et al. further employed Cur–SLN‐loaded composite microneedles to improve curcumin bioavailability and therapeutic efficacy. Cur–SLN was prepared via a microemulsion method using glyceryl monostearate as the lipid matrix and Tween 80 as a stabilizer. The results indicated that the excipients exhibited good compatibility with curcumin and produced no skin irritation. Furthermore, the formulation significantly improved motor deficits in an animal model of PD, enhancing motor coordination and balance. These results provide a theoretical basis for the development of novel Cur–SLN formulations for the treatment of neurological disorders [259]. Another study reported that Cur–SLNs exhibited small and uniform particle size, high encapsulation efficiency, favorable in vitro sustained‐release profiles, and excellent stability. Compared with free curcumin, Cur–SLNs significantly enhanced cellular uptake efficiency, thereby improving the inhibition of proliferation, migration, and invasion of MHCC‐97H cells, while also increased apoptosis [260]. Vinit et al. [261] evaluated the anticancer effects of curcumin‐loaded SLNs conjugated with concanavalin A (ConA) in A549 human lung cancer cells. Cur–SLNs improved the recognition and uptake by lung cancer cells, thereby enhancing the inhibitory effect of curcumin on A549 cell proliferation. These findings suggest that Cur–SLNs may serve as a potential targeted drug delivery system for lung cancer therapy. Arora et al. [262] investigated the protective effects of curcumin and Cur–SLNs in complete Freund's adjuvant‐induced rat model of arthritis. The results showed that arthritic rats exhibited significantly reduced paw withdrawal thresholds and shortened hot‐plate response time. In addition, peripheral white blood cell counts, oxidative/nitrosative stress levels, tumor necrosis factor‐α (TNF‐α), C‐reactive protein (CRP), and radiographic changes in the tibio‐tarsal joint were markedly increased. Compared with free curcumin, Cur–SLNs significantly alleviated arthritic symptoms, improved biochemical parameters, preserved radiological joint integrity, and demonstrated dose‐dependent therapeutic effects. Another study evaluated the potential of Cur–SLNs for the treatment of sepsis. Mice were pretreated with either free curcumin or Cur–SLNs prior to intraperitoneal LPS administration. Compared with free curcumin, Cur–SLNs more effectively reduced the expression of proinflammatory cytokines IL‐1β, IL‐6, and TNF‐α, while enhanced the expression of the anti‐inflammatory cytokine IL‐10. Moreover, Cur–SLN treatment significantly alleviated sepsis‐induced organ failure [263]. In addition, a Cur–SLN hydrogel was developed for the topical treatment of skin inflammation. The formulation exhibited small particle size, high encapsulation efficiency, and good stability, as well as a certain occlusive and moisturizing effect. In vitro release and ex vivo permeation studies demonstrated that the Cur–SLN hydrogel significantly improved drug release, skin penetration, and skin retention compared with free curcumin gel. In a mouse wound inflammation model, Cur–SLNs markedly promoted wound healing, reduced lipid peroxidation levels, and increased antioxidant markers such as GSH and SOD. Histopathological analysis further confirmed superior anti‐inflammatory and tissue‐repair effects compared with free curcumin gel [264].

#### 4.1.4 Gold Nanoparticles

AuNPs exhibit unique properties owing to their small size and distinctive structure. The diameter of AuNPs typically ranges from 1 to 100 nm, enabling them to interact with biomolecules at the cellular and molecular levels. Additionally, AuNPs are recognized for their excellent biocompatibility and relative inertness, which minimizes the risk of adverse immune reactions or cytotoxicity when used in biological systems [265]. They can be functionalized with various molecules, such as targeting ligand neurological disorders, antibodies, or drugs, allowing for their customization for specific applications, thereby enhancing their targeting and therapeutic capabilities [266] and offering promising strategies for optimizing drug delivery and improving treatment outcomes.

Zhou et al. modified AuNPs with peptides via gold‐sulfur, creating peptide‐functionalized AuNPs that selectively bind copper ions, facilitating the formation of Aβ–copper complexes, thereby mitigating cellular toxicity [267]. Notably, advanced delivery systems have been developed to enhance the neuroprotective effects of curcumin in neurological disorders. For instance, one study evaluated the activity of curcumin against CNS amoebic infections by coupling curcumin with AuNPs (Cur–AuNPs). This formulation resulted in a 56% increase in the inhibition of amoebic activity relative to free curcumin, with no detectable cytotoxicity at concentrations of 5 and 10 µM. The authors proposed that curcumin–AuNPs represent a novel potential therapeutic agent for treating cerebral amoebic infections [268]. Moreover, low‐intensity pulsed ultrasound combined with Cur–AuNPs was found to exert neuroregulatory effects in an Aβ‐induced AD mouse model. Cur–AuNPs traversed the blood–brain barrier without inducing hepatic toxicity. Compared with standalone curcumin therapy, Cur–AuNPs effectively reduced the levels of nitrite in the hippocampus and cortex and downregulated NF‐κB expression in the hippocampus [269]. Zeynab et al. [270] reported a Cur–AuNPs and evaluated its biological effects in a doxorubicin‐induced acute toxicity model in BALB/c mice. The results demonstrated that Cur–AuNPs exerted significantly stronger cardioprotective effects than free curcumin. After 24 h of intervention, Cur–AuNPs reduced serum biomarkers of myocardial injury and apoptotic proteins, increased the antiapoptotic protein Bcl‐2, and showed no evidence of interstitial hemorrhage or cellular gaps in myocardial histology. Previous studies have shown that in a chicken embryo oxidative stress model, 300 µg/mL of Cur–AuNPs significantly reduced cardiac lipid peroxidation and ROS levels, while restored the activity of antioxidant enzymes such as SOD and catalase (CAT), suggesting that curcumin alleviated acute cardiotoxicity by modulating oxidative stress [271]. In another study, curcumin was used as both a reducing and stabilizing agent, and Cur–AuNPs were integrated into a conductive nanocomposite hydrogel to develop a biocompatible and multifunctional porous nanocomposite material. In vitro antioxidant assays demonstrated that Cur–AuNPs exhibited dose‐dependent free radical scavenging activity, reaching a peak at 50 µg/mL. In an isoproterenol‐induced myocardial injury rat model, Cur–AuNP administration significantly alleviated cardiac damage, accompanied by reductions in inflammation, oxidative stress, and infarct size. These findings indicate that curcumin‐mediated AuNPs possess strong therapeutic potential in cardiovascular diseases [272]. Wang et al. combined the anticancer properties of curcumin with AuNPs and encapsulated them within a ZIF‐8 framework to construct a composite drug delivery system (Cur/Au@ZIF‐8). The drug loading efficiency of curcumin in this system reached 47.16%, and the release profile showed a faster release under conditions of pH 5.5, with a cumulative release rate of 72.34%. In a rat hepatocellular carcinoma model, Cur/Au@ZIF‐8 exhibited enhanced antitumor efficacy, achieving a tumor inhibition rate of 46.81% [273]. In addition, an injectable decellularized ECM scaffold combined with AuNPs was developed. In vitro evaluations using L929 mouse fibroblasts and human chondrocytes demonstrated good biocompatibility, ROS scavenging capacity, and chondroprotective effects. Under IL‐1β stimulation, the system effectively reduced intracellular ROS levels and alleviated apoptosis in chondrocytes [274].

### 4.2 Liposomes

Liposomes are specialized vesicles typically composed of cholesterol and phospholipids, among other substances, capable of encapsulating therapeutic agents. They serve as versatile platforms for biotherapy and for overcoming bioavailability constraints. Liposomes can encapsulate a variety of pharmacological agents, protecting them from degradation while enhancing pharmacokinetics and minimizing adverse reactions. These advantages stem from their particle size, lipid bilayer structure, and inherent biocompatibility. Consequently, liposome‐based delivery systems are among the most extensively investigated methodologies in modern pharmacology, with significant applications in the treatment of neurological disorders, oncology, and pain management [275]. Advancements in liposomal technology have enabled the evolution of liposomal platforms from passive to active targeting systems, while conventional liposomes are being transformed into long‐circulating formulations. Recently, numerous modified liposomes constructed from specific materials have been designed to achieve targeted delivery across the blood–brain barrier and for intervention in key signaling pathways or targets. In clinical research, several novel liposomal drug delivery methods have been approved for therapeutic use, offering new avenues for the treatment of neurological disorders.

In applications within the nervous system, surface modifications of liposomes primarily aim to overcome the challenges posed by the blood–brain barrier [276]. The blood–brain barrier is a physiological barrier formed by brain microvascular endothelial cells, astrocytes, and neurons that effectively prevents most drugs from entering the CNS, thereby maintaining neurological stability. However, this barrier also hinders the efficient delivery of treatments for neurological disorders, often resulting in poor efficacy or increased side effects. Liposomes are self‐assembled colloidal structures featuring an aqueous core surrounded by single or multiple lipid bilayers, synthesized through a variety of methods, including ethanol injection, thin‐film hydration, and extrusion [277, 278]. Their amphiphilic nature allows for the encapsulation of hydrophilic drugs in the aqueous core and hydrophobic drugs within the lipid layers, thus broadening the range of drug delivery strategies for ND treatment [279]. Seeking to enhance the therapeutic effects of curcumin in neurological disorders, Chen et al. delivered curcumin to the brain via NLCs and reported that it significantly extended its half‐life from 3.1 to 5.7 h and increased curcumin plasma concentrations by 6.4‐fold [280]. Further investigations involving the incorporation of curcumin into NLCs (CUR–NLCs) revealed that, compared with rats given free curcumin, those administered these NLCs demonstrated enhanced neuronal survival in the cortex and hippocampus, along with significant inhibition of neuroinflammation through the downregulation of proinflammatory factors in the brain. These outcomes can be attributed to the ability of CUR–NLCs to cross the BBB and efficiently deliver curcumin to the brain, thereby enhancing its neuroprotective effects [281]. Similarly, research involving curcumin encapsulated in liposomes containing cardiolipin (CL) and wheat germ agglutinin (WGA) (WGA–CL–CUR) showed that this formulation significantly enhanced the ability of curcumin to traverse the BBB while also mitigating potential curcumin‐induced cytotoxicity and attenuating neuronal apoptosis [282]. Soroor et al. demonstrated that encapsulating curcumin within SLNs and NLCs significantly enhanced the uptake of curcumin into the brain, resulting in a greater than fourfold increase in curcumin absorption [283]. Meanwhile, Yan et al. encapsulated curcumin into lipid–PLGA nanobubbles using the melt crystallization method, yielding curcumin‐loaded lipid–PLGA nanobubbles (Cur–NBs). They noted that these nanobubbles were effective at releasing curcumin, achieving a release rate of up to 30% within 6 h. Furthermore, the Cur–NBs were capable of penetrating the BBB, enabling curcumin to reach the brain core, while also significantly enhancing its solubility [284]. Li et al. [285] developed E‐selectin and intercellular adhesion molecule‐1 (ICAM‐1) targeted liposomes (T‐AC–Lipo) for curcumin delivery to enhance drug targeting and antiatherosclerotic efficacy. Therapeutic effects were evaluated in both in vitro endothelial inflammatory model and in vivo ApoE^−^/^−^ atherosclerosis model. The T‐AC–Lipo formulation significantly reduced the percentage of aortic atherosclerotic lesions and decreased inflammatory cytokine levels and lipid profiles. In addition, T‐AC–Lipo increased intracellular curcumin concentration and effectively reduced cellular cytotoxicity. Zhu et al. [286] prepared curcumin‐loaded liposomes (Cur–Lips) using a microfluidic chip technique, achieving an encapsulation efficiency of 99.33%. Cur–Lips increased skin penetration by 2.76‐fold, which further increased to 4.48‐fold when combined with the transdermal peptide TD‐1. This delivery system enhanced drug permeation by interacting with stratum corneum lipids and keratin, increasing hydration, and facilitating transport via hair follicles and sweat glands. In vitro and in vivo studies confirmed that the TD‐1 + Cur–Lips hydrogel significantly inhibited melanoma growth and induced apoptosis, providing a safe and effective strategy for localized skin cancer therapy. In another study, Narayanan et al. encapsulated curcumin in liposomes and evaluated its chemopreventive effects in a male B6C3F1/J mouse model of prostate cancer. The results showed that the liposomal formulation significantly increased curcumin concentrations in serum and prostate tissue. In vitro experiments demonstrated that curcumin effectively inhibited cell proliferation and induced apoptosis, and in vivo studies further confirmed significant suppression of prostate tumor growth [287]. Dyandevi et al. developed a curcumin–piperine liposomal hydrogel for the topical treatment of atopic dermatitis. Ex vivo skin permeation studies showed a drug retention rate of 74.23 ± 1.12% in the skin, with minimal transdermal penetration, supporting its suitability for local therapy. Antioxidant assays demonstrated strong free radical scavenging activity. Compared with free curcumin, the liposomal hydrogel significantly reduced erythema, scratching behavior, ear thickness, and spleen index. Overall, the curcumin liposomal hydrogel exhibited synergistic antioxidant and anti‐inflammatory effects [288].

### 4.3 Nanoemulsions

NEs are colloidal dispersion systems composed of two immiscible liquids stabilized by ionic or nonionic surfactants, with dispersed droplet diameters ranging from 20 to 500 nm [289]. Surfactants are frequently utilized in NE structures to prevent aggregation via electrostatic and steric stabilization [290]. Within these systems, the nano‐sized droplets display sustained Brownian motion, which enables them to counteract gravitational effects and enhances resistance to droplet coalescence, thus maintaining kinetic stability. However, NEs also exhibit thermodynamic instability, primarily attributed to Ostwald ripening, a process where smaller droplets gradually enlarge and promote phase separation, consequently affecting long‐term dispersion stability. Generally, an NE comprises three components: oil, water, and emulsifiers, mixed in appropriate ratios to form either simple or multilayer structures. Simple NEs are further categorized into O/W or water‐in‐oil (W/O) emulsions [291], while multilayered NEs contain an additional internal droplet within the continuous phase, resembling the dispersed medium, which can be either O/W/O or W/W. Hence, the composition of the system plays is the primary determinant of the resulting NE type. Compared with other nano‐drug delivery systems, NEs offer distinct advantages, such as high solubility, flexibility in composition, ease of preparation, rapid drug release, high stability, uniform particle size, and high drug loading capacity [292].

Curcumin is a lipophilic molecule, and it displays low aqueous solubility and limited oral bioavailability. Consequently, NEs serve as a unique pathophysiological‐targeted drug delivery system for neurological disorders, offering new solutions to current therapeutic challenges. Research has shown that curcumin NEs (Cur–NEs) improve neurological function scores in rat models of cerebral I/R injury, restore redox homeostasis in the brain, reduce the expression of inflammatory factors, and attenuate cell apoptosis, thereby effectively alleviating neuronal damage [293]. Sheng et al. developed a Cur–NE using emulsification methods to enhance the water solubility and oral bioavailability of curcumin. Using an animal model of CUMS, they assessed the antidepressant activity of Cur–NE and subsequently analyzed its pharmacokinetic behavior. The optimization of Cur–NE yielded uniformly small particle sizes, which significantly improved the pharmacokinetics of curcumin in rats. Furthermore, Cur–NE notably ameliorated depressive and anxiety‐like behaviors in CUMS model mice, exerting antidepressant effects through the modulation of the serotonergic system [294]. Another study found that the relative bioavailability of Cur–NE reached 11.18 times that of a curcumin suspension, enhancing both the solubility and stability of curcumin, and representing a promising drug delivery system [295]. Additionally, Manoj et al. prepared nano‐emulsified curcumin droplets, which significantly improved curcumin's solubility and oral absorption rates and improved memory and cognitive abilities in mice while reducing serum cortisol levels [296]. A study evaluated the protective effects of curcumin‐loaded NEs against doxorubicin‐induced cardiotoxicity. After encapsulation of curcumin in 100 nm NEs, cardiomyocyte viability increased by approximately 35–40%. In addition, lipid peroxidation products and inflammatory cytokine levels were significantly reduced, while the expression of the anti‐inflammatory cytokine IL‐10 was increased [297]. Bharmoria et al. developed a gelatin‐coated olive O/W NE encapsulating curcumin (G‐Cur–NE). This formulation effectively inhibited curcumin autoxidation and significantly improved its stability and cellular uptake. In MDA‐MB‐231 triple‐negative breast cancer cells, G‐Cur–NE selectively induced cancer cell death while exhibited low cytotoxicity toward normal L929 cells, demonstrating its potential as a safe and efficient nanoplatform for breast cancer therapy [298]. Another study investigated a NE codelivery system containing FU and curcumin (CU/FU–NE). The results showed that the area under the curve (AUC_0‐t_) of CU/FU–NE was 8.85‐fold and 8.59‐fold higher than that of curcumin (CU) and FU, respectively. The IC_50_ values of CU/FU–NE against HepG2 cells were 4.6‐ and 4.9‐fold lower than those of CU and FU. In vivo antitumor studies demonstrated a tumor inhibition rate of 49.29% for CU/FU–NE, compared with 24.84% for CU and 4.72% for FU. These findings indicated that CU/FU–NE exhibited enhanced efficacy and reduced toxicity, representing a promising anticancer formulation [299]. Lei et al. prepared a curcumin/emodin NE (Cur/EMO NE), which was subsequently coated with a CS–sodium alginate crosslinked layer. In vitro release studies demonstrated pH‐responsive controlled release behavior. Pharmacodynamic studies in vitro and in vivo showed that Cur/EMO NE primarily targeted macrophages, exerting anti‐inflammatory effects by inhibiting proinflammatory cytokine secretion and promoting anti‐inflammatory cytokine production. In addition, Cur/EMO NE exhibited favorable intestinal mucosal repair properties by upregulating the expression of tight junction proteins ZO‐1 and occludin, thereby improving epithelial barrier integrity [300].

### 4.4 Polymer Micelles

Amphiphilic block copolymers self‐assemble into micelles in aqueous solutions through hydrophobic interactions, serving as carriers for insoluble therapeutic agents. These structures comprise a hydrophobic core and a hydrophilic shell, and their size and shape can be adjusted by varying the length and proportion of the respective hydrophilic and hydrophobic segments [301]. The hydrophobic core enhances the solubility of hydrophobic drugs, while the hydrophilic shell forms extensive hydrogen bonds with water molecules in solution, creating a protective and compact exterior that reduces protein adsorption and cellular adhesion. Notably, micelles are less likely to be recognized and captured by the endothelial reticular system in the bloodstream, thereby prolonging their circulation time. These properties render them one of the most promising nano‐drug carrier systems currently available. Studies have explored the applicability of triblock copolymers as polymeric nanocarriers for passive targeting to the brain. Li et al. prepared amphiphilic triblock copolymer (PLA–PEG–PLA) micelles loaded with curcumin via film hydration methods and subsequently investigated their neuroprotective effects following bilateral common carotid artery occlusion. The resulting micelles displayed a spherical morphology with a hydrodynamic radius of 96.67 nm and a polydispersity index (PDI) of 0.167, indicative of uniform dispersion and good solubility. Whereas free curcumin exhibited rapid dissolution and diffusion, curcumin was slowly released from the micelles. Thus, PLA–PEG–PLA micelles may serve as an effective system for the sustained release of curcumin in the brain [302]. In a separate study, deoxycholic acid–polyethylenimine‐modified micelles were prepared to encapsulate curcumin in a hydrophobic core, yielding small, stable, and nontoxic carriers, while achieving brain‐targeted delivery and significantly enhancing curcumin's water solubility and cellular delivery efficiency [303]. Stephanie et al. compared the impact of natural curcumin and curcumin micelles on mitochondrial function in NMRI mice, PC12 cells, and isolated mouse mitochondria. Their findings revealed that micellar encapsulation achieved a 10–40‐fold increase in curcumin concentrations in mouse plasma and brains compared with that observed for the unencapsulated compound [304]. Similarly, an amphiphilic DSPE–PEG2000 block copolymer was used as a carrier to prepare curcumin polymer micelles via solvent evaporation, yielding micelles with sizes ranging from 70 to 110 nm exhibiting good stability and low cytotoxicity [305]. Kang et al. developed an acid‐responsive curcumin‐loaded polymeric micelle system that could rapidly release curcumin and restore its fluorescence in the acidic microenvironment of osteoarthritis. The curcumin micelles significantly protected joint structures and reduced ECM degradation by inhibiting TNF‐α and IL‐1β, showing superior efficacy compared with free curcumin. This pathology‐triggered responsive nanoplatform provides a new strategy for anti‐inflammatory therapy in osteoarthritis [306]. Carlson et al. [307] prepared curcumin‐loaded polymeric micelles using a solvent casting method. The resulting micelles enabled stable and uniform dispersion of curcumin and exhibited favorable controlled‐release properties. In cardiomyocytes, they showed a pronounced antagonistic effect against toxicity, reducing ROS levels and Caspase‐3/7 activity, thereby alleviating doxorubicin‐induced cardiac injury. Another study employed a one‐step solid dispersion method to encapsulate curcumin into monomethyl poly (ethylene glycol)–poly(ε‐caprolactone)–poly (trimethylene carbonate) (MPEG‐P(CL‐co‐TMC)) micelles. The obtained curcumin micelles had a small particle size of 27.6 ± 0.7 nm and a low PDI of 0.11 ± 0.05, with a drug loading of 14.07 ± 0.94% and an encapsulation efficiency of 96.08 ± 3.23%. In vitro experiments showed that, compared with free curcumin, the micelles significantly enhanced cellular uptake in CT26 cells and increased apoptosis induction. In vivo, the micelles more effectively inhibited the growth of subcutaneous CT26 colon tumors, including suppression of tumor proliferation and angiogenesis, while promoted tumor cell apoptosis. In addition, the curcumin‐loaded micelles exhibited low systemic toxicity [308].

### 4.5 Exosomes

Exosomes are endogenous nanovesicles composed of a natural lipid bilayer rich in adhesion proteins that can interact with cell membranes [309]. Initially, exosomes were thought to be responsible for carrying waste produced by cells; however, they are now known to serve as essential mediators of intercellular communication, transporting functional genetic information and proteins between cells. Notably, their significance in the treatment of neurological disorders is now widely recognized. Given their cellular origin, exosomes possess inherent biocompatibility and degradability, which minimizes the risks of immune rejection and adverse reactions, making them a safer drug delivery option than synthetic carriers [310, 311]. Moreover, their lipid bilayer structure confers stability while also enabling them to cross the blood–brain barrier for the targeted delivery of small‐molecule drugs to the nervous system [312]. The protective potential of miRNA‐126‐modified exosomes following cerebral ischemic injury has been explored in a rat model of MCAO. The authors injected miRNA‐126 exosomes into MCAO model rats, resulting in enhanced neurogenesis and angiogenesis alongside the suppression of microglial activation and inflammatory responses [313]. These findings indicate that miRNA‐126‐enriched exosomes may contribute to the regulation of neurogenesis and neuroinflammatory processes. Mohammad et al. assessed the effects of curcumin‐loaded exosomes on streptozotocin (STZ)‐induced learning and memory impairments and neuroinflammation in rats. Compared with untreated mesenchymal stem cell‐derived exosomes, curcumin‐pretreated exosomes significantly improved memory and learning performance, reduced neuronal degeneration, and more effectively promoted microglial polarization toward the M2 phenotype [314]. In another study, investigators developed human endometrial stem cell‐derived exosome formulations loaded with curcumin, which exhibited uniform particle size and morphology, good stability, and sustained release characteristics. In a PD mouse model, these curcumin exosome formulations were able to cross the blood–brain barrier, ameliorated motor coordination deficits in mice, and enhanced the neuroprotective effects of curcumin against neuronal loss and α‐Syn aggregation [315]. Ding et al. further engineered exosomes modified with DSPE–PEG2000‐C3 peptides and triphenylphosphine (TPP) to encapsulate curcumin (C3/TPP–EXO–Cur) and administered them intravenously to tauP301S transgenic mice. C3/TPP–EXO enabled the targeted delivery of curcumin to neuronal mitochondria, thereby enhancing intracellular drug uptake efficiency. In vivo experiments demonstrated that C3/TPP–EXO–Cur treatment improved memory deficits in tauP301S mice by suppressing tau phosphorylation and inhibiting the expression of proapoptotic genes, providing a novel and potentially effective therapeutic strategy for AD [316]. Zhang et al. constructed neutrophil‐derived exosomes capable of selectively targeting the brains of AD mice in response to inflammatory signals. This platform facilitated the selective intracellular release of therapeutic agents, including curcumin, in the presence of elevated levels of MMP‐2 in the AD microenvironment. In vivo studies demonstrated that curcumin‐loaded exosomes significantly improved memory and cognitive performance in AD mice and inhibited mitochondrial autophagy, thereby restoring mitochondrial metabolic functions in neurons, alleviating mitochondrial dysfunction, and delaying the progression of AD [317]. In addition, curcumin was incorporated into immature DC‐derived exosomes, and this formulation significantly reduced α‐Syn expression, increased neuronal survival, and markedly improved motor performance in PD model mice, thus demonstrating pronounced neuroprotective effects [318]. Sun et al. [319] demonstrated that curcumin encapsulated within exosomes can be preferentially taken up by inflammation‐activated monocyte‐derived myeloid cells. Exosomal delivery significantly improved the solubility, stability, and in vivo bioavailability of curcumin. This system markedly suppressed the expression of proinflammatory cytokines IL‐6 and TNF‐α, reduced inflammatory cell infiltration, and conferred protection in the LPS‐induced septic shock mouse model, ultimately improving survival. These findings suggest that curcumin‐loaded exosomes can target inflammatory cells and enhance anti‐inflammatory activity, providing a novel therapeutic strategy for inflammation‐related diseases. He et al. [320] evaluated the therapeutic effects of curcumin‐loaded mesenchymal stem cell‐derived exosomes (Curc–Exos) on RA synovial fibroblasts (HIG‐82). The exosomal formulation enhanced cellular uptake and stability, significantly inhibited LPS‐induced cell proliferation, and promoted apoptosis. In addition, it downregulated antiapoptotic proteins IAP1 and IAP2, as well as inflammatory mediators including IL‐6, TNF‐α, MMP‐1, and PGE2. This strategy provides a new direction for targeted anti‐inflammatory delivery of curcumin in RA. In recent years, exosomes have emerged as important mediators in cancer therapy. Xue et al. developed engineered exosomes comodified with hyaluronidase PH20 (Exos‐PH20) and curcumin to achieve targeted drug delivery and remodel the tumor microenvironment, thereby enhancing tumor penetration. Rather than eliminating CAFs, this system aimed to “normalize” them and enhance antitumor efficacy. The results showed that Exos‐PH20@Cur leveraged the tumor‐targeting ability of exosomes and PH20‐mediated hyaluronan degradation to disrupt crosstalk between CAFs and cancer cells mediated by TGF‐β and IL‐6, thereby suppressing tumor invasion [321].

Overall, significant progress has been made regarding the use of nanomedicine to overcome the inherent pharmacological limitations of curcumin, including its low aqueous solubility, rapid systemic metabolism, and poor gastrointestinal stability. These delivery systems have substantially improved the therapeutic utilization and efficacy of the compound. However, research focused on improving brain‐targeted delivery efficiency remains relatively limited. There is an urgent need to develop specialized carriers with higher drug‐loading capacities and enhanced delivery efficiency [322]. Furthermore, many existing platforms are designed exclusively for curcumin as a monotherapy, which may partially account for their limited therapeutic efficacy. Combining curcumin with other natural compounds or drugs could represent a promising strategy for enhancing therapeutic outcomes. Additionally, the selection of different administration routes can significantly influence the therapeutic efficacy of curcumin by modulating its pharmacokinetic profile. Consequently, the development of disease‐specific drug‐loading and administration strategies is critical for maximizing the bioavailability and neuroprotective potential of curcumin [8]. Table 1 summarizes the strategies employed for the generation of drug delivery systems aimed at enhancing curcumin bioavailability.

**TABLE 1:** Strategies for enhancing the bioavailability of curcumin.

Curcumin carrier	Packaging material	Improvement strategy	Application effect	References
Nanoparticles	CS–CHP conjugates	Loading curcumin into CS–CHP conjugate emulsifier via Maillard reaction	Improved the thermal stability and solubility of curcumin Sustained release of curcumin	[237]
	Lactoferrin NPs	Curcumin‐loaded lactoferrin NPs	Increased intracellular drug uptake and retention time Enhanced the neuroprotective activity of curcumin	[238]
	OVA–CMC NPs	Preparation of curcumin‐loaded OVA/CMC NPs via complex coacervation and thermal induction	Improved the water solubility of curcumin Enhanced the stability and dispersibility of curcumin Significantly enhanced the antioxidant capacity of curcumin	[239]
	CS–BSA NPs	Encapsulation of curcumin in CS–BSA NPs	Increased the ability of curcumin to cross the BBB Prolonged the half‐life of curcumin and reduced the dosing frequency Promoted the activation of microglia and exerted anti‐inflammatory and neuroprotective effects	[35]
	HSA–DOPE conjugate	Self‐assembly of DOPE‐conjugated HSA for hydrophobic CUR encapsulation	Enhanced cellular uptake via albumin‐mediated endocytosis Improved ROS generation and mitochondrial depolarization Superior in vivo tumor growth inhibition versus free CUR Good hemocompatibility and colloidal stability	[241]
	Folate‐decorated BSA conjugate	Desolvation‐crosslinking encapsulation of CDF in FA–BSA NPs	Enhanced folate receptor‐mediated cellular uptake Improved synergistic cytotoxicity with PTX in ovarian/cervical cancer cells Sustained pH‐responsive drug release Reduced nonspecific uptake in low‐FR cells	[242]
	PLGA NPs	Preparation of curcumin‐loaded PLGA NPs	Prolonged the retention time of curcumin in the cerebral cortex and hippocampus by 96 and 83%, respectively Protected neurons	[243]
	PLGA–SA–GEL NPs	Curcumin is encapsulated in PLGA NPs and integrated into a 3D‐printed SA‐GEL scaffold structure.	Rapid sublingual dissolution to avoid the hepatic first‐pass effect and the low bioavailability of orally administered curcumin Reduced the dosing frequency of curcumin and increased its stability Noncytotoxic	[246]
	PLGA–PEG–B6 NPs	Conjugating PLGA–PEG with B6 peptides for the encapsulation of curcumin	Increased the cellular uptake of curcumin with good blood compatibility Enhanced spatial learning and memory abilities	[247]
	PLGA–Tet‐1 NPs	PLGA‐coated curcumin NPs conjugated with Tet‐1 peptide	Increased neuronal uptake Exhibited antioxidant properties and noncytotoxicity	[249]
	PLGA–GSH NPs	Encapsulation of curcumin in glutathione‐functionalized PLGA NPs	Avoided lysosomal degradation Enhanced the water solubility and stability of curcumin	[245]
	PLGA copolymer	Single emulsion‐solvent evaporation encapsulation of curcumin in PLGA NPs	Enhanced antiproliferative activity in gastric cancer cells (AGS) versus free curcumin Lowered IC_50_ (18.78 µM at 72 h) compared with native curcumin Induced stronger sub‐G0 phase cell cycle arrest and apoptosis Increased bioavailability and therapeutic efficacy via nanoparticulate protection	[252]
	PEI‐functionalized PLGA copolymer	O/W emulsion‐solvent diffusion encapsulation of CUR with PEI‐mediated siRNA complexation	Enabled simultaneous codelivery of curcumin and Bcl‐2 siRNA Enhanced cellular uptake via cationic PEI surface modification Exhibited synergistic cytotoxicity versus single agents Reduced cell viability	[253]
	PLGA copolymer coencapsulating curcumin and bioperine	Single emulsion‐solvent evaporation for dual‐drug PLGA nanoformulation	Enhanced curcumin bioavailability via bioperine‐mediated bioenhancement Inhibited ox‐LDL‐induced foam cell formation in THP‐1 macrophages Reduced relative cholesterol content in foam cells Maintained >80% cell viability with good biocompatibility	[254]
	Chitosan‐modified PLGA copolymer coencapsulating AT–CU conjugate	Single emulsion‐solvent evaporation of AT–CU conjugate in CS–PLGA matrix	Enhanced aqueous solubility via ester‐linked AT–CU conjugate Suppressed PLGA burst release through chitosan surface modification Achieved high encapsulation efficiency with positive zeta potential	[255]
	PLGA copolymer codelivering curcumin and microbiota‐derived SCFAs	Double emulsion‐solvent evaporation for Cur encapsulation with postadsorption of SCFAs	Sustained curcumin release with high biocompatibility Reduced intracellular ROS to 65.1% and enhanced wound closure Dose‐dependently alleviated RE severity and restored colon length Suppressed NF‐κB signaling and proinflammatory cytokines; upregulated tight junction proteins	[256]
Solid lipid nanoparticles	Cur–SLNs	Encapsulates curcumin in a solid lipid matrix and uses surfactants (Polysorbate 80) and soy lecithin	Significantly improves the oral bioavailability of curcumin Prolongs the in vivo circulation time of the drug Improves the stability of curcumin Achieves sustained drug release	[230]
	Cur–SLNs	Prepared by high‐pressure homogenization and ultrasonic technology	Improves the water solubility and bioavailability of curcumin and reduces the enzymatic degradation rate Good biocompatibility	[231]
	Cur–SLNs	Prepared by solvent evaporation method; modifies SLNs with PEG	Improves the bioavailability and brain delivery efficiency of curcumin Prolong the in vivo circulation time of nanoparticles Enhanced blood–brain barrier penetration ability, and improve the retention time of curcumin in the brain Reduced cytotoxicity	[232]
	Cur–SLNs	Prepared by microemulsification method with Polysorbate 80 and soy lecithin as excipients, encapsulates curcumin	Increased bioavailability Improves brain tissue structure, reduces neuronal degeneration, edema and vascular congestion, with no obvious in vivo toxicity	[257]
	Cur–SLNs	Prepared by emulsification‐evaporation method	Improves the water solubility and dispersibility of curcumin Enhance blood–brain barrier penetration Achieved sustained drug release	[162]
	Cur–SLNs	Cur–SLNs are prepared by melt emulsification–ultrasonication method and optimized by Box–Behnken design; then loaded into poloxamer‐based thermosensitive gel	Suitable for nose‐to‐brain delivery treatment, with good formulation stability Good formulation stability Reduces the direct exposure of curcumin to improve biocompatibility	[258]
	Cur–SLN–MN	Cur–SLNs are prepared by microemulsion method with glyceryl monostearate as lipid and Tween 80 as stabilizer; then embedded into PVA microneedle array	High transdermal permeation efficiency	[259]
	HSPC + DSPE–PEG2000	Thin‐film ultrasonic dispersion encapsulating CU1 in PEGylated lipid matrix	Enhanced cellular uptake and sustained release over 96 h Significantly improved pharmacokinetics with liver safety	[260]
	ConA–Cur–SLNs	Solvent injection encapsulation of curcumin with surface ConA conjugation via EDC/NHS chemistry	Enhanced A549 lung cancer cell inhibition versus nontargeted Cur–SLNs Sustained drug release over 24 h with suitable aerosol performance Lectin‐mediated targeting via carbohydrate recognition on cancer cells	[261]
	C‐SLNs	High‐pressure homogenization encapsulation of curcumin in solid lipid matrix	Enhanced oral bioavailability and dose‐dependent amelioration of CFA‐induced arthritis versus free curcumin Attenuated oxidative–nitrosative stress, TNF‐α, CRP, and CCP–antibody levels Preserved joint architecture and reduced radiological damage comparable to naproxen	[262]
	Cur–SLNs	Emulsification‐low‐temperature solidification encapsulating curcumin in lipid matrix	Superior suppression of LPS‐induced IL‐1β, IL‐6, and TNF‐α versus free curcumin in septic mice Attenuated multiple organ injury via TLR4/2‐NF‐κB pathway inhibition Enhanced anti‐inflammatory efficacy comparable to dexamethasone with gender‐related differences	[263]
	THC–SLNs gel	Microemulsification encapsulation of tetrahydrocurcumin in lipid matrix with hydrogel incorporation	Enhanced topical bioavailability with 17‐fold higher skin permeation versus free THC gel Superior anti‐inflammatory and wound healing activity in excision wound mice model Colorless, nonirritating, and stable formulation with good occlusivity and rheological properties	[264]
Gold nanoparticles	Cur–PFG‐NPs	Modify polypeptides onto gold nanoparticles via gold‐sulfur bonds, and introduce the GGG tripeptide as a spacer	Reduced cytotoxicity Possesses good biocompatibility and nontoxicity	[267]
	Cur–AuNPs	Covalently conjugate curcumin with gold nanoparticles	Reduced cytotoxicity Improves stability	[268]
	Cur–AuNPs	Green synthesis of Cur–AuNPs using Curcuma longa extract as a reducing and stabilizing agent	Increased the ability of curcumin to cross the BBB	[269]
	Cur–AuNPs	Green synthesis using curcumin for simultaneous reduction of Au^3^^+^ and surface functionalization	Superior cardioprotection versus free curcumin against DOX‐induced acute cardiotoxicity Attenuated serum cardiac biomarkers and inhibited cardiomyocyte apoptosis Prevented body and heart weight loss with sustained cardiac accumulation over 14 days	[270]
	Cur–AuNPs	Curcumin‐functionalized green‐synthesized gold nanoparticles	Attenuated AAPH‐induced cardiac lipid peroxidation, ROS, and modulated SOD/CAT/GR in chicken embryo model Enhanced total antioxidant capacity without inducing hepatotoxicity or cytotoxicity in vitro Preserved vascular morphology in CAM with sustained curcumin release	[271]
	Cur–AuNP–hydrogel	Green synthesis using curcumin as reducing and capping agent for AuNP formation with hydrogel encapsulation	Achieved dose‐dependent antioxidant activity with DPPH scavenging peaking at 50 µg/mL and enhanced cytocompatibility with H9c2 cell proliferation Reduced cardiac injury biomarkers CK–MB, LDH, and CPK while decreasing oxidative stress markers MDA and nitrite and increasing GSH in isoproterenol‐induced MI rats Attenuated inflammation by reducing CRP, TNF‐α, and IL‐10 levels with preserved cardiac histology and heart/body weight ratio	[272]
	Cur/Au@ZIF‐8 NPs	One‐pot coembedding of Cur and AuNPs into ZIF‐8 matrix during self‐assembly	High drug loading with pH‐responsive release Superior in vivo antitumor efficacy in orthotopic liver cancer model Modulated fatty acid metabolism with 26 key differential metabolites identified via metabolomics	[273]
	AuNP–ECM–Cur–HA	Chemical crosslinking of AuNPs to ECM with physical mixing of curcumin and HA for injectable scaffold formation	Reduced intracellular reactive oxygen species and mitigated IL‐1β‐induced chondrocyte apoptosis in vitro Maintained low injection force with biocompatibility in fibroblasts and chondrocytes	[274]
	Esb–Lipo–AC	Thin‐film hydration‐sonication encapsulating Ato and Cur with surface Esb peptide conjugation	Synergistically inhibited adhesion molecules and inflammatory factors in dysfunctional ECs Reduced atherosclerotic lesions and plasma lipids via HMG–CoA reductase suppression Alleviated Ato‐induced cytotoxicity through Cur codelivery with active targeting	[285]
	TD‐1 + Cur–Lips	Microfluidic chip encapsulation of Cur in liposomes with physical mixing of TD‐1 peptide	Enhanced skin penetration via stratum corneum lipid disruption and appendageal routes Superior in vivo melanoma inhibition with reduced tumor volume and VEGF levels versus Cur–Lips alone Safe and biocompatible with no body weight loss or systemic toxicity	[286]
Liposome	Compritol 888 ATO and oleic acid	Curcumin is loaded into liposomes	Increased the ability of curcumin to cross the BBB Achieved sustained release of curcumin The blood concentration increased 6.4‐fold compared with free curcumin	[280]
	CL–WGA	Loading curcumin into liposomes CL and WGA	Improved the ability of curcumin to cross the BBB Inhibited cytotoxicity Reduced neuronal apoptosis	[282]
	SLN–NLC	Loading curcumin using solid lipid NPs and nanostructured lipid carriers	Increased curcumin brain uptake, resulting in a more than fourfold increase in curcumin absorption by the brain	[281]
	PLGA–NBs	Encapsulating curcumin in lipid PLGA nanobubbles	Effectively released curcumin, achieving a release rate of 30% within 6 h Increased the solubility of curcumin	[281]
	Lipo–Cur + Lipo–Res	Thin‐film hydration encapsulation of curcumin and resveratrol with liposomal DMPC	Enhanced systemic bioavailability of curcumin when coadministered with resveratrol Significantly reduced prostate adenocarcinoma incidence in PTEN–KO mice via p‐Akt/mTOR/AR downregulation Synergistically inhibited PTEN–CaP8 cell growth and induced apoptosis	[286]
	Cur–PIP–Lipo–Gel	Ethanol injection encapsulation of curcumin and piperine with factorial optimization and hydrogel incorporation	Achieved high entrapment efficiency of 94% for curcumin and 89% for piperine with sustained release over 24 h and enhanced skin deposition of 74.23% Exhibited strong DPPH radical scavenging activity with synergistic antioxidant effects from curcumin and piperine combination Significantly reduced erythema, scratching behavior, ear thickness, and spleen index in DNCB‐induced atopic dermatitis mouse model comparable to dexamethasone treatment	[287]
Nanoemulsions	Cur–NE	Prepare oral turmeric extract (TUR) nanoemulsion by emulsification, optimize the formulation (Tween 80, absolute ethanol, MCT, lecithin, etc.)	Enhance the stability of curcumin and inhibit its alkaline degradation Improve antioxidant capacity Significantly increase oral bioavailability Show good safety with repeated administration	[294]
	Cur–NE	Screen formulations based on pseudoternary phase diagrams (Labrafac Lipophile WL 1349 as oil phase, Solutol HS 15 as surfactant, Transcutol HP as cosurfactant)	Improve water solubility and intestinal permeability Enhanced stability Promote in vitro lipolysis and drug release	[295]
	Cur–SMEDDS	Prepare self‐microemulsifying system with oleic acid as oil phase, Tween 80 as surfactant, and propylene glycol as cosurfactant	Improve water solubility and bioavailability of curcumin, and enhance brain permeability	[296]
	Curc–NE–CT	High‐pressure homogenization encapsulation of curcumin in oil nanodroplets with chitosan coating	Enhanced H9c2 cardiomyoblast viability with superior chitosan‐coated uptake Reduced lipid peroxidation and NO production by ∼40–50% under proinflammatory conditions Suppressed IL‐6, IL‐8, IL‐1β, TNF‐α, and elevated IL‐10 secretion, comparable to lycopene‐rich formulations	[297]
	G‐Cur–NE	Low‐energy emulsification encapsulating curcumin in olive oil nanodroplets with protein coating	Enhanced curcumin stability against autoxidation with sustained protection over weeks via olive oil antioxidants and gelatin shielding Improved cellular uptake and selective anticancer activity against MDA‐MB‐231 breast cancer cells with minimal effect on healthy L929 fibroblasts Maintained cytocompatibility through low surfactant concentration and protein‐mediated membrane transit	[298]
	CU/FU–LN	Emulsification coencapsulating curcumin and 5‐fluorouracil in lipid nanodroplets	Enhanced oral bioavailability with 8.85‐fold and 8.59‐fold AUC increase for curcumin and 5‐fluorouracil versus free drugs Achieved synergistic anticancer efficacy in HepG2 cells with CI = 0.110 at 2:1 molar ratio and 49.29% tumor inhibition in vivo Reduced systemic toxicity with minimal organ lesions and maintained body weight versus free drug treatments	[299]
	Cur/EMO NE@SA	High‐energy emulsification encapsulating CUR and EMO in MCT nanodroplets with pH‐responsive SA hydrogel coating	Achieved pH‐responsive colonic drug release with minimal gastric leakage and controlled release in simulated colonic fluid Synergistically reduced proinflammatory cytokines TNF‐α and IL‐6 while increasing IL‐10 and scavenging ROS in macrophages Restored intestinal mucosal barrier integrity by upregulating ZO‐1 and occludin expression in DSS‐induced colitis mice	[300]
Polymer micelles	NC	Prepare PLA–PEG–PLA triblock copolymer nanomicelles by thin film hydration method, load curcumin	Significantly increase oral bioavailability	[302]
	Cur–DP	Use deoxycholic acid‐conjugated polyethyleneimine (DP) as carrier, prepare micelles by oil‐in‐water emulsification/solvent evaporation method, and coload curcumin and miR‐21 antisense oligonucleotide (miR21ASO)	Improve water solubility and intracellular delivery efficiency of curcumin Enhance the stability and delivery effect of miR21ASO, and synergistically downregulate miR‐21 expression	[303]
	Cur–MI	Prepare curcumin micelles with Tween 80 as carrier, simulate the structure of physiological micelles in the intestine	Significantly improve bioavailability Protect mitochondrial function Enhance cell vitality	[304]
	DSPE–PEG_2000_	Loading curcumin into micelles	Enhanced biocompatibility Reduced cytotoxicity	[305]
	ACP micelles	Covalent incorporation of curcumin into polymer backbone via acrylation and self‐assembly into micelles	Achieved acid‐triggered curcumin release with 28% drug loading and sustained release under acidic conditions Suppressed TNF‐α and IL‐1β expression and reduced ROS levels in LPS‐stimulated macrophages and MIA‐induced osteoarthritis model Preserved cartilage structural integrity with enhanced extracellular matrix maintenance versus free curcumin	[306]
	Cur micelles	One‐step solid dispersion encapsulating curcumin into amphiphilic copolymer micelles	Achieved high drug loading of 14.07% and encapsulation efficiency of 96.08% with small particle size of 27.6 nm for intravenous administration Enhanced cellular uptake and apoptosis induction in CT26 colon cancer cells versus free curcumin Suppressed subcutaneous CT26 tumor growth via inhibiting proliferation and angiogenesis while inducing apoptosis with minimal systemic toxicity	[307]
Exosomes	Cur–MSC–EXO	Pretreat mesenchymal stem cells with 10 µM/L curcumin, collect the secreted exosomes (CUR–MSC–EXO)	Enhances anti‐inflammatory and neuroprotective potential of exosomes Increases expression of anti‐inflammatory cytokines (IL‐10, TGF‐β) Reduces hippocampal neuronal degeneration	[314]
	Cur–hEnSCs EXOs	Load curcumin into human endometrial stem cell exosomes via incubation	Improves water solubility, stability, and blood–brain barrier penetration of curcumin Inhibits α‐synuclein aggregation	[315]
	C3/TPP–EXO	Construct C3/TPP–EXO carriers to encapsulate curcumin via incubation with synthetic DSPE–PEG2000–C3 peptide and DSPE–PEG2000–TPP	Enhances water solubility and bioavailability of curcumin Improves the ability of curcumin to cross the blood–brain barrier	[316]
	MP@Cur–MExo	Construct MP@Cur–MExo engineered exosomes, which release Cur–MExo and MK–SPION upon cleavage by MMP‐2	Enhances brain targeting and biological stability of curcumin Inhibits Aβ aggregation and intracellular Aβ production	[317]
	REXO–C/ANP/S	Construct a ROS–responsive gene–chem drug nanocomplex core, coat it with RVG‐modified exosomes, and deliver curcumin and siSNCA via membrane fusion	Improves bioavailability of curcumin and siRNA, avoiding lysosomal degradation Synergistically clears α‐synuclein aggregates	[318]
	Cur–EL4–EXO	Coincubation of curcumin with exosomes for hydrophobic insertion into lipid bilayer	Increased curcumin solubility by fivefold and protected against degradation with >80% remaining after 150 min versus 25% for free curcumin Enhanced systemic bioavailability with 5–10‐fold higher plasma curcumin levels versus free drug after intraperitoneal administration Improved anti‐inflammatory efficacy in LPS‐induced septic shock model with reduced IL‐6, TNF‐α, and CD11b + Gr‐1+ cell population and improved survival versus free curcumin	[319]
	Cur–EXOs	Coincubation of curcumin with MSC exosomes for hydrophobic encapsulation in lipid bilayer	Enhanced curcumin stability in PBS and plasma with >85% remaining after 120 min versus rapid degradation of free curcumin Achieved 52% encapsulation efficiency and pH‐responsive release with 85% cumulative release at pH 6.6 over 48 h Suppressed LPS‐stimulated HIG‐82 synovial cell proliferation and induced apoptosis via downregulation of IAP1, IAP2, IL‐6, TNF‐α, MMP1, and PGE2 expression	[320]
	EXOs–PH20@Cur	Electroporation encapsulation of curcumin into PH20‐engineered exosomes	Achieved deep tumor penetration via PH20‐mediated hyaluronan degradation with enhanced ECM barrier disruption and vascular perfusion improvement Normalized cancer‐associated fibroblasts by blocking PRMT5‐strengthened Smad3‐mediated fibrotic gene transcription, reducing α‐SMA, Collagen I, and fibronectin expression Suppressed desmoplastic TNBC tumor growth and metastasis by inhibiting TGF‐β and IL‐6 mediated CAF‐cancer cell crosstalk while promoting CD8+ T cell infiltration	[321]

Abbreviations: ACP, acid‐activatable curcumin polymer; AT–CU, atorvastatin–curcumin conjugate; AuNP–ECM–Cur–HA, gold nanoparticle–extracellular matrix–curcumin–hyaluronic acid; AuNPs: curcumin‐biosynthesized gold nanoparticles; BBB, blood–brain barrier; BSA, bovine serum albumin; C3/TPP–EXO, exosomes modified with DSPE–PEG2000–C3 and DSPE–PEG2000–TPP; CAM, chorioallantoic membrane; CDF, difluorinated curcumin; CHP, casein hydrophobic peptide; CK‐MB, creatine kinase‐MB; CL, cardiolipin; CPK, creatine phosphokinase; CS, chitosan; Curc–Ne–CT, chitosan‐coated nanoemulsion loaded with curcumin; Cur–Lips, curcumin liposomes; DNCB, 2,4‐dinitrochlorobenzene; DOX, doxorubicin; DPPH, 2,2‐diphenyl‐1‐picrylhydrazyl; DSPE–PEG2000, 1,2‐distearoyl‐sn‐glycero‐3‐phosphoethanolamine–N‐poly(ethylene glycol)‐2000; DSPE–PEG2000, distearoylphosphatidylethanolamine–polyethylene glycol 2000; DSS, dexosan sulfate sodium salt; EDC, N‐(3‐dimethylaminopropyl)‐N‐ethylcarbodiimide; EMO, emodin; FA–BSA, folic acid decorated bovine serum albumin; FR, THP‐1 the human monocytic cell line; GEL, gelatin; GSH, glutathione; hEnSCs EXOs, human endometrial stem cell‐derived exosomes; HMG–CoA, 3‐hydroxy‐3‐methylglutaryl–CoA; HP‐β‐CD, hydroxypropyl‐β‐cyclodextrin inclusion; HSPC, hydrogenated soybean phosphatidylcholine; IAP1, inhibitor of apoptosis protein 1; IAP2, inhibitor of apoptosis protein 2; LDH, lactate dehydrogenase; Lipo–Cur, liposomal curcumin; MCT, medium chain fatty acids; MDA‐MB‐231, MDA‐MB‐231 breast cancer cells; MI, myocardial infarction; MSC–EXO, mesenchymal stem cell‐derived exosomes; NBs, nanobubbles; NE, nanoemulsion. SMEDDS, self‐microemulsifying drug delivery system. NC, nanomicelles; DP, polymeric micelles; NHS, N‐hydroxysuccinimide; NLC, nanostructured lipid carriers; NPs, nanoparticles; OVA–CMC, ovalbumin–sodium carboxymethyl cellulose; PEG, polyethylene glycol; PFG‐NP, polypeptide‐functionalized gold nanoparticles; PGE2, prostaglandin E2; PRMT5, protein arginine methyltransferase 5; PLGA, polylactic acid‐hydroxyacetic acid; PRO–POL, protein–polysaccharide; PTEN–KO, PTEN knockout; PTX, paclitaxel; REXO‐C/ANP/S, RVG peptide‐modified immature dendritic cell exosomes encapsulating curcumin/phenylboronic acid‐poly(2‐dimethylaminoethyl acrylate) nanoparticles/siSNCA; SA, sodium alginate; SA, sodium alginate; SCFAs, short‐chain fatty acids; SLG, solid lipid nanoparticle‐thermosensitive in situ gel; SLN, solid lipid nanoparticles; SLN–MN, solid lipid nanoparticle‐dissolving microneedle patch; TUR‐NE, turmeric extract nanoemulsion; VEGF, vascular endothelial growth factor; WGA, wheat germ agglutinin.

## 5 Therapeutic Effects of Curcumin in the Treatment of Multiple diseases

The development of nanoformulations has significantly enhanced the bioavailability and stability of curcumin, enabling both controlled release and targeted delivery. These advancements ensure that curcumin maintains effective systemic and cerebral concentrations, thereby improving therapeutic outcomes. Overall, preclinical in vivo and in vitro studies have confirmed the positive effects of curcumin in ameliorating neurological disorders, cancer, cardiovascular diseases, inflammatory conditions, and autoimmune diseases. A deeper understanding of the mechanisms underlying the effects of curcumin is crucial for the development of treatment strategies and its integration into clinical trials. Table 2 summarizes the therapeutic effects observed in both in vivo and in vitro studies.

**TABLE 2:** Preclinical application of curcumin in the treatment of various diseases.

Diseases	Study type	Models	Administration method	Pathway and mechanism of action	Outcome	References
Alzheimer's disease	In vitro	PC12 cells	Pretreatment 10 µg/mL for 1 h	Oxidative stress	SOD↑, CAT↓, DNA damage↓, intracellular calcium levels↓, tau phosphorylation↓	[326]
	In vitro	Primary microglia	Pretreatment 10 µM for 4 h	ERK1/2, p38 MAPK signaling pathway	IL‐1β↓, IL‐6↓, TNF‐α↓, ERK1/2↓, JNK↓, and P38 phosphorylation↓	[328]
	In vitro	BV‐2 cells	Pretreatment 20 µM for 4 h	TLR4/NF‐κB signaling pathway/antioxidant	IL‐6↓, TNF‐α↓	[328]
	In vitro	SH‐SY5Y cells	Treatment 5 µmol/L for 0, 12, 24, and 48h	Wnt/β‐catenin signaling pathway/antioxidant	GSK‐3β↓, activate the Wnt/β‐catenin signaling pathway	[225]
	In vivo	Male C57BL/6J mice	Gavage treatment at 150 mg/kg for 10 days	AMPK signaling pathway/oxidative stress	Improve learning and memory abilities in mice, increase the number of damaged neurons, Bax↓, BCL‐2↑, IL‐1β↓, IL‐6↓, TNF‐α↓, MDA↓, SOD↑	[330]
	In vivo	Male adult Wistar albino rats	Intragastric administration of 100 mg/kg for 60 days	Oxidative/apoptosis	AChE↑, MDA↓, SOD↑, catalase activities↑ INF‐γ↓, IL‐4↑, neurodegeneration in the hippocampus↓	[331]
	In vivo	Ten‐month‐old male and female APPSw Tg+ and Tg− mice	Oral administration of 160 and 5000 ppm for 6 months	NF‐κB signaling pathway/antioxidant	IL‐1β↓, GFAP↓, oxidized proteins↓, reduced the levels of Aβ and plaque burden in the brain	[332]
	In vivo	Male Sprague–Dawley rats	Intraperitoneal injection of 300 mg/kg for 7 days	Inhibition of inflammation	Improve learning ability and neuronal degeneration in Hippocampi, GFAP↓	[333]
	In vivo	Male Wistar rats	Oral administration of 400 mg/kg for 4 weeks	Nrf2 signaling pathway Wnt/β‐catenin signaling pathway/BDNF/TrkB/CREB signaling pathway	Nrf2↑, Aβ↓, pTau↓	[334]
Depression	In vitro	PC12 cells	Pretreatment with 2 × 10^−4 ^M for 2 h	ERK1/2 signaling pathway/specifically inhibit phosphorylation	Alleviate cytotoxicity, ERK1/2 phosphorylation↓	[345]
	In vitro	Primary hippocampal neurons cells	Pretreatment 0.31, 0.62, 1.25 and 2.5 mM for 1 h	CaMKII/NMDA‐R_2_B signaling pathway/specifically inhibit phosphorylation	CaMKII phosphorylation↓, NMDA‐R2B mRNA↓	[346]
	In vivo	Male albino Wistar rats	Oral administration of 200 mg/kg for 7 days	BDNF signaling pathway	Improve depressive behavior and memory performance, CORT↓, BDNF↑, 5‐HT↑, DA↑, acetylcholine↑	[247]
	In vivo	Male Wistar rats	Oral administration of 30 mg/kg for 9 weeks	AC‐cAMP signaling pathway	sucrose intake↑, CORT↓, adenylate cyclase activity↑, cAMP/CREB↑, 5‐HT↑	[349]
	In vivo	Adult Kun‐Ming mice	Intraperitoneal injection of 50 mg/kg for 7 days	NF‐κB signaling pathway	IL‐1β↓, TNF‐α↓, iNOS↓, COX‐2↓, NF‐κB↓	[350]
	In vivo	Male Wistar rats	Gavage treatment of 10 mg/kg for 5 weeks	BDNF/ERK signaling pathway	BDNF↑, ERK↑	[351]
	In vivo	Male Sprague–Dawley rats	Oral administration of 1.25, 2.5, 5, 10 mg/kg for 14 days	Monoaminergic neurotransmitter pathways	5‐HT↑, DA↑, NA↑	[352]
	In vivo	Male Laca mice	Oral administration of 200 mg/kg for 28 days	Oxidative stress	MDA↓, nitrite concentration↓, GSH↑ SOD↑, mitochondrial enzyme complex↑ Acetlycholinestrase↓, CORT↓	[356]
	In vivo	Male Wistar rats	Intraperitoneal injection of 40 mg/kg for 6 weeks	MAPK/ERK/BDNF signaling pathway	Amygdala neuronal synaptic number density, surface density, PSD‐95↑, BDNF↑	[35]
Ischemic stroke	In vitro	Primary microglia	Treatment 12.5 µM for 12 h	NF‐κB/NLRP3 signaling pathway	NF‐κB↓, NLRP3↓, GSDMD↓, Caspase‐1↓, IL‐1β↓, IL‐18↓	[63]
	In vitro	N2a cells	Treatment 5, 15, 25, and 35 µmol/L for 24 h	Regulate the mitochondrial apoptotic pathway	BCL‐2↑, Caspase‐3↓, Bax↓	[47]
	In vitro	SH‐SY5Y cells	Treatment 100 µg/mL for 48 h	Regulate oxidative stress‐related pathways and neuroinflammation regulatory pathways	ROS↓, cytotoxicity↓	[364]
	In vitro	BV‐2 cells	Treatment 12.5, 25 µmmol/L for 48 h	Regulatory pathways of microglial polarization	TNF‐α↓, IL‐1β↓, IL‐6↓	[365]
	In vitro	BV‐2 and TH22 cells	Oral administration of 25 and 50 mg/kg for 14 days	miRNA‐423‐5p/NOD2/NF‐κB/MAPK signaling pathway	iNOS↓, CD206↑, Arg‐1↑, NF‐κB/MAPK↓	[366]
	In vitro	BMECs	Pretreatment 20 µM for 2 h	GMFB signaling pathway	LDH↑, ROS↑, Bax↑, Bcl2↓, GMFB↑	[367]
	In vivo	Male C57BL/6 mice	Intraperitoneal injection of 100 mg/kg for 3 days	AKT/GSK‐3β//Nrf2 signaling pathway	IL‐1β↓, TNF‐α↓, IL‐6↓, IL‐4↑, IL‐10↑, TGF‐β1↑, MDA↓, SOD↓, GPX↓, Nrf2↑	[368]
	In vivo	Male Wistar rats	Intraperitoneal injection of 300 mg/kg for 30min	NF‐κB signaling pathway	Cerebral infarction volume↓, brain edema↓, NF‐κB↓ MMP‐9↓, ICAM‐1↓, Caspase‐9↓	[369]
	In vivo	Male Sprague–Dawley rats	Intraperitoneal injection of 0.1 mg/kg for 15 days	Antioxidant	SOD↑, GSH↓, CAT↓, ROS↓	[370]
	In vivo	Male C57BL/6 mice	Intraperitoneal injection of 25 mg/kg for 3 days	DNMT1/TIMP‐2 signaling pathway	Cerebral capillary permeability↓, improve mitochondrial dysfunction	[371]
	In vivo	Male Sprague–Dawley rats	Intraperitoneal injection of 100 mg/kg for 24 h	PKC‐θ–orai1 signaling pathway	ZO‐1↑, occludin↑, PKC↑, p‐IκBα↑, p65↑, Caspase‐3↓, TNF‐α↓	[372]
	In vivo	Male Sprague–Dawley rats	Intraperitoneal injection of 300 mg/kg for 1 h	PI3K–Akt–Nrf2 signaling pathway	Cerebral infarct volume↓, MDA↓, SOD↑, Nrf2↑, PI3K/Akt↑	[373]
Parkinson disease	In vitro	N2a cells	Treatment 1 µM for 16 h	AKT–MTORC1–TFEB signaling pathway	α‐Syn↓, TFEB↑	[378]
	In vitro	SH‐SY5Y cells	Pretreatment with 100, 250, and 500 nM for 24 h	Nrf2–Keap1 signaling pathway	ROS↓, SOD↑, CAT↑, neuroinflammation‐related factors iNOS↓, Keap↓, and IL‐1β↓	[379]
	In vitro	SH‐SY5Y cells	Treatment 1 µmol/L for 48 h	TFEB–autophagy pathway	α‐Syn↓, TFEB↑, LAMP2A↑, LC3‐II↑	[380]
	In vitro	SH‐SY5Y cells	Pretreatment 2 µM for 1 h	Regulate mitochondrial function pathways	Cell viability↑, mitochondrial membrane Potential↑, ATP↑, mitochondria respiratory↑	[381]
	In vitro	PC cells	Pretreatment 0.5, 1, 2, 5, and 10 µM for 6 h	AKT/mTOR signaling pathway	Cytotoxicity↓, LC3‐II↑, Beclin‐1↑, Akt/mTOR↓	[382]
	In vivo	Male C57BL/6 mice	Intraperitoneal injection of 50 mg/kg for 5 days	JNK signaling pathway/mitochondrial apoptotic pathway	JNK phosphorylation↓, Bcl‐2↑, Bax↓	[383]
	In vivo	Adult male Sprague–Dawley rats	Oral administration of 25 and 50 mg/kg for 14 days	AKT/mTOR signaling pathway	p‐AKT↓, mTOR↓, Beclin‐1↑, LAMP‐1↑, p62↓	[382]
	In vivo	Male C57BL/6 mice	Oral administration of 25, 100, 400 mg/kg for 4 weeks	Regulate the gut microbiota–metabolite axis regulatory pathway	α‐Syn↓, Lactobacillaceae↑, Triocillaceae↑, Ballomyceae↓, Staphylococcaceae↓, tyrosine↑, methionine↑, sarcosine↑, creatine↑, IL‐6↓, IL‐1β↓, TNF‐α↓	[384]
	In vivo	Male C57BL/6 mice	Gavage treatment at 60 mg/kg for 7 weeks	Regulate the gut microbiota‐short‐chain fatty acid (SCFAs) pathway	Improve gastrointestinal dysfunction, SCFA↑	[387]
	In vivo	Male Wistar rats	Oral administration of 200 mg/kg for 2 weeks	α7‐Nicotinic acetylcholine receptor (α7‐nAChR)‐mediated pathway	TH↑, DA↑	[386]
	In vivo	Male C57BL/6 mice	Intraperitoneal injection of 50 mg/kg for 21 days	NLRP3/NF‐κB signaling pathway	Inhibition of microglial activation, NLRP3↓, NF‐κB↓, IL‐1β↓, IL‐18↓, Drp1↓	[387]
	In vivo	Male Sprague–Dawley rats	Intraperitoneal injection of 40 mg/kg for 21 days	NF‐κB signaling pathway/	IL‐6↓, IL‐1β↓, TNF‐α↓, Bax↓, Bcl‐2↑, Caspase‐3↓, Caspase‐9↓, iNOS↓, GSH↓, CAT↓	[388]
Multiple sclerosis	In vitro	CD4^+^T cells	Treatment 5, 10, 20, 40, 60 µM for 24 h	Apoptosis	CD25↓, CD44↓, Bcl‐2↓ Bax↑, Caspase‐3↑	[392]
	In vitro	BV‐2 cells	Pretreatment 10, 30, 50 µM for 2 h	AXL/JAK2/STAT3 signaling pathway	AXL↓, p‐JAK2↓, p‐STAT3↓, iNOS↓, COX2↓, TNF‐α↓, IL‐6↓ TGF‐β↑, IL‐10↑	[393]
	In vitro	Astrocyte	Pretreatment 2.5, 5 µM for 2 h	NF‐κB signaling pathway	IL‐6↓, MMP‐9↓, MCP‐1↓	[394]
	In vitro	T cells	Treatment 0–20 µg/mL for 15 min	IL‐12/JAK2/TYK2–STAT3/STAT4	IL‐12↓, IFN‐γ↓, p‐JAK2↓, p‐TYK2↓, p‐STAT3↓, p‐STAT4↓	[395]
	In vivo	C57BL/6 mice	Oral administration of 100 mg/kg/day for 14 days	Antioxidant effect	TNFα↓, IL‐2↓, IL‐6↓, IFN‐γ, IL‐17A↓, CD3↓, CD4↓ IL‐10↑, MBP↑, SOD↑, CAT↑, GSH↑	[396]
	In vivo	Male C57BL/6 mice	Oral administration of 200 mg/kg/day for 14 days	AMPK/SIRT1 signaling pathway, CREB/BDNF/MBP signaling pathway, JAK2/STAT3 signaling pathway	p‐AMPK↑, SIRT1↑, p‐CREB↑, BDNF↑, MBP↑、Nrf2↑, SOD↑ p‐JAK2↓, p‐STAT3↓, NF‐κB↓, TNF‐α↓	[397]
	In vivo	C57BL/6 mice	Oral administration of 200 mg/kg/day for 21 days	AXL signaling pathway	MBP↑, TGF‐β↑, IL‐10↑ AXL↓, GFAP↓, IL‐6↓, TNF‐α↓, CCL2↓, CXCL10↓	[398]
	In vivo	C57BL/6 mice	Intraperitoneal injection of 100 mg/kg, once every 1 days, for 21 days	AXL/JAK2/STAT3 signaling pathway	TGF‐β↑, IL‐10↑ AXL↓, p‐JAK2↓, p‐STAT3↓, TNF‐α↓, IL‐6↓, iNOS↓, COX2↓, TMEM119↓	[393]
	In vivo	C57BL/6 mice	Intraperitoneal injection of 12.5 mg/kg, once every 1 days, for 29 days	NF‐κB signaling pathway and Nrf2/HO‐1 signaling pathway	IL‐4↑, IL‐10↑, TGF‐β↑, FoxP3↑, Nrf2↑, HO‐1↑, MCP‐1↓, IL‐1↓, IL‐17↓, NF‐κB↓, TNF‐αR↓, iNOS↓	[399]
	In vivo	C57BL/6 mice	Intraperitoneal injection of 1.8 mg/kg, single dose on Days 8, 10, 12, and 14	NF‐κB signaling pathway	NF‐κB↓, ICAM‐1↓, Mac‐1↓	[400]
	In vivo	C57BL/6 mice	Intraperitoneal injection of 100 µg/25 µL, give medication every alternate day, for 25 days	NF‐κB, AP‐1, MAPK, PPARγ signaling pathway	IL‐10↑, PPARγ↑, IFNγ↓, IL‐17↓, IL‐12↓(p35/p40)↓, IL‐23(p19)↓, T‐bet↓	[401]
	In vivo	C57BL/6 mice	Intraperitoneal injection of 50 µg and 100 µg/25 µL, every alternate day, for 25 days	JAK–STAT signaling pathway, NF‐κB signaling pathway	IL‐12↓, IFNγ↓, JAK2↓, TYK2↓, STAT3↓, STAT4↓	[395]
Amyotrophic lateral sclerosis	In vitro	THP‐1 cells	Treatment 100–300 µM for 72 h	Amyloidogenic pathway	SOD1 aggregation↓, cytotoxic aggregates↓	[403]
	In vitro	NSC‐34 cells	Treatment 5 µM for 24 h	Antioxidant and regulation of mitochondrial dysfunction	ROS↓, mitochondrial membrane potential↑	[405]
	In vivo	H46R mutant SOD1 transgenic mice	Oral gavage 10 mg/kg/day for 10 weeks	Antioxidant, Nrf2–ARE pathway	TNF‐α↓, IL‐1β↓, IL‐6↓, Nrf2↑	[406]
Colon cancer	In vitro	CT26 and RKO cell lines	Treatment 23.52–13.62 µM for 72 h	Induction of apoptosis and G_2_ phase cell cycle arrest	Cell viability↓, apoptosis induction, G_2_ phase arrest	[411]
	In vitro	HCT116 cell lines	Treatment 1–10 µM for 1–72 h	NCLX inhibition triggers Ca^2^^+^ overload, ROS, mPTP, and G2/M arrest	Cell viability↓, proliferation↓, apoptosis↑, spheroid volume↓, glycolysis↑ (HIF1α‐dependent)	[412]
	In vitro	Human CRC cell lines (SW620, HCT116) with HPSE overexpression	Treatment 30 µM for 24–48 h	lncXIST/miR‐337‐3p downregulates HPSE, inhibiting IL‐6/STAT5 and EMT	Cell proliferation↓, colony formation↓, migration↓, invasion↓, adhesion↑	[413]
	In vitro	Patient‐derived CRC organoids (TCO1, TCO2 strains)	Treatment 0.6–20 µg/mL for 72 h	ERK/c‐MYC/cyclin‐D1 inhibition suppresses CSCs and induces apoptosis	Cell viability↓ (concentration‐dependent), apoptosis↑, stemness↓	[414]
	In vivo	Swiss nude mice bearing HCT116 CRC xenografts	Intraperitoneal injection of 150 mg/kg, 3 times per week for 5 weeks	NCLX inhibition triggers Ca^2^^+^‐mediated mitochondrial dysfunction and mPTP	Tumor growth↓, tumor volume↓, Ki67‐positive cells↓	[412]
	In vivo	SCID mice bearing patient‐derived CRC organoid xenografts	Oral gavage 20 mg/mouse/day for 21 days	ERK/c‐MYC/cyclin‐D1 blockade suppresses CSC markers and induces apoptosis	Tumor growth↓ (Days 8 and 14), necrotic lesions↑ in tumor tissue	[414]
	In vivo	Nude, BALB/c, C57BL/6 mice bearing CRC xenografts	Oral gavage for 25–500 mg/kg intraperitoneal, intravenous, intratumoral, or feed supplementation 1–28 days	NF‐κB/ERK/Wnt/PI3K blockade induces apoptosis and reduces malignancy	Tumor volume↓, tumor weight↓, body mass unchanged (no toxicity), chemosensitization to 5‐FU, oxaliplatin, and irinotecan	[415]
Breast cancer	In vitro	MDA‐MB‐231 cells	Treatment 75 nM or 48 h for 48 h	TSG hypomethylation and oncogene hypermethylation regulate ECM/cell cycle	Antiproliferative activity, migration inhibition, TSG↑, oncogene↓	[418]
	In vitro	MCF‐7 breast cancer cells	Treatment with 5, 12, and 20 µM curcumin for 96 h	Enhanced uptake inhibits growth via NF‐κB/MAPK/miRNA/rigidity	Growth inhibition↑, chitosan coating improved encapsulation efficiency and enhanced antiproliferative effect	[419]
	In vitro	MCF‐7 breast cancer cells	Treatment 4.8 µg/mL for 48 h	ROS‐driven antiproliferation via pH‐responsive sustained release	Cell viability↓, high apoptosis rate, minimal necrosis (1.13%), enhanced anticancer effect compared with free curcumin	[420]
	In vitro	T47D human breast cancer cells	Irradiation with red light (630 nm, 65.5 mW/cm^2^) for 40 min or blue light (450 nm, 40 mW/cm^2^) for 30 min	Singlet oxygen and hyperthermia drive synergistic anticancer activity	Cell viability significantly↓, effective antitumor effect via PDT/PTT synergy	[421]
	In vivo	BALB/c mice bearing 4T1 breast tumor xenografts	Intravenous and intrathecal 20 mg/kg for once every 2 days	ROS‐mediated TOM70 inhibition and MIC60 import blockade	Tumor growth↓, tumor volume and weight↓, TUNEL+ apoptotic cells↑, cleaved Caspase‐3↑, PCNA↓	[422]
	In vivo	MDA‐MB‐231 TNBC xenograft NSG mice	Intraperitoneal injection 20 mg/kg/day + luteolin 10 mg/kg/day every other day for 5 weeks	IFN‐α/β signaling activation, TGF‐β blockade, c‐Myc/Notch1 downregulation	Tumor volume↓, tumor weight↓, Ki67↓, tumor cell death↑	[423]
	In vivo	BALB/c mice bearing 4T1 triple‐negative breast cancer xenografts	Intraperitoneal injection 5 or 10 mg/kg for 3 weeks	SLC7A11/NF‐κB/TGF‐β signaling pathway	Tumor volume↓, tumor weight↓, Ki‐67↓, lung metastasis nodules↓, E‐cadherin↑, N‐cadherin↓, Vimentin↓, BAX↑, BCL‐2↓, CD11c↑, CD69↑	[424]
Skin cancer	In vitro	A375, C32 (melanotic melanoma)	Treatment 5–200 µM for 4 h (for PDT) or 24/48 h (for cytotoxicity)	Apoptosis, Caspase‐3, DNA damage, and actin cytoskeleton	Cell viability decreased, PDT enhanced curcumin cytotoxicity, increased apoptosis/necrosis, decreased proliferation and migration	[427]
	In vitro	A431, A388 (cutaneous squamous cell carcinoma)	Treatment 2.5–80 µM for 24 h	Skp2, p21, MAPK, AKT, and ROS‐mediated apoptosis	Cell viability↓, colony formation↓, spheroid formation↓, apoptosis↑	[428]
	In vitro	HuT‐78, MJ (cutaneous T‐cell lymphoma)	Treatment 80 µM for 48–72 h	TWIST1, Akt, NF‐κB, apoptosis, and glutathione pathways	TWIST1↓, p‐Akt (Ser473/Thr308)↓, NF‐κB p65 activity↓, Caspase‐3↑, cell proliferation↓, clonogenicity↓, spheroid formation↓, apoptosis↑	[429]
	In vivo	C57BL/6 mice bearing B16‐F10 melanoma cells	Intraperitoneal 20 mg/kg/day for 15 days	Induction of apoptosis, oxidative stress	Tumor growth inhibition, reduced tumor weight	[430]
	In vivo	C57BL/6 mice bearing B16‐F10 melanoma	Posttreatment 5–50 µmol/L for 30 min	ROS‐mediated photodynamic therapy with metal‐enhanced singlet oxygen generation (ME^1^O_2_)	Tumor growth inhibition, prolonged survival (median 12 vs. 9 days), no systemic toxicity, increased skin retention of CUR and AgNPs	[431]
	In vivo	Swiss albino mice (DMBA/croton oil‐induced skin papilloma)	Curcumin‐loaded TPGS‐PLGA nanopatterned films 6.25 µM for 2 weeks	Inhibits SCC progression, induces apoptosis, reduces epidermal hyperplasia	Reduced tumor progression, well‐differentiated papilloma, mild epidermal hyperplasia, minimal dermal infiltrates	[432]
Rheumatoid arthritis	In vitro	RAW264.7 macrophages	Treatment 200 µg/mL for 48 h	NF‐κB/TLR4 inhibition	TNF‐α↓, IL‐1β↓, IL‐6↓, IL‐10↑	[436]
	In vitro	LPS‐activated RAW264.7 macrophages	Treatment 50 µM for 16–48 h	Folate receptor‐mediated endocytosis and ROS‐triggered PEG detachment	TNF‐α↓, IL‐6↓, IL‐1β↓, IL‐8↓, osteoclast differentiation↓, apoptosis rate↓	[437]
	In vitro	Rat primary articular chondrocytes	Pretreatment with 10 µM for 1 h	Activation of autophagy; inhibition of NF‐κB signaling pathway	LC3‐II↑, Beclin‐1↑, p‐p65↓, cleaved‐Caspase‐3↓, cleaved‐Caspase‐9↓, apoptosis rate↓	[438]
	In vitro	LPS‐stimulated HIG‐82 synovial cells	Treatment 20 µM for 48 h	Enhanced cellular uptake and stability; inhibition of antiapoptotic and inflammatory signaling	IAP1↓, IAP2↓, IL‐6↓, TNF‐α↓, MMP1↓, PGE2↓	[320]
	In vivo	Collagen‐induced arthritis (CIA) in male DBA/1J mice	Oral gavage 100 mg/kg for 29 days	Suppression of CHDPs via NF‐κB and proinflammatory cytokine inhibition	Joint CRAMP↓, S100A8↓, S100A9↓; serum TNF‐α↓, IFN‐γ↓, MCP‐1↓	[439]
	In vivo	Collagen‐induced arthritis (CIA) in SD rats	Intra‐articular injection of 15 mg/mL curcumin + blue‐violet light (300 J/cm^2^) daily for 28 days	Downregulation of Nrf2/xCT/GPX4 ferroptosis pathway	Joint swelling↓, synovitis↓, collagen deposition↓, Nrf2↓, xCT↓, GPX4↓	[440]
	In vivo	Collagen‐induced arthritis (CIA) in SD rats	Intravenous injection 4 mg/kg curcumin for 3 days	ROS‐triggered PEG detachment and folate receptor‐mediated active targeting	Joint swelling↓, arthritis index score↓, serum IL‐1β↓, TNF‐α↓, IL‐6↓, IL‐8↓, cartilage erosion↓, circulation time↑	[437]
Systemic lupus erythematosus	In vitro	Conditionally immortalized mouse podocytes	Stimulation with anti‐dsDNA^+^ serum (5%) + curcumin cotreatment for 24 h	Inhibition of NLRP3 inflammasome/Caspase‐1/IL‐1β axis	NLRP3↓, Caspase‐1p20↓, IL‐1β↓	[444]
	In vitro	CD4+ T cells from SLE patients	Treatment with 0.1–1 µg/mL for 72 h	Modulation of Th17/Treg balance via STAT3/mTOR pathways	Th17%↓, IL‐17A↓, Treg%↑, TGF‐β1↑	[445]
	In vivo	Female MRL/lpr mice and R848‐induced C57BL/6J mice	Intraperitoneal injection of 50 mg/kg curcumin once daily for 4–6 weeks	Induction of DNT cell apoptosis via p53/Caspase‐3 pathway	DNT cells↓, Tregs↑ (MRL/lpr), Th1↓ (R848), anti‐dsDNA↓, TNF‐α↓, proteinuria↓, renal IgG/C3 deposition↓	[446]
	In vivo	Female NZBWF1 mice	Oral gavage 500 mg/kg/day for 14 days	Suppression of immune activity and anti‐inflammatory effects	Body weight↑, spleen weight↓, glomerulosclerosis↓, BUN↓	[447]
Psoriasis	In vitro	THP‐1 macrophages and NCTC‐2544 keratinocytes	Treatment with 5 µg/mL CU–LNA–SLNs ± 50 µM IMQ for 6–24 h	Suppression of NF‐κB‐mediated inflammation and ferroptosis	In macrophages: IL‐23↓, IL‐6↓, IL‐8↓, CCL‐20↓; In keratinocytes: proliferation↓, death↓, TFRC↓, MDA↓	[451]
	In vitro	HaCaT keratinocytes and BMDC macrophages	Treatment with 5–20 µg/mL Cur–NPs for 24 h (cytotoxicity) or 30–60 min (uptake)	Suppression of IL‐23/IL‐17 inflammatory axis via NF‐κB inhibition	HaCaT proliferation↓ (IC_50_ = 5.26 µg/mL), cellular uptake↑ (vs. liposomes/nanocrystals), BMDC uptake↑	[452]
	In vitro	M5‐stimulated HaCaT keratinocytes	Treatment with 10 µM for 24–72 h	Inhibition of PI3K/Akt signaling pathway	Cell proliferation↓, KRT6↓, KRT16↓, IL‐1β↓, IL‐6↓, IL‐8↓, IL‐23A↓, TNF‐α↓, MCP‐1↓, p‐Akt↓	[453]
	In vivo	IMQ‐induced psoriasis in male BALB/c mice	Cur–TPP@Mil MN patch applied on Days 3, 5, 7, 9 post‐IMQ induction	Mitochondrial targeting and autophagy activation	Epidermal hyperplasia↓ (70%), Ki67+ cells↓, IL‐17A↓ (60%), TNF‐α↓ (40%), spleen index↓	[454]
	In vivo	IMQ‐induced psoriasis in BALB/c mice	Topical application of CUR–Ph–SHS for 5 days	TLR4 inhibition and DC‐mediated Treg proliferation	Epidermal thickness↓, mast cell infiltration↓, IL‐6↓, IL‐17↓, TNF‐α↓, IL‐1β↓, IL‐23↓, CD80/CD86/MHC‐II on DCs↓, Treg%↑, Ki67+ Treg↑	[455]
	In vivo	IMQ‐induced psoriasis in BALB/c mice	Topical application of CUR–SLN nanogel for 5 days	Suppression of Th17/IL‐23 inflammatory axis via NF‐κB inhibition	PASI score↓, epidermal thickness↓, IFN‐α↓, IL‐23↓, IL‐17↓, TNF‐α↓, spleen/body weight ratio↓	[456]
Osteoarthritis	In vitro	IL‐1β‐stimulated rat primary chondrocytes	Treatment 10 ng/mL IL‐1β for 48 h	SIRT5‐mediated ACSL4 desuccinylation inhibits ferroptosis	Cell viability↑, ferroptosis↓, SIRT5↑, ACSL4 succinylation↓, protein lysine succinylation↓	[460]
	In vitro	SW‐1353 chondrosarcoma cells	Treatment with 15 ppm for 2–24 h	CB2 receptor agonism; inhibition of NF‐κB/ERK pathways	ROS↓, SOD↑, CAT↑, ROS↓, IL‐6↓, COX‐2↓, NFKB1↓, MMP1↓, MMP13↓, HMOX‐1↑, CBR2↑, DAGL‐α↓, MAGL↓	[461]
	In vitro	IL‐1β‐stimulated human chondrocytes	Treatment with curcumin (dose not specified)	Inhibition of TRPM2/NLRP3 signaling axis‐mediated pyroptosis	Cell viability↑, ROS↓, IL‐1β↓, IL‐6↓, TNF‐α↓, IL‐10↓, NLRP3↓, GSDMD↓, procaspase‐1↓	[462]
	In vivo	MIA‐induced OA in male SD rats	Intra‐articular injection of 30 mg/mL once weekly for 4 weeks	NF‐κB inhibition and suppression of macrophage recruitment	Joint edema↓, joint space narrowing↓, cartilage fibrillation↓, hypocellularity↓, proteoglycan restoration↑, synovial inflammation↓, MDA↓, macrophage migration↓	[463]
	In vivo	MIA‐induced OA in male Wistar rats	Oral gavage 100 + 110 mg/kg Omega‐3 daily for 21 days	Mitochondrial protection via COX IV/TOMM20 upregulation	Pain↓ (PWT↑, PWL↑, weight bearing↑), cartilage destruction↓, OARSI score↓, Mankin score↓, MMP‐1/3/13↓, MCP‐1↓, COX IV↑, TOMM20↑	[464]
	In vivo	DMM‐induced OA in male C57BL/6 mice	Nutritional intervention with C3GC for 8 weeks	Chondroprotection and anti‐inflammation via subchondral bone preservation	Subchondral microarchitecture integrity↑, motor function↑, OARSI score↓ (medial tibial plateau), cartilage destruction↓	[465]
Heart failure	In vitro	Phenylephrine‐induced cardiomyocyte hypertrophy	pretreated with 1 and 3 µM for 2 h	Disrupts CDK9/CyclinT1; indirectly inhibits p300‑HAT and RNA pol II	Phenylephrine‐induced cardiomyocyte hypertrophy↓	[469]
	In vitro	Liposomal formulation	In vitro release study in simulated gastric fluid and simulated intestinal fluid; particle size ∼170 nm; encapsulation efficiency up to 97%	Sustained and delayed drug release; PVP coating improves thermal stability; trehalose as cryoprotectant	Particle size ∼170 nm, encapsulation efficiency up to 97%; sustained release profile	[460]
	In vitro	LPS‐stimulated HUVECs	Pretreated with 4 µM curcumin or 4 µM for 24 h	Inhibits inflammation and exosome secretion; regulates miR 142 3p, miR 126 3p, miR 143 3p	Protects vascular smooth muscle cell function	[471]
	In vivo	HFpEF mouse model (high‐fat diet + l‐NAME)	Administered orally at a dose of 20 mg/kg once daily for 4 weeks	Competitively inhibits TGF‐β/TGFBR1; blocks SMAD2/3	Myocardial fibrosis↓, cardiac function↑	[472]
	In vivo	Doxorubicin‐induced cardiotoxicity rat	Oral gavage 100 mg/kg once daily for 14 days	Apelin↑, NF‐κB mRNA↓	Ameliorates QT prolongation, troponin,↓ oxidative stress↓	[473]
	In vivo	STZ‐induced diabetic cardiomyopathy mouse	Oral gavage 5 and 10 mg/kg once every 2 days for 8 weeks	Inhibits OGT → SP1 O‐GlcNAcylation↓ → stabilizes SERCA2a	Alleviates cardiac hypertrophy and fibrosis; effect lost in SERCA2a/OGT‐silenced cells	[474]
	In vivo	Postinfarction mouse model	Oral gavage 120 mg/kg/day for 4 weeks	Nrf2‐SIRT3 signaling → ROS↓, preserves mitochondria	Infarct size↓, fibrosis, hypertrophy, apoptosis	[475]
	In vivo	Post‐MI rat model	Oral gavage 0.2 and 0.5 mg/kg once daily for 6 weeks	Higher bioavailability than Theracurmin	Achieves comparable improvement at lower dose	[476]
	In vivo	Dahl salt‐sensitive rat	Oral gavage 50 mg/kg/day for 6 weeks	Inhibits p300 HAT, GATA4 acetylation↓	LVH without affecting BP or systolic function↓	[477]
	In vivo	Dahl salt‐sensitive rats	Oral gavage 50 mg/kg/d for 6 weeks	Inhibits p300 HAT activity → GATA4 acetylation↓	Posterior wall thickness↓, LV mass index, myocardial cell diameter, perivascular fibrosis, hypertrophy–response gene transcription; no effect on BP or systolic function	[478]
	In vitro	Endothelial cells and macrophages	Pretreated with 10 µM for 2 h	Scavenges ROS, monocyte adhesion↓, inflammatory factors↓	Inhibits foam cell formation	[254]
	In vivo	ApoE−/− mice	Oral gavage at doses of 10, 20, and 40 mg/kg once daily for 4 weeks	Inhibits PDK1/Akt/mTOR → promotes autophagy	VSMC lipid accumulation, inflammation↓	[482]
	In vivo	ApoE−/− mice	Oral gavage at doses of 10, 20, and 40 mg/kg once daily for 28 days	Regulates M1/M2 polarization and lipid metabolism	Improves aortic plaques; superior to simvastatin	[488]
	In vivo	ApoE−/− mice	Intraperitoneal injection at low and high doses of 2.5 and 5 mg/kg for each compound twice weekly for 12 weeks	IL‐17RA‐TAK1‐NF‐κB pathway; SSNB targets TLR4/CD36, curcumin inhibits NF‐κB p65	Aortic lesions and inflammation↓	[483]
	In vivo	ApoE−/− mice	Oral gavage at a dose of 100 mg/kg once daily for 2 months	Restores gut microbiota, TMAO↓, inhibits M1 polarization	Reverses cadmium‐induced plaque progression	[484]
	In vivo	Mouse model of atherosclerosis + ulcerative colitis	Oral gavage at low and high doses of 50 and 100 mg/kg once daily for 4 weeks	Inhibits TLR4/PAD4; METs↓, TNF‐α, IL‐6, IL‐1β, MMP9	Necrotic core↓, collagen↑, stabilizes vulnerable plaques	[486]
Atrial fibrillation	In vitro	Porcine primary hepatocytes	Treated with 25 µM for 24 h	Warfarin‐induced CYP3A↓; inhibits CYP1A2 and CYP3A activity	Food–drug interaction	[488]
	In vivo	Male C57BL/6 mice	Administered at a dose of 100 mg/kg once daily via food feeding from 12 to 18 months	Mature‐mt_tRNA‐Val‐TAC_CCA_end; ROS↓, inflammation, senescence	Ameliorates age‐related atrial fibrosis, AF susceptibility↓	[489]
	In vivo	HL‐1 atrial cardiomyocytes	Pretreated with 50 µM for 2 h	Modulates TGFβ/Smad via TGFBR1	Myocardial fibrosis↓	[490]
	In vivo	Aged mice and senescent human atrial fibroblasts	Oral gavage 50 or 100 mg/kg/day for 6 months	Inhibits p300, blocks p53/Smad3 complex	Atrial fibroblast senescence and fibrosis↓	[491]
	In vivo	Ach‐CaCl_2_‐induced rat AF model	Oral gavage at a dose of 4 mL/kg once daily for 24 days	Regulates IL‐17 signaling; affects Col1a1, Fasn	AF duration, left atrial fibrosis, inflammatory cytokines↓	[492]
Hypertension	In vitro	Male Wistar rats; in vitro dissolution studies	Oral gavage 40 mg/kg for 10 days	Mixed micelle encapsulation enhances solubility and dissolution; bioavailability↑	Particle size ∼24–30 nm; *t*_1_/_2_↑ 10.89‐fold, AUC_0‐∞_↑ 2.65‐fold versus commercial tablets	[494]
	In vitro	Sprague–Dawley rats	Oral gavage 2 mg/kg once daily for 10 days	Inhibits CYP3A4 →↓ amlodipine metabolism	In vivo: amlodipine Cmax↑, AUC↑; in vitro: microsomal↑, intrinsic clearance↓	[495]
	In vitro	Male Sprague–Dawley rats	Treated topically with VASCEPTOR at a dose of 7.5 mg twice weekly for 6 weeks	Transdermal delivery directly to circulation; overcomes poor oral bioavailability	Curcumin may exert therapeutic effects on MetS and its components via multigene, multipathway, and miRNA regulation	[496]
	In vivo	SHR/Izm rats	Oral gavage doses of 100 or 200 mg/kg body weight once daily for 4 weeks	Inhibits brain ACE and AT1, Ang II↓; mAChR and Ach↑	Improves vascular health	[497]
	In vivo	SHR/Izm rats	Oral gavage doses of 100 or 200 mg/kg once daily for 4 weeks	Inhibits brain ACE and AT1, Ang II↓; mAChR and Ach↑	BP↓, improves scopolamine‐induced cognitive impairment	[499]
	In vivo	SHR rats	Intragastric gavage at doses of 100 or 300 mg/kg once every 2 days for 12 weeks	Reshapes gut microbiota,↑ butyrate, activates GPR43 in PVN	BP↓, neuroinflammation↓, intestinal integrity↑	[500]
	In vivo	l‐NAME‐induced hypertensive rat	Oral gavage 50 or 100 mg/kg/day for 14 days	MMP‐2/9↓, oxidative stress, ED1 expression	BP↓, improves renal function and glomerular structure	[501]
	In vitro	High glucose‐treated H9c2 cells	Treated with 20 µM for 24 h	Activates SIRT1; Pgc1α↑, Tfam, Nrf2; inhibits Drp1 translocation	Improves mitochondrial morphology, oxidative stress↓	[504]
	In vitro	High glucose‐treated cardiomyocytes	Treated with 10 µmol/L for 24 h	Promotes Nrf2 nuclear translocation; HO‐1↑ and Gpx4	Lipid peroxides↓, inhibits ferroptosis	[505]
	In vitro	High glucose‐treated H9c2 cells	Pretreated with 10 µM JM 2 for 1 h	Inhibits NF‐κB activation	Suppresses hypertrophy and fibrosis	[506]
	In vitro	High glucose‐treated H9c2 cells	Pretreated with 10 µM for 36 h	Activates Nrf2/HO‐1	ROS↓, antioxidant enzymes↑, inhibits apoptosis	[507]
	In vivo	Male C57BL/6 mice	Intragastric gavage 100 and 200 mg/kg/day for 7 days	Activates Akt/Bad/Caspase‐3	Cardiomyocyte edema↓ and mitochondrial swelling	[508]
	In vivo	Male Sprague–Dawley rats	Intragastric gavage at a dose of 200 mg/kg/day for 8 months	AKT/Nrf2/ARE pathway; inhibits NLRP3‐mediated pyroptosis	Ameliorates diabetic cardiomyopathy	[192]
	In vivo	Male Wistar rats	Oral gavage at a dose of 100 mg/kg daily for 4 weeks	Regulates Beclin‐1, ATG5, LAMP‐2	HIIT more effective than curcumin	[509]
	In vivo	Male Wistar rats	Intraperitoneal injection at a dose of 100 mg/kg 1 h	↓ TLR1, inhibits NF‐κB phosphorylation	Inflammation↓, cardiac function↑	[510]
	In vivo	Male Swiss albino mice	Oral gavage at doses of 100 and 200 mg/kg/day for 20 days	Activates CB2 cannabinoid receptor	Improves hemodynamics, lipid profile, antioxidant capacity; blocked by AM630	[511]
	In vivo	Male C57BL/6 mice	Intragastric gavage at a dose of 120 mg/kg/day for 5 days	↑ MKP‐1, inhibits JNK/ERK; activates Nrf2; modulates Bcl‐2/Bax/Caspase‐3	Oxidative stress↓, inflammation, apoptosis	[512]
Enteritis	In vitro	H2O2‐induced Caco‐2 cells	Treatment with CURG@IN	Macrophage polarization	iNOS (M1 marker)↓, Arg‐1 (M2 marker)↑	[513]
	In vitro	LPS‐induced Caco‐2 cells	CCM at 20, 40, 80 µM	PANoptosis/NLRP3 signaling	Cell death↓, IL‐18↓, IL‐1β↓	[514]
	In vivo	C57BL/6 mice	Intragastric administration of 1.5 mg/kg, once every 1 days, for 9 days		DAI↓, colon length shortening improved↑	[513]
	In vivo	C57BL/6 mice	Enema + gavage administration of 60 mg/kg, Once every 1 days, for 10 days	Tight junction proteins	Histopathological damage↓, IL‐1β↓, TNF‐α↓, IL‐4↑, IL‐10↑, occludin↑, ZO‐1↑	[522]
	In vivo	Balb/c mice	Intragastric administration of 25 mg/kg, once every 1 days, for 7 days	VEGF‐mediated JAK2/STAT3/angiogenesis	Inflammation↓, abnormal angiogenesis↓, Tregs↑	[517]
Gastritis	In vitro	AGS cells	Curcumin oxidative products	Binding to VacA virulence factor	Vacuolation activity induced by Hp↓	[521]
	In vitro	C57BL/6 mice	Oral administration of 100, 200, 400 mg/kg for 4 weeks	—	Hp growth completely inhibited↓	[519]
	In vitro	CD1 mice	Oral administration of 50, 100, 200 mg/kg for 28 days	—	Hp growth↓, urease activity↓	[520]
	In vivo	BALB/C mice	Oral administration of 500 mg/kg, 3 times/week for 6 weeks	PI3K/AKT signaling/oxidative stress	Gastric mucosal inflammation↓, IL‐1β↓, TNF‐α↓, MDA↓, SOD↑, CAT↑	[522]
	In vivo	C57BL/6 mice	Intragastric administration of 500 mg/kg, 3 times/week for 18 weeks	Regulation of immune response	Prevention of malignant progression from chronic atrophic gastritis to gastric cancer↑	[523]
Dermatitis	In vitro	HaCaT cells	Cur–Bet–IL–Lip treatment	IL‐17A signaling pathway	IL‐17A/IL‐17F/IL‐22↓, Collagen‐I synthesis↑, penetration rate↑	[274]
	In vitro	Human fibroblasts	NLC (curcumin + sesame oil)	—	Biocompatibility↑, 24 h sustained release↑, toxicity↓	[524]
	In vivo	BALB/c mice	Microneedle patches of Cur–TPP@Mil, ∼55 µg/patch, on Days 3, 5, 7, 9	Autophagy activation	Epidermal thickness↓, IL‐17A/TNF‐α/IL‐6↓, keratinocyte apoptosis↑, Ki67 positive cells↓	[454]
	In vivo	BALB/c mice	Topical Cur@TA‐Gel patches, every other day, for 21 days	ROS scavenging/macrophage M2 polarization +1	Radiation damage↓, ROS↓, macrophage M2 polarization↑ +1	[525]
Pneumonia	In vitro	LPS‐induced A549 cells	Treated with 0, 5, 10, 20, 30, 40, 80 µM for 2 h	Rac1/NF‐κB/HPSE pathway	Glycocalyx damage reversed↑, HS↑, HPSE overactivation↓	[526]
	In vitro	LPS‐induced RAW264.7	Curcumin 5–20 µmol/L	TLR4/NF‐κB/mitochondrial potential	LPS‐induced damage↓	[527]
	In vitro	DC	Curcumin treatment	—	DC maturation process blocked↓, CD80/CD86 expression↓	[528]
	In vivo	C57BL/6 mice	IP injection of 100, 200, 300 mg/kg, single dose (2 h before LPS)	—	Pulmonary edema↓, Inflammatory infiltration↓, BALF total cells/neutrophils/protein↓, MPO↓, MDA↓	[526]
	In vivo	KM mice	Inhalation of 50, 100 mg/kg Cur–SLN–FL for 14 days	—	BALF IL‐1β/IL‐6↓, ACE2 expression↑	[529]
	In vivo	C57BL/6 mice	IP injection of 20 mg/kg, daily for 9 days	Wnt/β‐catenin signaling	AHR↓, eosinophil infiltration↓	[530]

Abbreviations: 5‐HT, 5‐hydroxytryptamine; ACE, angiotensin converting enzyme; AchE, acetylcholinesterase; AF, atrial fibrillation; AgNPs, silver nanoparticles; LC3‐II, microtubule‐associated protein 1 light chain 3‐II; AHR, airway hyperresponsiveness; Akt/mTOR, protein kinase B/mechanistic target of rapamycin; ALI, acute lung injury; Ang II, angiotensin II; ApoE, apolipoprotein E; ARDS, acute respiratory distress syndrome; Arg‐1, arginase 1; Arg‐1, arginase 1; ATP, adenosine triphosphate; Aβ, amyloid beta; Bax, bcl‐2‐associated x protein; BCL‐2, b‐cell lymphoma 2; BDNF, brain‐derived neurotrophic factor; BMDC, bone‐marrow‐derived dendritic cell; BP, blood pressure; BUN, blood urea nitrogen; C3GC, Curcumin C3 complex–glucosamine–chondroitin; CaMKII, calmodulin‐dependent protein kinase II; cAMP/CREB, cyclic adenosine monophosphate/cAMP response element‐binding protein; Caspase‐1, cysteinyl aspartate‐specific proteinase 1; Caspase‐3, cysteinyl aspartate‐specific proteinase 3; Caspase‐9, cysteinyl aspartate‐specific proteinase 9; CAT, catalase; CCM, curcumin–chitosan microspheres; CD206, mannose receptor c‐type 1; CDC, water‐soluble curcumin complex; HIF1α, hypoxia‐inducible factor 1 alpha; CDK9, cyclin‐dependent kinase 9; CHDPs, cationic host defense peptides; CLE, curcuma longa extract; CLP, cecal ligation and puncture; COPD, chronic obstructive pulmonary disease; CORT, corticosterone; COX IV, cytochrome c oxidase 4; COX‐2, cyclooxygenase‐2; CRC, colorectal cancer; CSCs, cancer stem cells; Cur–Bet–IL‐Lip, curcumin‐based ionic liquid liposome; Cur–SLN–FL, flower‐shaped lactose‐loaded curcumin solid lipid nanoparticles; CVD, cardiovascular disease; DA, dopamine; DAI, Disease Activity Index; DC, dendritic cells; Drp1, dynamin‐related protein 1; DSS, dextran sodium sulfate; EMT, epithelial interstitial transformation; ERK, extracellular‐regulated protein kinases; ERK1/2, extracellular‐regulated protein kinases; GFAP, glial fibrillary acidic protein; GMFB, glial maturation factor beta; GPR43, G protein coupled receptor 43; GPX, glutathione peroxidase; GSDMD, gasdermin d; GSH, glutathione; GSK‐3β, glycogen synthase kinase 3 beta; HFpEF, heart failure with preserved ejection fraction; Hp, Helicobacter pylori; HPSE, heparanase; HPSE, paranase; HS, heparan sulfate; HUVEC, human umbilical vein endothelial cell; IAP1, inhibitor of apoptosis protein 1; IBD, inflammatory bowel disease; ICAM‐1, intercellular cell adhesion molecule‐1; ICU, intensive care unit; IL‐10, interleukin 10; IL‐18, interleukin 18; IL‐1β, interleukin 1 beta; IL‐4, interleukin 4; IL‐6, interleukin 6; IMQ, imiquimod; inducible nitric oxide synthase; INF‐γ, interferon gamma; iNOS; iNOS, inducible nitric oxide synthase; JNK, c‐Jun N‐terminal kinase; l‐NAME, Nω nitro l‐arginine methyl ester; LAMP2A, lysosome‐associated membrane protein 2A; LC3‐II, LC3‐II, microtubule‐associated protein 1 light chain 3‐II; LDH, lactate dehydrogenase; LPS, lipopolysaccharide; LVH, left ventricular hypertrophy; mAChR, muscarinic acetylcholine receptor; MAPK, mitogen‐activated protein kinase; MDA, malondialdehyde; METs, macrophage extracellular traps; MI, myocardial infarction; MMP, matrix metalloproteinase; MMP‐9, matrix metalloproteinase‐9; NA, noradrenaline; NF‐κB, nuclear factor kappa B; NLC, nanostructured lipid carrier; NLRP3, nod‐like receptor family pyrin domain containing 3; NMDAR, N‐methyl‐d‐aspartic acid receptor; Nrf2, nuclear factor erythroid 2‐related factor 2; OGT, O linked N acetylglucosamine transferase; ox‐LDL, oxidized low density lipoprotein; PASI, Psoriasis Area and Severity Index; PCNA, proliferation‐associated molecule; PDT photodynamic; PI3K/Akt, phosphoinositide 3‐kinase/protein kinase B; p‐IκBα, phosphorylated Inhibitor of nuclear factor kappa B alpha; PKC, protein kinase C; PLGA, poly (lactic co glycolic acid); PSD‐95, postsynaptic density protein 95; pTau, phosphorylated tau; PTT, photothermal; PVN, paraventricular nucleus; PVP, polyvinylpyrrolidone; QoL, quality of life; ROS, reactive oxygen species; SERCA2a, sarco/endoplasmic reticulum Ca^2^^+^ ATPase 2a; Sirt5, Sirtuin 5; Skp2, S‐phase kinase‐associated protein 2; SMAD, mothers against decapentaplegic homolog; SOD, superoxide dismutase; STAT5, signal transducer and activator of transcription 5; STZ, streptozotocin; T helper 17, T helper 17; TFEB, transcription factor EB; TFRC, transferrin receptor; TGF β, transforming growth factor beta; TGFBR1, TGF β receptor 1; TGF‐β, transforming growth factor‐beta; TGF‐β,transforming growth factor‐β; TH, tyrosine hydroxylase; TLR, Toll like receptor; TMAO, trimethylamine N oxide; TNF‐α, tumor necrosis factor‐alpha; Treg, regulatory T cells; TRPM3, transient receptor potential cation channel subfamily M member‐3; TSG, tumor suppressor genes; TWIST1, twist‐related protein 1; UC, ulcerative colitis; VEGF, vascular endothelial growth factor; VSMC, vascular smooth muscle cell; ZO‐1; zonula occludens‐1; α‐Syn, alpha‐synuclein.

### 5.1 Neurological Disorders

#### 5.1.1 Alzheimer's Disease

AD is a progressive neurodegenerative disorder primarily characterized by the impairment of memory and cognitive functions. It typically manifests as short‐term memory loss, cognitive deficits, alterations in behavior and mood, and executive dysfunction. The pathological hallmarks of AD include the formation of Aβ plaques and the accumulation of tau protein, which forms neurofibrillary tangles in the brain [323]. The misfolding of Aβ peptides is closely associated with the pathogenesis of neurological disorders, including AD. This process contributes to elevated calcium influx, heightened oxidative stress, cellular inflammation, and the dysregulated expression of ion channel and receptor genes [324]. Intracellular tau tangles involve the hyperphosphorylation of tau protein, resulting in the destabilization of neurofilament microtubules, the disruption of cellular structure and transport systems, and the impairment of normal neuronal function. Accordingly, the elimination of Aβ to reduce its cerebral deposition, along with the inhibition of tau protein hyperphosphorylation, represents a critical strategy in anti‐AD drug development [325].

##### 5.1.1.1 In Vitro Studies

Curcumin is recognized for its potent antioxidant properties. In vitro studies have demonstrated that curcumin protects neuronal cells against Aβ‐induced neurotoxicity. For instance, the pretreatment of PC12 cells with curcumin has been shown to ameliorate neuronal damage by reducing oxidative stress, attenuating Aβ‐induced DNA damage, lowering intracellular calcium levels, and inhibiting tau protein hyperphosphorylation [326]. Activated microglia may be important mediators of Aβ production and can promote the release of cytokines, which enhances the immune response in the brains of patients with AD [327]. Research has indicated that curcumin can suppress the expression of IL‐6, IL‐1β, and TNF‐α, as well as inhibit the phosphorylation of ERK1/2 and P38 in Aβ‐activated microglia, thereby attenuating the microglia‐mediated inflammatory response in the brain [328]. Moreover, Zhang et al. demonstrated that supplementation with curcumin nanoparticles significantly reduced the expression of the proinflammatory factors TNF‐α (51.89 ± 8.61%) and IL‐6 (38.52 ± 4.97%) in LPS‐treated BV‐2 microglial cells [329]. One study established an AD cell model by transfecting APPswe and BACE1‐mycHis plasmids into SH‐SY5Y cells. Curcumin treatment inhibited GSK‐3β expression in the transfected cells, thereby activating the Wnt/β‐catenin signaling pathway. These findings provide a potential therapeutic strategy for AD [225].

##### 5.1.1.2 In Vivo Studies

Animal studies have demonstrated that, in Aβ‐induced AD model mice, curcumin restores damaged neuronal populations and enhances cognitive function and spatial memory. These effects are associated with the inhibition of cellular inflammatory responses, reduced MDA levels, increased SOD activity, and enhanced AMPK phosphorylation [330]. Research has also shown that curcumin reverses AD‐like alterations induced by aluminum chloride in mice. These changes include reduced AChE activity, attenuation of hippocampal oxidative stress, and the suppression of neuroinflammation, characterized by a decrease in the release of TNF‐α and IL‐6 and a concomitant increase in IL‐4 secretion [331]. Notably, the administration of low‐dose curcumin was particularly effective in decreasing both the Aβ content and the plaque load in the brain [332]. It has also been reported that curcumin ameliorated the spatial memory impairment induced by Aβ1−42​ in AD model rats, achieved through the inhibition of glial fibrillary acidic protein expression and the suppression of astrocyte proliferation in the hippocampal CA1 region [333]. Furthermore, Jearjaroen et al. investigated the effects of oral hexahydrocurcumin (HHC) administration (40 mg/kg for 4 weeks) on cognitive dysfunction induced by two‐vessel occlusion in rats [334]. They found that HHC promotes nuclear factor erythroid 2‐related factor 2 (Nrf2) activation, thereby alleviating oxidative stress, downregulating Aβ production, and reducing the levels of phosphorylated tau protein, thereby protecting neurons from injury and ultimately improving dementia in the animals.

Combined, the above findings suggest that the therapeutic effects of curcumin in AD are mediated via the clearance of Aβ deposits, decreasing tau protein phosphorylation levels, and protecting neurons by suppressing neuroinflammation and oxidative stress.

#### 5.1.2 Depression

Depression is a mental disorder characterized by persistent feelings of sadness, diminished interest in normal activities, and physiological dysfunction. Its symptoms include difficulty concentrating, changes in appetite, and disrupted sleep patterns [335]. In severe cases, depression can lead to self‐denial and cognitive distortions, which can ultimately result in suicidal behavior [336]. Numerous studies have indicated that experiencing depression significantly increases the risk of developing other neurological diseases, including stroke, epilepsy, and AD [337]. The etiology of depression is multifaceted. While the mechanisms underlying this condition remain incompletely understood, it is widely accepted that its development arises from the complex interplay among multiple components, including psychological factors, genetic susceptibility, social stress, and comorbidities, among others [338]. Widely recognized mechanisms underlying depression include the monoamine neurotransmitter hypothesis [339], hypothalamic–pituitary–adrenal axis hyperactivity [340], neuroinflammation [341], oxidative stress [342], and gut–brain axis dysregulation [343]. Research into these mechanisms holds promise for providing effective methods for the treatment of depression.

##### 5.1.2.1 In Vitro Studies

A growing body of evidence suggests that the MAPK signaling pathway, particularly the ERK subfamily, plays a critical role in the pathogenesis of depression. Research indicates that ERK signaling is markedly decreased in the prefrontal cortex and hippocampus of patients with depression [344]. Gao et al. assessed the effect of curcumin using a PC12 cell model of depression and found that curcumin significantly enhanced the viability of PC12 cells that had been exposed to corticosterone. Furthermore, western blot analysis indicated that curcumin effectively and dose‐dependently suppressed corticosterone‐induced ERK1/2 phosphorylation in the treated cells, thereby mitigating corticosterone‐induced cytotoxicity [345]. The *N*‐methyl‐d‐aspartate receptor (NMDAR) is a well‐documented crucial binding partner for CaMKII within the postsynaptic density. The binding of phosphorylated CaMKII to NMDAR leads to the activation of a variety of postsynaptic proteins involved in memory processes [346]. An in vitro study reported that curcumin pretreatment effectively attenuates cortisol‐induced neuronal cell death in hippocampal neurons, downregulates NMDAR expression, and reduces excessive CaMKII phosphorylation, thereby safeguarding neurons from cortisol‐induced neurotoxicity [347].

##### 5.1.2.2 In Vivo Studies

In a rat model of depression induced by chronic restraint stress, curcumin treatment significantly ameliorated depressive‐like behaviors. This therapeutic intervention also led to an improvement in memory function impairment, likely through the regulation of cortisol levels and the positive modulation of BDNF expression in the hippocampus [348]. Similarly, Fan et al. reported that curcumin effectively reversed the reduction in sucrose consumption and the increase in serum corticosterone levels induced by CUMS in rats. Curcumin also enhanced the levels of cAMP in the brain and reversed the decline in serotonin (5‐HT) contents, thereby exerting a protective effect against stress‐induced depressive‐like behaviors in the animals [349]. Another study reported that curcumin effectively alleviated LPS‐induced depressive‐like behaviors in mice by inhibiting microglial activation and suppressing the release of proinflammatory cytokines, including IL‐1β, TNF‐α, iNOS, and COX‐2. Additionally, curcumin attenuated the activation of the NF‐κB signaling pathway in the hippocampus and prefrontal cortex [350]. BDNF plays a key protective role in the pathophysiology of depression by promoting neuronal plasticity and survival, synaptic growth and differentiation, and the production of neurotransmitters [351]. Animal studies have demonstrated that prolonged curcumin treatment reverses the decrease in sucrose preference observed in rats subjected to CUMS. Additionally, curcumin upregulates BDNF expression in the hippocampal region. The ERK signaling pathway is highly responsive to stress and plays an essential role in regulating cognitive and emotional processes [352, 353]. Research has shown that curcumin can inhibit ERK phosphorylation, enhance cognitive function, and improve learning and memory capabilities in rats. These data suggest that curcumin exerts its antidepressant effects by upregulating BDNF expression and modulating the ERK signaling pathway [199]. In a study involving a rat model of depression, curcumin administration significantly reduced immobility time in the forced swimming test. Additionally, curcumin treatment effectively reversed the decreases in 5‐HT, DA, and NA levels in the hippocampus and prefrontal cortex of rats subjected to bilateral olfactory bulbectomy, thereby ameliorating depressive‐like behaviors in the animals [354]. Excessive ROS accumulation in the brain can severely impair neuronal function [355]. Rinwa and Kumar exposed mice to CUMS and reported that supplementation with curcumin effectively prevented oxidative damage by increasing the expression of GSH and CAT and reducing the concentrations of MDA and nitrite [356]. Similarly, Zhang et al. demonstrated that in rats with CUMS‐induced depression, curcumin pretreatment increased synaptic density and the surface density of amygdala neurons, while also restoring PSD‐95 and BDNF levels, resulting in significant antidepressant effects [357].

Collectively, these findings indicate that curcumin elevates monoamine neurotransmitter levels, upregulates the expression of BDNF, and promotes neuronal plasticity and survival. In addition, curcumin inhibits the activation of microglia and the release of proinflammatory cytokines, which may help protect neurons and potentially delay the onset and progression of depression.

#### 5.1.3 Ischemic Stroke

Ischemic stroke is an acute cerebrovascular disorder typically caused by the sudden occlusion of cerebral blood vessels, resulting in the interruption of blood supply to the brain. This leads to ischemic lesions in the affected arterial regions, manifesting as neuronal damage or cerebral edema [358]. The clinical manifestations of ischemic stroke primarily include facial nerve paralysis, language impairment, limb weakness, and sensory disturbances [359]. Considering the constrained regenerative potential of the adult brain and the frequently irreversible nature of neuronal injury, the prompt reperfusion of ischemic regions is essential for ameliorating the neuronal damage elicited by ischemic stroke [360]. Notably, cerebral I/R injury following the restoration of blood flow can initiate a cascade of detrimental reactions, ultimately resulting in cellular injury and inflammatory responses within the affected tissue [361].

##### 5.1.3.1 In Vitro Studies

Damage to white matter is a crucial aspect of the pathophysiological processes of ischemic stroke, potentially leading to long‐term sensorimotor deficits and cognitive impairment [362]. Neuroinflammation plays a significant role in the damage and repair of white matter. It ultimately contributes to secondary brain injury primarily through the recruitment of peripheral immune cells, the activation of glial cells, and the release of proinflammatory cytokines [363]. In a study involving primary mouse microglia, curcumin treatment significantly suppressed the activation of NF‐κB and the NLRP3 inflammasome, thereby diminishing the expression of the pyroptosis‐related proteins GSDMD and Caspase‐1 and the proinflammatory cytokines IL‐18 and IL‐1β [63]. Xie et al. explored the effects of curcumin on ischemic stroke using N2a cells subjected to oxygen–glucose deprivation/reperfusion (OGD/R). Treatment with 25 µmol/L curcumin significantly increased the protein levels of BCL‐2 while reducing the expression of BAX and Caspase‐3, thereby suppressing mitochondrial apoptosis. This inhibitory effect on apoptosis highlights the neuroprotective potential of curcumin in the context of ischemic stroke [47]. Yang et al. reported that curcumin‐loaded gelatin nanoparticles (Cur@GAR NPs) protected SH‐SY5Y cells against hydrogen peroxide‐induced oxidative stress and consequent cell death. Notably, these effects were attributed to the potent antioxidant properties of curcumin [364]. A recent study revealed that curcumin inhibits the LPS‐ and interferon‐gamma (IFN‐γ)‐induced M1 polarization in BV2 microglial cells, thereby reducing the expression of proinflammatory cytokines such as TNF‐α, IL‐1β, and IL‐6 [365]. Meanwhile, Lan et al. established an OGD/R model using BV2 and TH22 cells to simulate I/R injury. Pretreatment with curcumin resulted in the downregulation of iNOS protein levels and the upregulation of the M2 microglial markers CD206 and Arg‐1, leading to a significant decline in the release of proinflammatory factors. These data indicated that curcumin exerts significant anti‐inflammatory effects by promoting the polarization of microglia from an M1 to an M2 phenotype. In addition, curcumin modulates neuroinflammatory responses by inhibiting the NF‐κB/MAPK signaling pathway, thereby alleviating PANoptotic neuronal death [366]. Similarly, curcumin was reported to inhibit apoptosis, reduce ROS generation and LDH levels, and restore the damage induced by OGD/R in brain microvascular endothelial cells in vitro [367].

##### 5.1.3.2 In Vivo Studies

In MCAO model mice, combination treatment with curcumin and human umbilical cord‐derived mesenchymal stem cells (hUC‐MSCs) led to a reduction in the cerebral infarction area and brain edema. This combined therapy afforded neuroprotection by reducing ROS levels and downregulating the expression of oxidative mediators and proinflammatory cytokines, effects that were mediated through the Nrf2 pathway [368]. Another group investigated the effects of curcumin on cerebral I/R injury using a similar MCAO model in rats. Compared with untreated MCAO animals, those administered curcumin presented significant reductions in cerebral infarction volume, neurological dysfunction, and brain edema. Furthermore, curcumin downregulated the expression of inflammation‐related markers (NF‐κB, MMP‐9, and ICAM‐1) as well as that of the apoptosis‐related marker Caspase‐9. This implies that the neuroprotective effects of curcumin against cerebral I/R injury may be associated with its anti‐inflammatory and antiapoptotic properties [369]. It was also shown that curcumin nanoparticles improved motor function and muscle strength in rats following ischemic stroke. These nanoparticles effectively enhanced the activity of antioxidant enzymes, including SOD, GSH, and CAT, thereby reducing oxidative stress and providing neuroprotection [370]. Additionally, in mice with MCAO‐induced stroke, tetrahydrocurcumin (THC) treatment significantly improved functional coordination, while simultaneously reducing cerebral infarction volume, brain edema, and microvascular leakage in the brain parenchyma. Furthermore, this curcumin metabolite restored the levels of homocysteine metabolic enzymes, alleviated mitochondrial dysfunction—including mitochondrial oxidative stress and mitochondrial DNA methylation—and normalized MMP‐9 activity in the brain, thereby protecting the mice against I/R injury [371]. Mo et al. simulated I/R injury using a MCAO/reperfusion (MCAO/R) model. The authors reported that curcumin reversed the MCAO/R‐induced increase in calcium ion concentrations and the disruption of the blood–brain barrier, while also protecting against cerebral I/R injury through PKC signaling [372]. Another study demonstrated that curcumin alleviated MCAO‐induced oxidative stress in rats by activating the AKT/Nrf2 signaling pathway. Moreover, curcumin significantly decreased the cerebral infarction area and minimized ischemic neuronal damage, thus exerting neuroprotective effects [373].

Combined, observations from in vitro, in vivo, and clinical trials suggest that curcumin mitigates neuronal ischemic injury and inhibits the progression of ischemic stroke by promoting microglial polarization, suppressing neuroinflammation, reducing oxidative stress, inhibiting cell apoptosis, and alleviating mitochondrial dysfunction.

#### 5.1.4 Parkinson's Disease

PD is a complex neurodegenerative disorder, pathologically defined by the loss of dopaminergic neurons in the substantia nigra pars compacta of the midbrain, the presence of Lewy bodies in neurons, and the aggregation of cytoplasmic inclusions consisting of insoluble α‐Syn [374]. Patients additionally present with progressive impairment of voluntary motor control, characterized by a range of symptoms such as tremor, bradykinesia, difficulty in walking, autonomic dysfunction, and cognitive decline [375]. Furthermore, the prevalence of PD steadily increases with age [376]. Factors such as oxidative stress, mitochondrial dysfunction, neuroinflammation, and deficiencies in neurotrophic factors all significantly influence the onset and progression of the disease [377].

##### 5.1.4.1 In Vitro Studies

It is increasingly clear that impairment of the autophagy–lysosome pathway is a significant contributor to the pathogenesis of PD. Transcription factor EB (TFEB), the master regulator of this pathway, has emerged as a promising therapeutic target for this condition. The treatment of N2a cells with the curcumin derivative E4 (1 µM) for 24 h resulted in increased TFEB phosphorylation and reduced α‐Syn protein levels, suggesting that this treatment exerted a neuroprotective effect [378]. Pirunkaset et al. [379] established a model of PD in human neuroblastoma (SH‐SY5Y) cells via rotenone treatment and examined the ROS scavenging activity and potential neuroprotective effects of the diacetyldicurcumin–manganese complex (DiAc–Cp–Mn). The addition of this complex significantly diminished intracellular ROS release, boosted the activities of SOD and CAT, and suppressed the expression of the neuroinflammatory factors iNOS, KEAP1, and IL‐1β. Similarly, Wu et al. established a PD cell model by treating SH‐SY5Y cells with MPTP. Curcumin treatment significantly increased both the survival of dopaminergic neurons and the clearance of intracellular α‐Syn relative to the model group. Notably, these neuroprotective effects were mediated through the upregulation of TFEB and the autophagy‐related proteins LAMP2A and LC3‐II [380]. In a separate study, the authors established a cell model of PD in SH‐SY5Y cells via the deletion of PINK1. Cells pretreated with curcumin displayed enhanced viability, improved mitochondrial dysfunction—characterized by increased MMP and mitochondrial respiration—and reduced levels of apoptosis [381]. It has also been reported that curcumin inhibits 6‐OHDA‐induced apoptosis in PC12 cells, suppresses cytotoxicity, upregulates the expression of the autophagy markers LC3‐II and Beclin‐1, and exerts neuroprotective effects via the inhibition of the AKT/mTOR signaling pathway [382].

##### 5.1.4.2 In Vivo Studies

In mouse models of PD, curcumin has demonstrated potential in mitigating the death of dopaminergic neurons and the loss of dopaminergic axons in the striatum. Specifically, research has shown that the intraperitoneal injection of curcumin (50 mg/kg/day) for 5 consecutive days inhibits JNK phosphorylation and modulates the protein expression of BCL‐2 and BAX, resulting in significant neuroprotective effects following the onset of PD [383]. Furthermore, curcumin can diminish α‐Syn accumulation in a 6‐OHDA‐induced rat model of PD as well as inhibit AKT/mTOR signaling pathway‐mediated autophagy. These findings underscore the potential value of curcumin in the treatment of PD [382]. In MPTP‐induced PD model mice, curcumin effectively ameliorated motor deficits, suppressed glial cell activation, and reduced α‐Syn aggregation. Meanwhile, gut microbiota analysis revealed that curcumin significantly increased the abundance of Lactobacillaceae and Turicibacteraceae while decreasing that of Bifidobacteriaceae and Staphylococcaceae. Additionally, curcumin treatment elevated the serum levels of tyrosine, methionine, sarcosine, and creatine [384]. Consistent with these findings, in mice with MPTP‐induced PD, curcumin was reported to alleviate motor deficits and dopaminergic neuron loss, improve intestinal dysbiosis, and restore short‐chain fatty acid levels [385]. Furthermore, in the 6‐OHDA‐induced rat PD model, curcumin increased the survival of tyrosine hydroxylase (TH)‐positive striatal fibers and neurons in the substantia nigra pars compacta, elevated dopamine levels, and reduced abnormal rotational behavior [386]. Given that microglia‐mediated neuroinflammation is closely associated with the pathophysiology of PD, the anti‐inflammatory properties of curcumin likely underlie its significant neuroprotective effects in this disorder. In a rotenone‐induced PD mouse model, curcumin alleviated rotenone‐induced behavioral deficits, mitigated the loss of dopamine neurons in the substantia nigra, suppressed microglial activation, and reduced mitochondrial fission. The neuroprotection afforded by this polyphenol in this model was attributed to its inhibitory effects on the NF‐κB signaling pathway, the NLRP3 inflammasome, and the neuroinflammation mediated by proinflammatory cytokines (IL‐1β, IL‐6, and IL‐18) [387]. In other PD animal models, curcumin has been demonstrated to effectively suppress the upregulation of the NF‐κB signaling pathway and inhibit the expression of proinflammatory cytokines (TNF‐α, IL‐1β, and IL‐6), iNOS, and key regulators of the intrinsic apoptotic pathway (BAX, BCL‐2, Caspase‐3, and Caspase‐9). Furthermore, curcumin treatment reportedly boosted the levels of GSH and oxidoreductases and prevented the aggregation of α‐Syn in dopaminergic neurons [388].

In summary, studies to date have shown that curcumin mitigates PD progression by inhibiting α‐Syn protein aggregation, suppressing the expression of neuroinflammatory factors, alleviating oxidative stress, and modulating the gut microbiota. These multifaceted mechanisms together contribute to its neuroprotective effects.

#### 5.1.5 Multiple Sclerosis

MS is a disease characterized primarily by inflammation and demyelination in the CNS [389]. This condition typically manifests between the ages of 20 and 40 years and shows a higher prevalence in females. The clinical presentations of MS are varied, which complicates its diagnosis. Imaging studies have revealed varying degrees of inflammatory lesions in both the gray and white matter of the brains of affected patients, accompanied by oligodendrocyte death and myelin destruction [390]. In the early stages of the disease, myelin‐specific reactive T cells and peripheral immune cells, such as macrophages, infiltrate the CNS through the compromised blood–brain barrier [391]. T helper 17 (Th17) cells play a significant immunological role in the pathophysiology of MS. Once activated, Th17 cells aberrantly secrete IL‐17 and IL‐22, cross the blood–brain barrier, exacerbate barrier damage, and interfere with neuronal signal transmission by activating inflammatory responses within the CNS. A variety of immunosuppressive and immunomodulatory therapies are widely employed in clinical settings to modulate immune responses, acting through multiple mechanisms. However, these treatments often exhibit limited efficacy and are frequently associated with side effects. Curcumin has demonstrated significant efficacy against various inflammatory diseases, including neurological disorders. In this section, we explore the potential therapeutic effects of curcumin in MS based on both in vitro and in vivo studies.

##### 5.1.5.1 In Vitro Studies

Liu et al. isolated CD4+ T cells from mouse spleens and induced their differentiation into Th1, Th17, and regulatory T cell (Treg) subtypes. Following treatment with varying concentrations of curcumin, they induced apoptosis in these cells by downregulating the expression of the antiapoptotic protein BCL‐2 and upregulating that of the proapoptotic proteins BAX and Caspase‐3, thereby providing a basis for the use of curcumin as a therapeutic agent for MS [392]. In LPS‐stimulated BV‐2 microglial cells, curcumin alleviated inflammatory responses by inhibiting the AXL/JAK2/STAT3 signaling pathway [393]. Astrocytes, the most abundant glial cell type in the brain, play a fundamental role in the maintenance of CNS homeostasis and are closely related to MS when activated in response to stimuli. Furthermore, curcumin treatment was demonstrated to dose‐dependently inhibit MMP9 activity, IL‐6 secretion, and MCP‐1 transcription in LPS‐activated astrocytes, thereby mitigating inflammatory responses and exerting neuroprotective effects [394]. In an in vitro experiment, the treatment of activated T cells with curcumin inhibited the IL‐12‐induced phosphorylation of JAK2 and STAT3, resulting in diminished Th1 cell differentiation, suggesting that curcumin holds potential for application in MS treatment [395].

##### 5.1.5.2 In Vivo Studies

In experimental autoimmune encephalomyelitis (EAE) and copper chloride (CPZ)‐induced MS mouse models, curcumin treatment significantly reduced the infiltration of inflammatory cells and demyelination in the spinal cord, accompanied by an improvement in neurological function. Furthermore, curcumin alleviated oxidative stress, which ultimately promoted remyelination, improved behavioral abnormalities, and repaired tissue damage, thus enhancing MS outcomes [396]. Moreover, curcumin was reported to mitigate EAE‐induced cognitive and motor impairments, oxidative stress, and neuroinflammation through the activation of the AMPK/SIRT1 signaling pathway [397]. Additionally, in CPZ‐induced MS mouse models, curcumin exerted neuroprotective effects by downregulating AXL expression and alleviating astrocyte‐mediated inflammatory responses [398]. In the EAE model, curcumin inhibited the expression of the AXL receptor, thereby blocking the phosphorylation of JAK2 and STAT3, reducing microglia‐mediated neuroinflammation, and alleviating neurological damage in mice [393]. In one study, the authors encapsulated curcumin in nanoparticles to enhance its bioavailability and efficacy following administration via intraperitoneal injection. The evaluation of this formulation in MS animal models revealed that preventive treatment at the onset of EAE symptoms mitigated disease progression and delayed relapse. This intervention also suppressed proinflammatory factor expression in the spinal cord, enhanced anti‐inflammatory and antioxidant effects, reduced demyelination severity, and increased the numbers of neurotrophic factors and progenitor cells involved in myelin repair [399]. Separately, investigators employed high‐density lipoprotein peptide‐phospholipid scaffolds (HPPS) as carriers to encapsulate curcumin (HPPS–Cur). This delivery method effectively reduced the infiltration of inflammatory monocytes across the blood–brain barrier, inhibited microglial proliferation, and restricted the infiltration of other effector immune cells, resulting in a 70% reduction in EAE incidence. This effect was attributed to the immunomodulatory action of HPPS–Cur on inflammatory monocytes, which inhibited NF‐κB activation and downregulated the expression of adhesion and migration‐related molecules [400]. Saravanan et al. demonstrated that curcumin alleviated EAE by modulating Th1/Th17 cell differentiation, inhibiting IL‐12/IL‐23, and upregulating Th2/Treg responses, highlighting the significance of curcumin in the treatment of MS [401]. Furthermore, results from an EAE SJL/J mouse model showed that curcumin alleviated spinal cord inflammatory infiltration and demyelination via the inhibition of IL‐12‐mediated JAK/STAT signaling pathways [395].

In conclusion, curcumin exhibits the potential to suppress neuroinflammation in EAE models, thereby improving the prognosis of MS.

#### 5.1.6 Amyotrophic Lateral Sclerosis

ALS is a neurodegenerative disease characterized by the progressive loss of upper and lower motor neurons, leading to muscle weakness, paralysis, and ultimately respiratory failure within 3–5 years of symptom onset [402]. The global incidence of ALS is approximately 1.75 per 100,000 person‐years, making it the most common motor neuron disease among adults. The pathogenesis of ALS involves a complex interplay between genetic susceptibility—particularly mutations in *SOD1*, TAR DNA‐binding protein, and fused in sarcoma (*FUS*)—and environmental factors. This interplay results in multiple pathological mechanisms, including protein misfolding, oxidative stress, and neuroinflammation. Despite extensive research, treatment options remain limited. Recent studies have confirmed that curcumin can serve as an adjunctive therapy to slow the progression of ALS, offering promising avenues for the treatment of this condition.

##### 5.1.6.1 In Vitro Studies

Bhatia et al. evaluated the protective effects of curcumin nanoparticles against the toxicity of SOD1 aggregates in human monocytic THP‐1 cells. These nanoparticles altered the aggregation process by binding to protein aggregates, leading to the formation of smaller, more disordered, and less toxic aggregates. This structural modification significantly reduced the toxicity to THP‐1 cells, providing a potential direction for the application of curcumin as a therapeutic agent for ALS [403]. Additionally, researchers identified a novel potent polyphenolic compound, bis‐demethoxycurcumin, which effectively reduced the incidence of ALS in experimental models by preventing the abnormal misfolding and aggregation of SOD1 into amyloid‐like aggregates [404]. Moreover, in a motor neuron‐like cell line (NSC‐34), curcumin derivatives reversed TDP‐43‐induced action potential and sodium channel dysfunction. These effects were achieved by alleviating oxidative stress and improving mitochondrial dysfunction, without evidence of cytotoxicity [405].

##### 5.1.6.2 In Vivo Studies

Hajime et al. evaluated the effects of curcumin derivatives on the H46R mutant SOD1 transgenic mouse model of ALS. They revealed that the administration of these derivatives significantly slowed the progression of motor dysfunction and reduced the accumulation of highly aggregated SOD1, further protecting neurons in the spinal cord of the mice [406]. Furthermore, Cur–SLNs have shown potential as an estrogen substitute therapy in the Prp–TDP–43A315T transgenic mouse model of ALS. Cur–SLNs improved the survival rate of ALS mice through the modulation of the enzymatic activities of CYP19A1 and CYP3A4, thereby suppressing TDP‐43 aggregation [407].

In summary, these findings indicate that curcumin significantly improves ALS symptoms both in vitro and in vivo, providing a novel therapeutic direction for enhancing ALS prognosis.

### 5.2 Cancer

#### 5.2.1 Colon Cancer

Colorectal cancer, a prevalent malignancy of the digestive system, exhibits a high global incidence and ranks among the primary causes of cancer mortality [408]. While primarily affecting middle‐aged and older populations, evolving lifestyle factors indicate a discernible trend toward earlier onset. Clinical presentations of colorectal cancer are strongly correlated with tumor location; right‐sided colon cancers often manifest as anemia, fatigue, and nonspecific abdominal discomfort, whereas left‐sided colon cancers are more frequently associated with altered bowel habits, rectal bleeding, and stool caliber reduction. Diagnostic imaging may identify colonic wall lesions of varying sizes, characterized by mucosal erosion and mural infiltration. During disease progression, immune cells within the tumor microenvironment, including tumor‐associated macrophages and Tregs, facilitate immune escape, thereby fostering tumor proliferation and metastasis [409]. Current clinical practice employs comprehensive treatment modalities such as surgical resection, chemotherapy, radiotherapy, and immunotherapy; however, the effectiveness of these interventions is frequently constrained by drug‐induced toxicity and acquired resistance. Research has indicated that curcumin holds promise as a therapeutic agent for diverse gastrointestinal malignancies [410]. Consequently, this section will provide a systematic review of curcumin's potential therapeutic applications in both in vitro and in vivo investigations of colorectal cancer.

##### 5.2.1.1 In Vitro Studies

Using CT26 and RKO colorectal cancer cell lines, Zhou et al. demonstrated curcumin's inhibitory effect on cell viability (IC_50_ values of 23.52–13.62 and 26.3–14.22 µM, respectively), its induction of apoptosis, and its causation of G_2_ phase arrest, utilizing cell viability assays and apoptosis analysis [411]. Guéguinou et al. [412] employing MSI colorectal cancer cell lines like HCT116 and methods such as mitochondrial function assays, concluded that curcumin induces apoptosis by inhibiting the mitochondrial sodium‐calcium‐lithium exchanger, resulting in mitochondrial calcium overload, elevated ROS, membrane depolarization, and diminished ATP production. Wang et al. [413] utilized human colorectal cancer cell lines, including HCT116, and employed techniques like noncoding RNA regulation and signaling pathway analysis to establish that curcumin inhibits CRC cell proliferation, migration, and metastasis by modulating noncoding RNA to downregulate heparanase expression, suppress the IL‐6/STAT5 signaling pathway, and disrupt the tumor microenvironment and epithelial–mesenchymal transition (EMT) process. Elbadawy et al. [414] employed human colorectal cancer cell lines and methods including ERK phosphorylation and tumor stem cell marker detection to further show that amorphous curcumin inhibits CRC by suppressing ERK phosphorylation and its downstream c‐MYC and cyclin‐D1 signaling, reducing tumor stem cell markers CD44, LGR5, and CD133, and inducing cell cycle arrest and apoptosis.

##### 5.2.1.2 In Vivo Studies

In a mouse xenograft model of MSI colorectal cancer, Guéguinou et al. [412] observed that curcumin impedes tumor progression and augments apoptosis by downregulating the NCLX target. This mechanism induces mitochondrial calcium overload, elevates ROS, causes membrane depolarization, and reduces ATP levels, as evidenced by mitochondrial function assays. The study also indicated a significant correlation between elevated NCLX expression and recurrence risk in MSI patients. Elbadawy et al. [414] employing a mouse xenograft model derived from human colorectal cancer organoids, utilized techniques such as ERK phosphorylation and tumor stem cell marker detection. Their findings showed that amorphous curcumin suppresses ERK phosphorylation and its downstream c‐MYC and cyclin‐D1 signaling pathways, leading to reduced expression of tumor stem cell markers (CD44, LGR5, CD133). This inhibition results in cell cycle arrest, apoptosis, necrotic lesions, and an overall antitumor effect. A systematic review of 22 animal studies by Jia et al. which analyzed colorectal cancer animal models comprehensively, corroborated that curcumin significantly reduces tumor volume and weight. Its mechanisms include modulating gut microbiota, mitigating intestinal inflammation, inhibiting cell proliferation, promoting apoptosis, preventing metastasis, and enhancing chemotherapy sensitivity. Notably, oral administration of curcumin demonstrated superior antitumor efficacy compared with other routes [415].

The therapeutic efficacy of curcumin in colorectal cancer, as supported by the cited research, is principally mediated by several mechanisms: suppression of cell viability, induction of cell cycle arrest and apoptosis, modulation of mitochondrial calcium homeostasis and ROS metabolism, regulation of noncoding RNAs and associated signaling pathways, and reduction of tumor stem cell markers. Additionally, its capacity to inhibit tumor progression and improve chemosensitivity has been corroborated in in vivo experimental models.

#### 5.2.2 Breast Cancer

Breast cancer, a highly prevalent malignancy in women, ranks globally as the most frequent cancer incidence and a primary cause of cancer‐related mortality [416]. While predominantly affecting individuals over 40 years of age, lifestyle shifts are contributing to an increasing incidence in younger demographics. The clinical presentation of breast cancer is varied, with early, nonspecific symptoms complicating early diagnosis. Examination via imaging and histopathology identifies malignant proliferative lesions of varying severity within breast tissue, modulated by molecular subtypes linked to hormone receptor status (ER/PR/HER2) [417]. The tumor microenvironment exhibits enhanced infiltration of tumor‐associated macrophages polarized to an M2 phenotype, fostering an immunosuppressive milieu that facilitates tumor advancement. Current clinical management employs surgery, chemoradiotherapy, endocrine therapy, and targeted therapy; however, therapeutic resistance and treatment‐induced toxicity persist as considerable clinical obstacles. Research has substantiated curcumin's cardioprotective role in adjuvant breast cancer therapy and its capacity to ameliorate tamoxifen‐induced hepatotoxicity. Consequently, this review will investigate curcumin's prospective therapeutic utility in breast cancer management.

##### 5.2.2.1 In Vitro Studies

In a study utilizing the MDA‐MB‐231 triple‐negative breast cancer cell line and whole‐genome methylation sequencing, Nirgude et al. [418] identified that curcumin derivatives ST08 and ST09 induce methylation changes in promoter regions, including the HOX family and genes such as CACNAH1 and CDH23. These modifications were shown to modulate the ECM signaling pathway and cell cycle pathway, respectively, leading to antiproliferative, proapoptotic, and antimigratory effects. Hasan et al. [419] explored the use of CS‐coated salmon lecithin‐derived nanoliposomes for curcumin delivery in the MCF‐7 human breast cancer cell line. This nanocarrier achieved an 88% drug loading efficiency and, at 20 µM, reduced cell growth fourfold compared with controls. The enhanced antiproliferative activity was attributed to the nanocarrier's improved curcumin bioavailability. Pourmadadi et al. [420] developed a curcumin‐loaded Fe_3_O_4_/CS/agarose dual‐emulsion hydrogel nanocarrier, also using the MCF‐7 cell line. This nanocomposite demonstrated sustained and pH‐responsive release properties, promoting a high rate of cell apoptosis, with its antitumor efficacy linked to the enhanced delivery of curcumin by the hydrogel nanocarrier. Romero et al. [421] investigated the T47D human breast cancer cell line using carbon dots synthesized from curcumin as a precursor, in conjunction with blue light irradiation. Under irradiation, these carbon dots functioned as both photosensitizers and photothermal agents, eliciting significant photodynamic cytotoxic effects through the generation of ROS and localized heating (40–50°C) [421].

##### 5.2.2.2 In Vivo Studies

In a study employing a 4T1 tumor‐bearing mouse model, Zhao et al. investigated a curcumin‐mediated photodynamic therapy strategy. Their findings indicate that this therapy generates ROS, leading to the inactivation of the mitochondrial outer membrane protein TOM70. This disruption impedes MIC60 transport into mitochondria, causing significant mitochondrial damage and effectively inhibiting breast cancer progression [422]. In a NOD scid gamma mouse model engrafted with MDA‐MB‐231 cells, Wang et al. administered luteolin (10 mg/kg/day) and curcumin (20 mg/kg/day) via intraperitoneal injection every other day for 5 weeks. Through transcriptomic sequencing, Western blotting, and histopathological analysis, the study revealed that the combination of luteolin and curcumin synergistically activated the Type I interferon signaling pathway, attenuated the TGF‐β signaling pathway, and downregulated c‐Myc and Notch1 protein levels, leading to effective suppression of tumor growth in triple‐negative breast cancer [423].Further research by Li et al. using a triple‐negative breast cancer mouse model (4T1 cell implantation) and curcumin intervention focused on the SLC7A11/NF‐κB/TGF‐β signaling pathway. They demonstrated that curcumene inhibits tumor growth by downregulating this pathway, reversing EMT, reducing lung metastasis, and inducing ferroptosis, characterized by decreased MMP and increased accumulation of lipid ROS [424].

Curcumin and its analogs exhibit antitumor activity in breast cancer through epigenetic alterations, improved nanodelivery and photoresponsive characteristics, modulation of ECM and cell cycle pathways, and suppression of Hedgehog/Gli1 and SLC7A11/NF‐κB/TGF‐β signaling. These mechanisms collectively inhibit proliferation, migration, and EMT, while inducing apoptosis and ferroptosis, thereby demonstrating potent anticancer effects across in vitro and in vivo experimental models.

#### 5.2.3 Skin Cancer

Skin cancer, a prevalent malignant neoplasm globally, leads all cancers in incidence and is a primary driver of dermatological mortality [425]. The disease predominantly affects individuals over 50 years of age, especially those with prior ultraviolet (UV) radiation exposure, who exhibit a markedly elevated risk. Clinical presentations of skin cancer are varied, potentially manifesting in early stages as new cutaneous growths, alterations in pigmented nevi, ulcerations, or nodules. Advanced disease can involve local ulceration, hemorrhage, and regional lymph node metastasis, complicating early detection [426]. Imaging and histopathological analyses indicate varying degrees of malignant proliferation within cutaneous tissues, characterized by aberrant melanocyte proliferation, basal cell malignant transformation, and dysregulated activation of multiple oncogenic signaling cascades. Within the tumor microenvironment, impaired function of tumor‐associated immune cells and a perturbation of proinflammatory and anti‐inflammatory mediators establish an immunosuppressive milieu, thereby facilitating tumor progression and dissemination. Current investigations have established curcumin's potential therapeutic utility in managing skin cancer. A meta‐analysis of 40 studies encompassing 989 animal subjects revealed that curcumin significantly attenuated tumor volume and mass in melanoma models. Consequently, this section will investigate curcumin's potential therapeutic efficacy as demonstrated in in vitro and in vivo investigations of skin cancer.

##### 5.2.3.1 In Vitro Studies

Szlasa et al. utilized curcumin‐mediated photodynamic therapy on A375 and C32 cell lines, observing a reduction in cell viability to 8.98–12.42% at 50 µM and 48 h, accompanied by a significant increase in apoptotic and necrotic cells. However, this approach lacked selectivity for normal skin cells [427]. In studies with a human cSCC cell line, Khan et al. demonstrated that curcumin modulates Skp2, CDKI, and KLF4 expression. Specifically, it inhibits Skp2, upregulates p21 and KLF4, and suppresses cell proliferation, colony formation, and spheroid formation through ROS‐mediated apoptotic pathways. Furthermore, curcumin enhances sensitivity to chemotherapeutic agents like cisplatin and doxorubicin [428]. Trochopoulos et al. [429] using CTCL cell lines, investigated the combination of micellar curcumin with erufosine. Their analysis of signaling pathways and antibacterial activity confirmed synergistic antitumor effects by inhibiting TWIST1 and Akt expression, reducing NF‐κB activation, inducing apoptosis, and regulating GSH levels. This combination also demonstrated enhanced anti‐Staphylococcus activity and inhibited biofilm formation.

##### 5.2.3.2 In Vivo Studies

Fontes et al. [430] administered intraperitoneal curcumin (20 mg/kg) and disulfiram (60 mg/kg) to C57BL/6 mice bearing B16‐F10 melanoma. This combination regimen synergistically elevated mitochondrial superoxide and enhanced oxidative stress, resulting in significant tumor growth inhibition. Specifically, the combination group achieved a 28.8% tumor inhibition rate, surpassing monotherapy groups (17.0–19.8%). The regimen also effectively induced apoptosis and DNA fragmentation. In a separate study on a subcutaneous B16‐F10 melanoma mouse model, Ricken et al. combined curcumin and silver nanoparticles within DPPC–F127 liposomes and applied photodynamic therapy. This drug delivery system significantly inhibited tumor growth and improved survival in tumor‐bearing mice by augmenting singlet oxygen production [431]. Malathi et al. [432] investigated TPGS‐stabilized PLGA nanopatterned films loaded with curcumin in a DMBA/castor oil‐induced squamous cell carcinoma model in Swiss albino mice. The study demonstrated that these nanopatterned films inhibited skin tumor progression (mechanism unspecified), and in vitro assays showed potent cytotoxic activity against the A431 human squamous cell carcinoma cell line.

### 5.3 Autoimmune Diseases

#### 5.3.1 Rheumatoid Arthritis

Rheumatoid arthritis (RA) is a chronic autoimmune inflammatory condition defined predominantly by symmetrical polyarticular synovitis. Affecting approximately 0.5–1.0% of the global population, RA significantly contributes to joint deformity and functional disability. This condition disproportionately impacts women between the ages of 30 and 50 years, with a notable increase in risk among individuals with a familial predisposition. Initial RA symptoms include prolonged morning stiffness, joint swelling, and pain; delayed intervention can lead to joint erosion and irreversible functional deficits [433]. Diagnostic evaluations, including imaging and serological assays, may indicate joint space narrowing, bone erosion, and the presence of rheumatoid factor and anti‐CCP antibodies. During disease progression, M1 macrophages, Th17 cells, and activated fibroblast‐like synoviocytes infiltrate the synovium. These cells perpetuate inflammation by secreting mediators like TNF‐α, IL‐6, and MMPs, which promote synovial hyperplasia and cartilage degradation [434]. Current therapeutic strategies for RA primarily involve NSAIDs, DMARDs, biologics, and targeted synthetic agents, though prolonged treatment may increase infection susceptibility and lead to drug resistance [435]. Research has indicated that curcumin exhibits anti‐inflammatory, immunomodulatory, and chondroprotective properties in RA models through the modulation of signaling pathways such as NF‐κB and STAT3. This review will comprehensively detail the pharmacological actions and underlying mechanisms of curcumin in both in vitro and in vivo investigations of RA.

##### 5.3.1.1 In Vitro Studies

Research by Yan et al. employing a RAW264.7 macrophage model demonstrated that the codelivery of prednisolone and curcumin via human serum albumin nanoparticles resulted in a synergistic suppression of proinflammatory factors and an elevation in the anti‐inflammatory factor IL‐10 [436]. In a separate study, Wang et al. [437] utilized RAW264.7 macrophages to show that ROS‐responsive, folate‐modified curcumin micelles specifically target and activate macrophages, consequently inhibiting inflammatory factor release and osteoclast differentiation. Chen et al. [438] working with primary rat articular chondrocytes, observed that curcumin mitigates IL‐1β‐induced chondrocyte apoptosis through autophagy activation and NF‐κB signaling pathway inhibition. Furthermore, He et al. [320] utilized the rabbit HIG‐82 synovial cell line and demonstrated that curcumin‐loaded exosomes derived from mesenchymal stem cells improve stability and cellular uptake. This approach synergistically inhibited the expression of antiapoptotic proteins IAP1/IAP2 and inflammatory factors, including IL‐6, TNF‐α, MMP1, and PGE2, thereby more effectively inducing synovial cell apoptosis. Collectively, these findings suggest that curcumin exerts anti‐RA effects in vitro by modulating inflammatory responses, apoptosis, and autophagy pathways via multiple molecular targets.

##### 5.3.1.2 In Vivo Studies

Preclinical investigations have corroborated the therapeutic efficacy of curcumin in RA models. Hemshekhar et al. demonstrated in a collagen‐induced arthritis (CIA) mouse model that highly bioavailable curcumin attenuated cathelicidin (CRAMP) and calprotectin (S100A8/S100A9) levels within joint tissues. These reductions were found to correlate linearly with serum levels of TNF‐α, IFN‐γ, and MCP‐1, indicating curcumin's capacity to mitigate both localized and systemic inflammation [439]. In a CIA rat model, Sun et al. established that curcumin‐mediated photodynamic therapy induces ferroptosis in synovial fibroblasts via the downregulation of the Nrf2/xCT/GPX4 pathway, consequently ameliorating joint inflammation, synovial hyperplasia, and collagen deposition [440]. Furthermore, Wang et al. utilized a CIA rat model to show that ROS‐responsive folate‐modified curcumin micelles enhance drug circulation time, exhibit targeted accumulation in inflamed joints, and suppress serum inflammatory factors and cartilage degradation, thereby effectively controlling RA progression [437].

In summary, the therapeutic efficacy of curcumin in RA is principally mediated by the inhibition of synovial cell proliferation and the release of inflammatory cytokines. Additional mechanisms include the induction of autophagy and ferroptosis, modulation of signaling cascades (e.g., NF‐κB and Nrf2/xCT/GPX4), and augmentation of anti‐inflammatory cytokine IL‐10 expression. These observed effects, encompassing the targeting of inflamed joints, amelioration of cartilage degradation, and suppression of systemic inflammation, have been substantiated in vivo.

#### 5.3.2 Systemic Lupus Erythematosus

SLE is a chronic autoimmune disorder marked by immune system dysregulation, affecting an estimated 3.4 million individuals globally. This condition is a primary driver of multiorgan damage and premature mortality [441], disproportionately impacting women of reproductive age and exhibiting higher incidence in non‐Hispanic Black populations compared with White populations. SLE presents with significant clinical heterogeneity, manifesting across multiple organ systems, including renal (proteinuria, hematuria, progressive renal dysfunction in lupus nephritis), cutaneous (malar rash), neurological, and cardiovascular systems, with neuropsychiatric lupus potentially causing cognitive deficits and seizures [442]. Pathogenically, aberrant Type I interferon pathway activation and autoreactive T and B cell dysfunction precipitate autoantibody formation and immune complex deposition. This subsequently activates complement and inflammatory pathways, leading to damage in renal, cutaneous, cardiovascular, and neuropsychiatric tissues. Current standard first‐line therapy with hydroxychloroquine effectively mitigates disease activity and mortality; for moderate to severe cases, combination therapy with immunosuppressants and biologics such as mycophenolate mofetil, azathioprine, cyclophosphamide, and belimumab may be employed [443]. Curcumin has demonstrated immunomodulatory and anti‐inflammatory properties relevant to SLE treatment. Consequently, this section will investigate curcumin's therapeutic potential in managing SLE.

##### 5.3.2.1 In Vitro Studies

Zhao et al. demonstrated in murine renal podocyte cell line studies that curcumin substantially inhibits NLRP3 inflammasome activation and the resultant Caspase‐1/IL‐1β signaling pathway triggered by anti‐dsDNA antibodies. This inhibition serves to attenuate podocyte injury and ameliorate lupus nephritis [444]. Concurrently, Handono et al. [445] isolated CD4+ T cells from both SLE patients and healthy donors. Employing flow cytometry and ELISA, their findings indicated that low‐dose curcumin (0.1–1 µg/mL) specifically reduced the proportion of Th17 cells and IL‐17A secretion from SLE patient CD4+ T cells, while concurrently augmenting Treg cell proportion and TGF‐β1 expression. This modulation effectively rebalanced the Th17/Treg immune equilibrium. Notably, these effects were not observed in cells from healthy controls.

##### 5.3.2.2 In Vivo Studies

In MRL/lpr and R848‐induced lupus mouse models, flow cytometry and RNA sequencing by Xu et al. demonstrated that curcumin administration promoted double‐negative T cell (DNT cell) apoptosis, restored T cell subset homeostasis, and subsequently improved serological and immunological parameters while ameliorating lupus nephritis‐associated pathological damage [446]. In the NZBWF1 lupus mouse model, Dent et al. observed via oral administration and renal function assessment that curcumin effectively reduced spleen weight, decreased glomerulosclerosis, and lowered blood urea nitrogen levels, maintaining stable body weight, thus attenuating autoimmune activity and renal injury [447].

In summary, the therapeutic efficacy of curcumin in SLE is principally mediated by the inhibition of NLRP3 inflammasome activation and the resultant Caspase‐1/IL‐1β signaling cascade. Furthermore, curcumin modulates the Th17/Treg immune cell equilibrium and induces apoptosis in double‐negative T‐cells. These mechanisms, supported by observed enhancements in serological and immunological indices and a reduction in renal pathology and autoimmune phenomena, have been substantiated in preclinical in vivo studies.

#### 5.3.3 Psoriasis

Psoriasis is characterized as a chronic, inflammatory dermatosis, etiologically linked to a confluence of genetic predispositions and environmental triggers. Affecting an estimated 2–3% of the global population, it constitutes a significant contributor to epidermal barrier impairment and arthropathy [448]. The disease typically presents with bimodal peaks in incidence, observed most prominently in individuals between 15–35 and 50–60 years of age, with notable interethnic variability. Clinically, it manifests as well‐demarcated erythematous plaques adorned with silvery‐white scales, predominantly localized to the scalp, elbows, knees, and lumbosacral area. In more severe presentations, the development of pustules, erythroderma, and psoriatic arthritis can complicate timely diagnosis [449]. Diagnostic modalities including dermoscopy and histopathological analysis reveal epidermal dyskeratosis, acanthosis, and elongation of dermal papillae, alongside associations with susceptibility loci such as HLA‐C and dysregulation within the IL‐23/Th17 axis. Disease pathogenesis involves a positive feedback cascade among DCs, Th17 lymphocytes, and keratinocytes, promoting hyperkeratinization and sustaining an inflammatory milieu. Current therapeutic strategies for mild psoriasis predominantly employ topical corticosteroids, vitamin D analogs, and novel nonsteroidal agents. For moderate‐to‐severe disease, systemic agents such as methotrexate, cyclosporine, apremilast, or biologic therapies targeting TNF‐α, IL‐17, or IL‐23 may be indicated [450]. Curcumin possesses demonstrated anti‐inflammatory, antioxidant, and immunomodulatory properties relevant to psoriasis management. Accordingly, this section will delineate the potential therapeutic utility of curcumin in the treatment of psoriasis.

##### 5.3.3.1 In Vitro Studies

In studies utilizing THP‐1 macrophages and NCTC‐2544 keratinocytes, Serini et al. demonstrated via ELISA and cell viability assays that curcumin–linolenic acid SLNs effectively suppress imiquimod (IMQ)‐induced inflammatory factor expression, keratinocyte proliferation, apoptosis, and ferroptosis markers, ameliorating psoriasis‐like inflammation [451]. Xu et al. [452] employing HaCaT keratinocytes and macrophage cell lines, confirmed through cell viability assays, inflammatory cytokine, and histological staining that curcumin nanocomposites reduce TNF‐α, IL‐17, IL‐22, and IL‐23 expression, thereby mitigating keratinocyte hyperproliferation, inflammatory responses, and improving psoriasiform lesions. Furthermore, Niu et al. [453] utilizing an M5‐stimulated HaCaT keratinocyte model, curcumin improves psoriasiform lesions by inhibiting the PI3K–Akt signaling pathway, consequently downregulating excessive keratinocyte proliferation and inflammatory cytokine expression.

##### 5.3.3.2 In Vivo Studies

In a murine model of psoriasis, Wu et al. established a micromot‐induced psoriatic mouse model and utilized histopathology and ELISA to show that Cur–TPP, a mitochondria‐targeted curcumin derivative, effectively suppressed epidermal hyperplasia, reduced Ki67‐positive cell counts, and downregulated IL‐17 and TNF‐α levels, thereby ameliorating psoriasiform inflammation [454]. Zhang et al. employed a psoriatic mouse model and supercritical solvent‐free fluidized‐bed coating technology to formulate curcumin–POPG phospholipid complex‐coated sponge bone needles. Their findings indicated that these needles effectively penetrated the stratum corneum, released nanoparticles to inhibit DC activation, and induced regulatory T‐cell proliferation, consequently alleviating psoriasis symptoms [455]. Similarly, Kumar et al. utilized a psoriasis‐induced mouse model and methods including PASI scoring, ELISA, and tissue staining to observe that a curcumin solid‐lipid nanoparticle gel significantly downregulated IFN‐α, IL‐23, IL‐17, and TNF‐α levels and improved dermal tissue architecture, thus effectively mitigating psoriasiform inflammation [456].

#### 5.3.4 Osteoarthritis

Osteoarthritis, the most prevalent chronic joint disease, exhibits a rising global incidence attributed to an aging demographic and increased obesity rates, resulting in significant joint pain and disability [457]. Predominantly affecting women over 50 years of age, knees, hips, and hands are the most commonly involved joints. Initial presentations are often subtle, characterized by pain associated with activity, transient morning stiffness, and functional impairments. With disease progression, joint effusion, deformity, and restricted range of motion may emerge, complicating early detection [458]. Diagnostic modalities, including imaging and pathological assessments, reveal hallmark findings such as joint space narrowing, osteophyte formation, subchondral sclerosis, and synovial inflammation. Advanced stages involve a vicious cycle of chondrocyte senescence, synovial inflammation, and subchondral bone remodeling, which exacerbates cartilage matrix degradation and joint destruction. Current therapeutic strategies encompass nonpharmacological interventions, oral analgesics (acetaminophen, NSAIDs), and intra‐articular injections; arthroplasty is reserved for severe presentations, though chronic medication use is associated with gastrointestinal and cardiovascular adverse events [459]. Curcumin demonstrates anti‐inflammatory, antioxidant, and chondroprotective properties relevant to osteoarthritis. Consequently, this section will investigate the therapeutic potential of curcumin in managing osteoarthritis.

##### 5.3.4.1 In Vitro Studies

In studies utilizing IL‐1β‐induced primary rat chondrocytes and molecular docking, Xu et al. [460] determined that curcumin mitigates chondrocyte injury and decelerates osteoarthritis progression by upregulating SIRT5 expression and facilitating ACSL4 deacetylation, consequently inhibiting the ferroptosis pathway. Employing human chondrosarcoma SW‐1353 cells, Ghiselli et al. [461] leveraged molecular docking, cAMP quantification, and ROS detection to establish that turmeric resin ameliorates oxidative stress and inflammatory responses via activation of cannabinoid receptor 2 (CB2), inhibition of NF‐κB and ERK signaling, and downregulation of IL‐6 and COX‐2 expression, thereby improving osteoarthritis‐like lesions. Further investigation involving primary chondrocytes and an IL‐1β‐induced inflammatory model, revealed that curcumin targets the TRPM2/NLRP3 signaling axis, suppressing pyroptosis‐related protein expression and inflammatory factor secretion, thus alleviating chondrocyte damage [462].

##### 5.3.4.2 In Vivo Studies

Hridayanka et al. established an osteoarthritis model in adult male Sprague–Dawley rats via intra‐articular injection of sodium iodoacetate into the knee joint. Employing histopathology and cell coculture, they observed that curcumin NE attenuated joint edema, suppressed cartilage degradation and synovial inflammation, and diminished macrophage infiltration, thereby ameliorating the osteoarthritis microenvironment [463]. Jhun et al. [464] employed a monosodium iodoacetate‐induced osteoarthritis model in Wistar rats. Behavioral assessments, histopathology, and micro‐computed tomography (micro‐CT) revealed that curcumin synergized with Omega‐3 fatty acids to alleviate pain, preserve cartilage architecture, downregulate MMP expression, and enhance mitochondrial function markers, consequently retarding osteoarthritis progression. Zheng et al. [465] utilized a C57BL/6 mouse model of osteoarthritis induced by medial meniscal instability. Through behavioral testing, micro‐CT, and histopathological analysis, they determined that the curcumin C3 complex, in conjunction with chondroitin sulfate and glucosamine, effectively maintained subchondral bone microstructural integrity, decreased osteoarthritis research society international (OARSI) histological scores, and improved locomotor function.

### 5.4 Cardiovascular Diseases

#### 5.4.1 Heart Failure

Heart failure is the end stage of numerous cardiovascular diseases, characterized by impaired cardiac pumping function that fails to meet the metabolic demands of the body [466]. Based on left ventricular ejection fraction, heart failure can be classified into heart failure with reduced ejection fraction and heart failure with preserved ejection fraction [467]. Myocardial fibrosis, cardiomyocyte hypertrophy, energy metabolism disorders, and apoptosis are key pathological mechanisms in the progression of heart failure [468]. Curcumin and its derivatives regulate these pathological processes through multiple targets, demonstrating potential for the treatment of heart failure.

##### 5.4.1.1 In Vitro Studies

Cardiomyocyte hypertrophy is an important feature of pathological cardiac remodeling in heart failure. Pyrazole curcumin reduces CDK9 kinase activity by disrupting the interaction between CDK9 and Cyclin T1, thereby indirectly inhibiting p300 HAT activity and RNA polymerase II transcriptional elongation, and suppressing phenylephrine induced cardiomyocyte hypertrophy at a lower concentration than curcumin [469]. Liposomes co loaded with SGLT2 inhibitors and curcumin, constructed using PVP coating and oleanolic acid to stabilize the lipid bilayer, exhibited sustained and delayed drug release properties in vitro, suggesting their potential as multifunctional targeted platforms for heart failure therapy [470]. Nicotinic acid–curcumin (NC) inhibited LPS induced inflammation and exosome secretion in HUVECs, regulated the levels of miR 142 3p, miR 126 3p and miR 143 3p in exosomes, and thereby protected vascular smooth muscle cell function [471].

##### 5.4.1.2 In Vivo Studies

Curcumin and its derivatives have shown significant protective effects in animal models of heart failure. BDMC competitively inhibited the binding of TGF‐β to TGFBR1, blocked SMAD2/3 phosphorylation, suppressed cardiac fibroblast activation, reduced myocardial fibrosis, and improved cardiac function in mouse models of heart failure with preserved ejection fraction (HFpEF) [472]. In a doxorubicin‐induced cardiotoxicity rat model, curcumin pretreatment upregulated apelin expression and downregulated NF‐κB mRNA expression in heart tissue, significantly ameliorated electrocardiographic QT interval prolongation, reduced cardiac troponin levels, and alleviated oxidative stress and histopathological damage, thereby exerting cardioprotective effects [473]. The novel curcumin analog SJ‐12 reduced the O‐GlcNAcylation level of SP1 by inhibiting OGT activity, thereby stabilizing SERCA2a and regulating calcium signaling pathways; it alleviated cardiac hypertrophy and fibrosis and improved cardiac function in a STZ‐induced diabetic cardiomyopathy mouse model, and the antifibrotic effect was absent in SERCA2a‐ or OGT‐silenced cardiomyocytes, indicating that SJ‐12 exerts cardioprotection by targeting the OGT/SP1/SERCA2a axis [474]. THC regulated the Nrf2–SIRT3 signaling pathway, reduced ROS generation, preserved mitochondrial function, and thereby attenuated postinfarction cardiac dysfunction, infarct size, myocardial fibrosis, cardiac hypertrophy, and cardiomyocyte apoptosis in mice; these protective effects were reversed in Nrf2‐ or SIRT3‐silenced cardiomyocytes [475]. The novel amorphous curcumin formulation ASD–Cur exhibited significantly higher bioavailability than Theracurmin in both rats and humans, and achieved comparable improvement in postinfarction heart failure at a lower dose [476]. In a Dahl salt‐sensitive rat model of hypertension‐induced left ventricular hypertrophy, curcumin inhibited p300 HAT activity, reduced GATA4 acetylation levels, and significantly attenuated left ventricular hypertrophy without affecting blood pressure or systolic function [477].

#### 5.4.2 Atherosclerosis

Atherosclerosis is a chronic inflammatory vascular disease characterized by lipid deposition, inflammatory cell infiltration, foam cell formation, and plaque development [478]. Curcumin shows potential in the prevention and treatment of atherosclerosis through multiple mechanisms, including anti‐inflammatory, antioxidant, and lipid‐regulating effects.

##### 5.4.2.1 In Vitro Studies

In vitro studies have shown that osteopontin targeted liposomes co delivering curcumin and perfluoro n pentane achieved acoustic cavitation induced rupture and rapid curcumin release upon exposure to low intensity focused ultrasound, significantly reduced IL 1α and TNF‐α levels in foam cells, induced apoptosis, and exerted anti‐inflammatory effects by regulating the TLR, NF‐κB, and MAPK3 signaling pathways [479]. Iron curcumin coordination polymers (Fe Cur CPs), constructed by coordinating curcumin with ferric ions, effectively scavenged excessive ROS in endothelial cells and macrophages, reduced monocyte adhesion, attenuated the production of inflammatory factors, and inhibited foam cell formation in vitro [254].

##### 5.4.2.2 In Vivo Studies

In animal models of atherosclerosis, iron curcumin coordination polymers (Fe Cur CPs) administered intravenously to ApoE/mice significantly reduced plaque burden, suppressed macrophage aggregation, decreased ROS and inflammatory cytokine levels, enhanced collagen deposition and α SMA expression, and downregulated MMP9, thereby synergistically stabilizing atherosclerotic plaques [480]. BDMC promoted autophagy by inhibiting the PDK1/Akt/mTOR signaling pathway in vitro, reduced lipid accumulation in ox‐LDL stimulated vascular smooth muscle cells, and alleviated inflammation and lipid accumulation in ApoE/mice in vivo, thus exerting anti atherosclerotic effects [481]. The novel curcumin analog CACN136 regulated iNOS and Arg‐1 expression in LPS induced RAW264.7 cells, reduced cholesterol levels in ox‐LDL induced foam cells, and modulated ABCA1/CD36/SRA1 expression in vitro; in ApoE/mice, it significantly ameliorated aortic plaques and reduced lipid and inflammatory levels through regulating macrophage polarization and lipid metabolism, with superior safety and efficacy compared with the same dose of simvastatin [482]. Sparstolonin B (SSNB) and curcumin modulated Th17 stromal cell interactions via the IL‐17RA/TAK1/NF‐κB pathway; SSNB primarily targeted TLR4/CD36 to inhibit DC activation, Th17 differentiation, and vascular smooth muscle cell inflammation, and inhibited TAK1 phosphorylation, whereas curcumin more markedly suppressed macrophage inflammation and directly inhibited NF κB p65 phosphorylation; together, they reduced aortic plaque lesions and inflammatory infiltration in ApoE/mice [483]. Cadmium exposure exacerbated atherosclerotic plaque formation in ApoE /mice by inducing gut dysbiosis, increasing trimethylamine N oxide (TMAO) synthesis, and promoting M1 type macrophage polarization; curcumin gavage reversed the cadmium‐induced pathological progression by restoring the gut microbiota [484]. Curcumin promoted TFEB nuclear translocation, blocked P300‐mediated BRD4‐dependent super enhancer formation and liquid–liquid phase separation, thereby restoring autophagy, optimizing lipid catabolism, and inhibiting inflammation in ox‐LDL treated foam cells; the key role of the TFEB P300 BRD4 axis was validated in ApoE/mice via bone marrow transplantation [485]. In a mouse model of atherosclerosis complicated by UC, curcumin inhibited the TLR4/PAD4 signaling pathway, reduced the formation of macrophage extracellular traps (METs), decreased the levels of inflammatory cytokines and the collagen degrading enzyme MMP9, thereby reducing necrotic core size, increasing collagen content, and significantly stabilizing vulnerable plaques [486].

#### 5.4.3 Atrial Fibrillation

Atrial fibrillation is the most common sustained cardiac arrhythmia, characterized by disordered atrial electrical activity and rapid, irregular excitation. Atrial fibrosis is a key pathological basis for the development and maintenance of atrial fibrillation [487]. Curcumin shows promise in the prevention and treatment of atrial fibrillation through its antifibrotic effects.

##### 5.4.3.1 In Vitro Studies

In vitro studies have shown that curcumin inhibits the activation, proliferation, and differentiation of cardiac fibroblasts into myofibroblasts, reduces collagen synthesis, and thereby suppresses the progression of atrial fibrosis. In porcine primary hepatocytes, coadministration of curcumin with warfarin downregulated warfarin‐induced CYP3A protein expression and inhibited the enzymatic activities of CYP1A2 and CYP3A, indicating a food–drug interaction between curcumin and warfarin [488].

##### 5.4.3.2 In Vivo Studies

Curcumin exhibits antifibrotic and antiarrhythmic effects in animal models of atrial fibrillation. Curcumin reduced the expression of mature‐mt_tRNA‐Val‐TAC_CCA_end in the atria of aged mice, decreased ROS levels, inflammatory responses, and cellular senescence, thereby ameliorating age‐related atrial fibrosis and reducing atrial fibrillation susceptibility [489]. By integrating data mining and network pharmacology, TGFBR1 was identified as a key gene in valvular atrial fibrillation; molecular docking confirmed a good binding affinity between curcumin and TGFBR1, and curcumin significantly reduced myocardial fibrosis by modulating the TGFβ/Smad signaling pathway, thereby improving valvular atrial fibrillation [490]. Curcumin inhibited p300 expression, blocked the formation of the p53/Smad3 complex, reduced the senescence ratio of atrial fibroblasts, and attenuated atrial fibrosis and atrial fibrillation susceptibility in aged mice and elderly patients with atrial fibrillation [491]. Curcumin gavage significantly shortened the duration of atrial fibrillation, reduced left atrial fibrosis, and decreased serum levels of IL‐17A, IL‐1β, IL‐6, and TGF‐β1 in an Ach‐CaCl_2_‐induced rat model of atrial fibrillation; the mechanism was related to regulation of the IL‐17 signaling pathway and key genes such as Col1a1 and Fasn [492].

#### 5.4.4 Hypertension

Hypertension is a clinical syndrome characterized by elevated systemic arterial pressure and is a major risk factor for cardiovascular diseases [493]. Curcumin exerts antihypertensive effects through multiple mechanisms, including improving vascular endothelial function, suppressing vascular inflammation, and regulating the gut–brain axis.

##### 5.4.4.1 In Vitro Studies

In vitro experiments have shown that curcumin promotes endothelial nitric oxide production, improves vasodilation, and inhibits the abnormal proliferation and migration of vascular smooth muscle cells. Carvedilol and curcumin coloaded in Soluplus micelles achieved sustained and controlled release in vitro while preserving the antioxidant activity of curcumin [494]. Coadministration of curcumin with amlodipine produced a stronger vasorelaxant effect than amlodipine alone in isolated rat aortic rings [495]. Network pharmacology and molecular docking revealed that curcumin targets genes such as PPARG, NOS3, IL6, INS, and ADIPOQ, thereby regulating multiple signaling pathways and miRNAs involved in metabolic syndrome and its components, exerting therapeutic effects [496].

##### 5.4.4.2 In Vivo Studies

Curcumin and its formulations exhibit antihypertensive and target organ protective effects in animal models of hypertension. The novel transdermal curcumin formulation VASCEPTOR improved vascular health in a rat model of hypertension and enhanced exercise tolerance, motor coordination, diastolic function, and reduced frailty and expression of certain age‐related markers in aged mice [497]. Curcumin inhibited PTP1B, DPP‐4, lipase, and α‐amylase activities, activated the insulin signaling pathway, and regulated glycogen metabolic enzymes, significantly reducing blood glucose, HbA1c, oxidative stress markers, and blood lipids in rats with high‐fat, high‐fructose diet‐induced obesity and Type 2 diabetes, while also reducing food intake by 21% [498]. Curcumin reduced blood pressure and scopolamine‐induced cognitive impairment in spontaneously hypertensive rats (SHR/Izm) by inhibiting the expression of ACE and AT1 receptor in brain tissue, decreasing angiotensin II levels, and increasing muscarinic acetylcholine receptor expression and acetylcholine content [499]. Curcumin reshaped the gut microbiota composition, increased plasma butyrate levels, and activated GPCR 43 (GPR43) in the hypothalamic paraventricular nucleus, thereby improving intestinal pathology and integrity, reducing neuroinflammation and oxidative stress, and lowering blood pressure in spontaneously hypertensive rats [500]. Curcumin prevented renal damage in an l‐NAME‐induced hypertensive rat model by reducing MMP‐2 and MMP‐9 activities, decreasing oxidative stress and ED1 expression, improving renal function and glomerular structure, and directly inhibiting MMP‐2 activity in vitro [501].

#### 5.4.5 Cardiomyopathy

Cardiomyopathy refers to a group of diseases involving structural and functional abnormalities of the myocardium, with diabetic cardiomyopathy being a common type [502]. Curcumin exerts protective effects in the treatment of cardiomyopathy by inhibiting programmed cell death pathways such as pyroptosis and ferroptosis. A meta‐analysis of 32 animal studies showed that curcumin dose‐dependently improved cardiac function indices (LVEF, LVFS), cardiac injury markers, heart‐to‐body weight ratio, and blood glucose in animal models of diabetic cardiomyopathy through antioxidant, anti‐inflammatory, autophagy‐enhancing, and antiapoptotic mechanisms, with greater efficacy at doses >200 mg/kg [503].

##### 5.4.5.1 In Vitro Studies

In a high glucose‐induced cardiomyocyte injury model, curcumin treatment alleviated cell damage, suppressed inflammasome activation and the expression of pyroptosis‐related proteins. Curcumin activated SIRT1, upregulated the expression of mitochondrial biogenesis‐related genes including Pgc1α, Tfam, and Nrf2, and inhibited Drp1 translocation to mitochondria, thereby improving mitochondrial morphology and function; it reduced oxidative stress and cardiac dysfunction in LPS‐stimulated HL‐1 cells and in a mouse model of sepsis [504]. The novel curcumin analog SJ‐12 reduced the O‐GlcNAcylation level of SP1 by inhibiting OGT activity, thereby stabilizing SERCA2a; it alleviated cardiac hypertrophy and fibrosis and improved cardiac function in an STZ‐induced diabetic cardiomyopathy mouse model, and the antifibrotic effect was absent in SERCA2a or OGT‐silenced cardiomyocytes [505]. Curcumin promoted Nrf2 nuclear translocation, increased the expression of HO‐1 and Gpx4, reduced lipid peroxide accumulation, inhibited high glucose‐induced ferroptosis in cardiomyocytes, and alleviated myocardial fibrosis and cellular disarray in a diabetic rabbit model [505]. The novel curcumin derivative JM‐2 inhibited NF‐κB activation, alleviated cardiac functional and structural impairment, inflammation and fibrosis in STZ‐induced Type 1 diabetes and HFD‐induced Type 2 diabetes mouse models, and suppressed high glucose‐induced cardiomyocyte hypertrophy and fibrosis in H9c2 cells [506]. Curcumin activated the Nrf2/HO‐1 signaling pathway, reduced ROS generation, increased antioxidant enzyme activity, and inhibited cardiomyocyte apoptosis in high glucose‐treated H9c2 cells and in a high‐fat diet plus STZ‐induced Type 2 diabetic rat model, thereby exerting cardioprotective effects [507].

##### 5.4.5.2 In Vivo Studies

Curcumin and its derivatives exhibit significant cardioprotective effects in animal models of cardiomyopathy. Curcumin pretreatment upregulated apelin expression and downregulated NF‐κB mRNA expression in cardiac tissue, significantly ameliorating doxorubicin‐induced QT interval prolongation, cardiac troponin elevation, oxidative stress, and histopathological damage in rats [473]. Network pharmacology and molecular docking identified AKT1, Bad, and CASP3 as potential targets of curcumin in heatstroke‐induced myocardial injury; in a heatstroke mouse model, curcumin activated the Akt/Bad/Caspase‐3 pathway and alleviated cardiomyocyte edema and mitochondrial swelling [508].Curcumin inhibited NLRP3‐mediated pyroptosis via the AKT/Nrf2/ARE pathway, thereby ameliorating diabetic cardiomyopathy [192]. In a rat model of diabetic cardiomyopathy, both 4 weeks of high‐intensity interval training (HIIT) and curcumin supplementation improved the expression of autophagy‐related genes in cardiomyocytes, with HIIT being more effective than curcumin [509]. Using GEO microarray data and WGCNA analysis, TLR1 was identified as a key target; curcumin downregulated TLR1, inhibited NF‐κB phosphorylation, reduced inflammation and improved cardiac function in a rat model of sepsis. THC inhibited JNK/ERK phosphorylation, reduced proinflammatory cytokine release, activated Nrf2 and other antioxidant proteins, downregulated gp91phox, and modulated the Bcl‐2/Bax/Caspase‐3 pathway, thereby alleviating oxidative stress, inflammation and cardiomyocyte apoptosis in LPS‐induced septic mice and H9c2 cells [510]. Molecular docking identified curcumin as a potent CB2 cannabinoid receptor agonist; in a mouse model of diabetes combined with isoproterenol‐induced myocardial infarction, curcumin improved hemodynamics, lipid profile, antioxidant capacity and inflammatory factors via CB2R activation, restoring cardiac function, while the CB2R antagonist AM630 attenuated these protective effects [511]. THC upregulated MKP‐1, inhibited JNK/ERK phosphorylation, reduced proinflammatory cytokine release, activated Nrf2 and other antioxidant proteins, downregulated gp91phox, and modulated the Bcl‐2/Bax/Caspase‐3 pathway, thereby alleviating oxidative stress, inflammation, and cardiomyocyte apoptosis in LPS‐induced septic mice and H9c2 cells, and improving cardiac function [512].

### 5.5 Inflammatory Conditions

#### 5.5.1 Enteritis

##### 5.5.1.1 In Vitro Studies

In a hydrogen peroxide‐induced oxidative damage model in Caco‐2 cells, pretreatment with the curcumin–glycyrrhizic acid self‐assembled nanoparticle‐based inulin hydrogel (CURG@IN) significantly increased cell survival from 54.2 to 89.7%, while reducing ROS levels by 68.5% [513]. In the RAW264.7 macrophage study, CURG@IN downregulated the expression of the M1 marker iNOS, which is induced by LPS, by 72.3%, while upregulating the expression of the anti‐inflammatory M2 marker Arg‐1 by 3.1‐fold [514]. A recent study utilized single‐cell RNA sequencing to investigate in depth the regulation of PANoptosis (pan‐apoptosis) in intestinal epithelial cells by curcumin. The study found that LPS stimulation significantly upregulated the expression of pan‐apoptosis‐related markers (ASC, NLRP3, NLRP12, and Caspase‐8) in Caco‐2 cells. Meanwhile, curcumin–CS microspheres (CCM) at concentrations of 20, 40, and 80 µM effectively inhibited LPS‐induced cell death in Caco‐2 cells while significantly reducing the levels of inflammatory cytokines such as IL‐18 and IL‐1β [515].

##### 5.5.1.2 In Vivo Studies

In a mouse model of DSS‐induced colitis, the Disease Activity Index (DAI) in the CURG@IN treatment group decreased from 7.8 to 2.1, and the degree of colonic shortening improved by 64.7% compared with the control group [513]. In another study, the combination of curcumin and fecal microbiota transplantation significantly improved colonic histopathological damage in UC‐model mice. Compared with the control group, levels of the proinflammatory cytokines IL‐1β and TNF‐α in the colonic tissue of mice in the combination group decreased by 64.3 and 61.5%, respectively, while levels of the anti‐inflammatory cytokines IL‐4 and IL‐10 increased by 2.1‐fold and 1.8‐fold, respectively. At the same time, the expression levels of tight junction proteins occludin and ZO‐1 were restored by 58.3 and 52.6%, respectively [516]. In a DSS‐induced mouse colitis model, studies have shown that curcumin not only alleviates inflammation but also significantly improves abnormal intestinal angiogenesis by inhibiting the VEGF‐mediated JAK2/STAT3 signaling pathway. Furthermore, it promotes the expansion of Tregs, shifting the immune microenvironment from a Th1/Th17‐driven proinflammatory state to an anti‐inflammatory state [517].

#### 5.5.2 Gastritis

##### 5.5.2.1 In Vitro Studies

Mechanistic studies have revealed that the oxidation products of curcumin can specifically bind to VacA (vacuolating toxin A), a key virulence factor of Helicobacter pylori, thereby directly inhibiting its ability to induce vacuolation in gastric mucosal epithelial cells [518]. A study systematically evaluated the anti‐Helicobacter pylori activity of curcumin extract (CLE). The results showed that CLE completely inhibited the growth of the Helicobacter pylori SS1 strain in a dose‐dependent manner and exhibited significant synergistic effects when used in combination with amoxicillin [519]. In addition, curcumin derivatives have also demonstrated significant potential; the copper and zinc metal complexes of diacetylcurcumin (DAC2‐Cu and DAC2‐Zn) have shown strong anti‐Helicobacter pylori activity, specifically inhibiting the growth of this bacterium and its urease activity [520].

##### 5.5.2.2 In Vivo Studies

Animal model studies have shown that curcumin can inhibit H. pylori virulence and reduce levels of inflammatory cytokines and oxidative stress [518, 521]. In a mouse model of Helicobacter pylori infection, the extent of gastric mucosal inflammation was significantly reduced in the curcumin‐treated group. Compared with the infected group, levels of the proinflammatory cytokines IL‐1β and TNF‐α in the serum of mice in the curcumin group decreased by 46.2 and 52.8%, respectively, levels of the oxidative stress marker MDA decreased by 41.3%, while the activities of SOD and CAT increased by 38.7 and 45.6%, respectively [522]. In a mouse model of H. pylori infection, curcumin not only significantly reduced proinflammatory factors, but more importantly, in long‐term administration studies, it demonstrated strong potential to prevent the malignant progression of chronic atrophic gastritis to gastric cancer (as a chemopreventive agent) by modulating the host's innate and adaptive immune responses, without causing significant histological toxicity [523].

#### 5.5.3 Dermatitis

##### 5.5.3.1 In Vitro Studies

Treatment of human immortalized keratinocytes (HaCaT) with a novel “curcumin–ionic liquid–liposome (Cur–Bet–IL–Lip)” compound was found to significantly downregulate the expression of IL‐17A, IL‐17F, and IL‐22, while promoting the synthesis of Collagen I in the dermis, with a cumulative transdermal penetration rate reaching an impressive 49% [288]. To address the challenge of skin penetration faced by natural compounds such as curcumin in the treatment of psoriasis, researchers developed a nanostructured lipid carrier (NLC) coencapsulating curcumin and sesame oil. This NLC has a particle size of approximately 132.4 nm and an encapsulation efficiency as high as 93%, enabling 24‐h sustained release. Furthermore, MTT assays confirmed its good biocompatibility and the absence of toxic side effects on human fibroblasts [524].

##### 5.5.3.2 In Vivo Studies

In a study of mitochondria‐targeted curcumin microneedles (Cur–TPP@Mil MNs), in a mouse model of IMQ‐induced psoriasis, epidermal thickness decreased significantly from 125 to 38 µm (a 70% reduction) after 10 days of treatment. Key inflammatory factors in the lesions, including IL‐17A, decreased by 60%, while TNF‐α and IL‐6 decreased by 42.1 and 53.8%, respectively [454]. This treatment increased the apoptosis rate of keratinocytes to 35.7%, while the number of cells positive for the proliferation marker Ki67 decreased by 61.3%. In addition, a nano‐composite hydrogel patch composed of natural derivatives possesses dual functions: chemically scavenging ROS and physically absorbing radiation, thereby mitigating radiation damage. At the same time, the patch exhibits strong ROS‐scavenging capacity and promotes M2 polarization of macrophages; and the high‐water contents of the GelMA hydrogel enables it to physically absorb low‐energy X‐rays, thereby providing radiation shielding [525].

#### 5.5.4 Pneumonia

##### 5.5.4.1 In Vitro Studies

In an A549 alveolar epithelial cell model, pretreatment with curcumin (20 µmol/L) effectively reversed LPS‐induced glycocalyx damage, restored heparan sulfate (HS) expression, and inhibited the excessive activation of HS protease (HPSE) [526]. In RAW264.7 macrophages, curcumin (5–20 µmol/L) alleviates LPS‐induced damage by modulating MMP and the TLR4/NF‐κB signaling pathway [527]. Curcumin inhibits the maturation of immature DCs derived from the bone marrow and reduces the expression of their costimulatory molecules (CD80/CD86), thereby cutting off the amplification of inflammation at the antigen‐presentation stage [528].

##### 5.5.4.2 In Vivo Studies

In a mouse model of LPS‐induced acute lung injury (ALI), treatment with curcumin (200 mg/kg) significantly reduced pulmonary edema and inflammatory infiltration. Compared with the control group, mice in the curcumin group exhibited significantly lower total cell counts, neutrophil counts, and protein concentrations in bronchoalveolar lavage fluid (BALF), along with a marked decrease in myeloperoxidase (MPO) activity and MDA levels in lung tissue [526]. In a mouse model of COPD, following inhalation of PEG‐modified curcumin SLNs (PEG–Cur–FL), the concentrations of the proinflammatory cytokines IL‐1β and IL‐6 in BALF decreased by 54.2 and 63.1%, respectively, while the expression of the protective receptor ACE2 was upregulated [529]. Curcumin can also influence the morphology and function of DCs by activating the Wnt/β‐catenin signaling pathway, thereby alleviating asthma symptoms and inflammatory responses [530]. In an allergen‐induced asthma/airway remodeling mouse model, curcumin specifically activates the Wnt/β‐catenin signaling pathway. This not only alters the morphology and function of airway DCs but also significantly alleviates airway hyperresponsiveness (AHR) and eosinophil infiltration in the lungs.

## 6 Clinical Trials

In both in vitro and in vivo preclinical studies, curcumin has demonstrated promising therapeutic effects in neurological disorders, cardiovascular diseases, cancer, inflammatory conditions, and autoimmune diseases. To assess the translational potential of curcumin during the progression of these conditions, recent advancements in its clinical application in this context are summarized, aiming to clarify its therapeutic value in the field.

### 6.1 Neurological Disorders

Hizomi et al. reported three cases of patients with AD who were administered oral turmeric powder capsules over 12 weeks. Following treatment, all patients demonstrated significant improvements in cognitive performance, as measured by the Mini‐Mental State Examination Assessment Scale, along with the alleviation of negative emotions based on the NPI‐Q Subscale. These outcomes contributed to improvements in quality of life and activities of daily living [531]. In a randomized, double‐blind, placebo‐controlled trial, 40 patients with AD, aged 50–84 years, were randomly allocated to either a curcumin capsule (90 mg) treatment group or a placebo group for a treatment duration of 18 months. The results showed that, compared with patients in the placebo group, those in the curcumin treatment group exhibited improvements in short‐term memory and cognitive abilities. FDDNP‐PET scan results indicated that curcumin reduced amyloid and tau protein accumulation in the amygdala and hypothalamus, suggesting that the treatment potentially prevents or delays the progression of neurodegeneration and mitigates symptoms at AD onset [532].

The efficacy of combined treatment with curcumin extract and saffron was evaluated in a 12‐week randomized, double‐blind, placebo‐controlled study. Compared with the placebo, the combination treatment significantly improved depressive symptoms and significantly reduced the STAI and IDS‐SR 30 scores of patients [533]. In another trial, 108 patients with depression were administered 1000 mg of curcumin capsules daily for 6 weeks. Patients receiving curcumin had significantly lower HDRS‐17 and MADRS scores than the controls. Moreover, compared with the placebo treatment, curcumin supplementation significantly lowered the levels of the proinflammatory cytokines IL‐1β and TNF‐α, reduced cortisol secretion, and increased plasma BDNF contents. These findings provided novel insights into the therapeutic potential of curcumin for depression [534]. Panahi et al. conducted a double‐blind, randomized controlled study to evaluate the efficacy of curcumin in patients with major depressive disorder. A total of 111 subjects were randomized into a curcumin–piperine treatment group and a control group, with both groups receiving their respective treatments for 6 weeks. Compared with the control group, the curcumin–piperine group demonstrated significantly lower scores on the HADS and the BDI‐II [535]. Furthermore, in a randomized, double‐blind, placebo‐controlled clinical study, the therapeutic effect of curcumin was investigated in 56 patients with depression. Following 8 weeks of continuous treatment, assessments based on the IDS‐SR 30 and the STAI indicated that curcumin was more effective than the placebo in improving mood‐related symptoms [536].

In a randomized controlled clinical trial, 66 patients with ischemic stroke were randomly assigned to receive either curcumin–piperine tablets or placebo tablets for a total of 12 weeks. At the conclusion of treatment, compared with the placebo, curcumin–piperine supplementation effectively reduced hs‐CRP, TC, and TG, accompanied by a decline in diastolic and systolic blood pressure. Additionally, this treatment enhanced total antioxidant capacity (TAC), contributing to improved health status in patients with ischemic stroke [537].

Donadio et al. investigated the effects of curcumin on the misfolding and accumulation of α‐Syn protein in skin biopsies from patients with PD who were administered a curcumin–phospholipid complex for 12 consecutive months. Patients receiving curcumin treatment exhibited reduced scores on the COMPASS‐31 and NMSS questionnaires, along with decreased α‐Syn accumulation. These findings suggest that the curcumin–phospholipid complex effectively improved clinical parameters by crossing the blood–brain barrier, and also reduced α‐Syn accumulation in the skin of PD patients [538]. In a 9‐month randomized, triple‐blind, placebo‐controlled experiment, 60 patients with PD aged over 30 years of age were randomly assigned to either a curcumin treatment group (*n* = 30) or a placebo group (*n* = 30). After 90 days of curcumin treatment (80 mg/day), scores on the MDS‐UPDRS were significantly decreased relative to those of the placebo controls, suggesting that the quality of life of the patients had significantly improved [539].

Dolati et al. investigated the effects of nanocurcumin on the regulation of abnormal miRNA expression profiles, transcription factors, and proinflammatory factors in peripheral blood mononuclear cells of patients with relapsing‐remitting MS. The results showed that after 6 months of oral nanocurcumin administration, the expression levels of miRNA‐145, miRNA‐132, and miRNA‐16 were reduced, alongside decreases in the secretion levels of IL‐6, IL‐1β, INF‐γ, and CCL‐5. Overall, compared with the placebo, the group supplemented with nanocurcumin showed significant improvements in Expanded Disability Status Scale scores [540]. In another 12‐month double‐blind, randomized, placebo‐controlled trial, 54 ALS patients were allocated in a 1:1 ratio to either a nanocurcumin group (*n* = 27) or a placebo group (*n* = 27), receiving daily doses of 80 mg of nanocurcumin or a placebo. Nanocurcumin was found to be well‐tolerated and improved survival probability, particularly for patients with poor prognoses exhibiting bulbar symptoms, thus establishing it as an effective adjunctive therapeutic agent for ALS patients [541]. In a separate study, 42 ALS patients were treated with 600 mg of oral curcumin and coenzyme Q10 for 6 months, and their Revised ALS Functional Rating Scale (ALS‐FRS‐r) scores remained stable, suggesting that curcumin exerts a protective effect on respiratory function. Additionally, significant reductions in advanced oxidation protein products (AOPPs) and lactate levels were noted, indicating that curcumin may alleviate oxidative stress and improve aerobic metabolism, thereby highlighting its potential therapeutic value for ALS [542]. Julio et al. randomly assigned selected ALS patients to either an intervention group receiving oral liposomal curcumin combined with dutasteride for 2 months or a control group receiving placebo treatment. They reported that, compared with the control group, patients in the intervention group experienced significant muscle bundle stabilization and improvements in muscle capability, which subsequently enhanced their quality of life [543].

### 6.2 Cancer

Clinical investigations into curcumin for colorectal cancer have largely concentrated on its potential as an adjuvant therapy and chemopreventive agent; however, existing data indicate considerable uncertainty regarding its therapeutic effectiveness. In the context of adjuvant chemotherapy, a 2025 randomized controlled trial (RCT) assessed the efficacy of an oral nano‐curcumin formulation in patients with metastatic colorectal cancer. This trial involved 94 patients undergoing XELOX/FOLFOX bevacizumab chemotherapy, who were randomized to receive either nano‐curcumin (40 mg thrice daily) or placebo for six cycles. The study found no statistically significant differences between the nano‐curcumin and placebo groups in carcinoembryonic antigen levels or RECIST‐defined imaging response rates, implying that nano‐curcumin did not substantially enhance treatment outcomes for metastatic colorectal cancer at the administered dosage [544]. However, the clinical application of nanocurcumin in metastatic colorectal cancer remains in the exploratory stage, with limited sample size and insufficient supporting data. Furthermore, variations in curcumin dosage, formulation types, and therapeutic strategies may affect its absorption efficiency, bioavailability, and distribution, resulting in a lack of statistically significant effects. Therefore, future studies should include larger sample size and optimize dosing regimens to further clarify the clinical value of curcumin in colorectal cancer treatment. Concerning the concurrent use of preoperative chemoradiotherapy, a Phase II randomized double‐blind trial (NCT00745134) targeting locally advanced rectal cancer investigated curcumin's efficacy when combined with preoperative chemoradiotherapy. Twenty‐two patients were allocated to receive either curcumin (4 g twice daily) or placebo throughout the chemoradiotherapy course and for 6 weeks postoperatively. The findings indicated that the pathological complete response rate in the curcumin arm was not significantly greater than in the placebo arm (*p* = 0.18). Furthermore, pharmacokinetic analyses demonstrated substantial interindividual variability in curcumin plasma concentrations (ranging from 8.8 to 258.1% of baseline 1 h postadministration) and highly variable tissue concentrations (0.1–4765.7 ng/mg tissue). This unpredictable bioavailability represents a critical obstacle to its therapeutic efficacy [545]. Within the scope of chemoprevention, curcumin has primarily been examined for its role in preventing colorectal adenomas, which are precancerous lesions. Separately, Tolentino et al. developed a CS bioadhesive film loaded with curcumin to assess its safety and efficacy for topical skin cancer treatment. This evaluation encompassed in vitro release kinetics, skin penetration studies, cell viability assays, and irritation testing. The developed film demonstrated robust physical stability at ambient temperatures for 90 days and controlled drug release, with 51.6–65.6% released within 8 h. Curcumin penetration into the skin's stratum corneum and deeper tissues was significantly enhanced, reaching 1.3–1.9 and 1.7–2.7 µg/cm^2^, respectively. In metastatic melanoma MeWo cells, curcumin monotherapy exhibited greater inhibition of cell viability compared with radiotherapy (4–12 Gy) (IC_50_ = 24.4 µg/mL); however, no synergistic effect was observed with their combined administration. Furthermore, the film demonstrated a lack of irritancy in the chicken embryo chorioallantoic membrane assay, and CS exhibited no cytotoxicity against HaCaT keratinocytes. Collectively, this CS film represents a safe and stable topical delivery system for curcumin, showing significant potential for skin cancer therapeutics [546].

### 6.3 Autoimmune Diseases

A randomized, double‐blind, placebo‐controlled trial comprising 48 women diagnosed with RA investigated the effects of a daily 500 mg curcumin supplement administered over 8 weeks. The curcumin cohort demonstrated a statistically significant augmentation in serum TAC, a concomitant reduction in MDA concentrations, and diminished Disease Activity Score‐28 (DAS‐28) scores. Furthermore, notable improvements were observed in joint tenderness, swollen joint count, and pain intensity, collectively suggesting that curcumin can potentiate antioxidant defenses and attenuate disease activity [547]. Conversely, a meta‐analysis incorporating seven RCTs involving 314 patients revealed that curcumin and its derivatives did not yield significant improvements in DAS‐28, ESR, or CRP levels among individuals with RA and SLE, with standardized mean differences of −2.34, −1.49, and −1.65, respectively. Substantial heterogeneity was noted across all outcome measures, indicating that the anti‐inflammatory efficacy of curcumin in these conditions is likely constrained and variable [548]. A randomized, triple‐blind, placebo‐controlled trial enrolling 62 patients diagnosed with SLE demonstrated that a daily oral regimen of 1000 mg curcumin administered over 10 weeks resulted in a statistically significant reduction in anti‐double‐stranded DNA (anti‐dsDNA) and IL‐6 levels. Specifically, change scores for anti‐dsDNA were −9.30 in the curcumin group versus −2.55 in the placebo group (*p* = 0.018), and for IL‐6, they were −25.62 versus −7.34 (*p* = 0.043). These findings suggest that curcumin functions as a safe and efficacious adjunctive therapeutic agent, capable of ameliorating autoimmune activity and inflammatory status [549]. Furthermore, a meta‐analysis incorporating seven RCTs with a cumulative participant count of 314 patients assessed the impact of curcumin and turmeric extract on inflammatory markers in individuals with RA and SLE. While no significant intergroup differences were observed for DAS‐28, erythrocyte sedimentation rate (ESR), and CRP levels, substantial heterogeneity was noted across all outcome measures. This heterogeneity implies that the observed anti‐inflammatory effects were constrained and exhibited inconsistency [548]. A Phase I clinical investigation in five individuals with mild‐to‐moderate psoriasis demonstrated significant amelioration of erythema and scaling in skin lesions following a 4‐week regimen of topical curcumin–vesicle nanocarrier application (CUR 15 µM). Concurrently, a reduction in the expression of IL‐17, IL‐23, IL‐22, TNF‐α, S100A7, S100A12, and Ki67 was observed in lesional tissue. These findings suggest that the antipsoriatic efficacy of this nanoparticle formulation is mediated through the inhibition of the IL‐17/IL‐23 signaling pathway [550]. In an 8‐week randomized, double‐blind, placebo‐controlled trial, 101 patients diagnosed with knee osteoarthritis were administered 500 mg of standardized curcumin extract (Curcugen) twice daily. The findings indicated that, in comparison with the placebo group, curcumin administration led to a significant reduction in KOOS knee pain scores and numerical pain scores. Furthermore, it enhanced performance on the timed up‐and‐go test and the 6‐min walk test, improved JOA total scores, and decreased the need for analgesics in 37% of participants. These outcomes suggest the potential efficacy of curcumin in alleviating osteoarthritis pain and enhancing joint function [551]. A separate 12‐week, three‐arm, randomized, double‐blind, placebo‐controlled trial involved 201 patients with osteoarthritis who received either a curcumin complex (CuraMed) or a curcumin–boswellic acid complex (Curamin) orally, three times daily. The study revealed that both formulations resulted in improvements in joint pain and physical function. Notably, Curamin demonstrated superior performance compared with the placebo in both pain indices and physical performance tests, implying a synergistic interaction between curcumin and boswellic acid [552].

### 6.4 Cardiovascular Diseases

Clinical studies provide evidence for the antiatherosclerotic effects of curcumin. In a RCT involving 72 patients with Type 2 diabetes and an ASCVD risk score ≥5%, curcumin significantly reduced systolic and diastolic blood pressure, LDL‐C, TNF‐α, and MDA levels, increased HDL‐C, improved ASCVD risk classification, and was well tolerated, but had no significant effect on HbA1c, fasting blood glucose, total cholesterol, or triglycerides [553]. In a randomized, double‐blind, placebo‐controlled trial involving 227 obese patients with Type 2 diabetes, 12 months of curcumin intervention significantly reduced pulse wave velocity, LDL‐C, small dense LDL‐C, high‐sensitivity CRP, IL‐1β, IL‐6, and TNF‐α levels, indicating that curcumin effectively reduces atherogenic risk in this population [554]. In a double‐blind, RCT involving 50 patients with stable coronary heart disease, curcumin (45 mg/day) administered from 7 days before to 2 days after percutaneous coronary intervention (PCI) did not show a significant difference compared with placebo, but within‐group analysis showed that curcumin significantly reduced serum hs‐CRP (*p* = 0.006) and sCD40L (*p* = 0.002) levels 48 h after PCI [555]. In a 12‐week randomized, double‐blind, placebo‐controlled trial involving 42 overweight or obese patients with coronary slow flow phenomenon, nano‐curcumin (80 mg/day) significantly improved all domains of the Seattle Angina Questionnaire score and reduced triglycerides, the triglyceride/HDL ratio, visceral adiposity index, and waist‐triglyceride index, but had no significant effect on endocan, adropin, or homocysteine levels [556].

Clinical studies have evaluated the preventive effect of curcumin on postoperative atrial fibrillation. In a randomized, double‐blind, placebo‐controlled trial involving 80 patients undergoing coronary artery bypass graft (CABG) surgery, postoperative oral administration of curcumin–piperine for 5 consecutive days significantly reduced CRP levels, increased TAC, and slightly reduced creatine kinase‐MB levels, but had no significant effect on Troponin I, lactate dehydrogenase, ejection fraction, or atrial fibrillation [557]. In a randomized, double‐blind, placebo‐controlled trial involving 234 patients undergoing CABG surgery, perioperative administration of a nano‐curcumin formulation (240 mg/day) from 3 days before to 4 days after surgery did not reduce the incidence of postoperative atrial fibrillation, nor did it alter hs‐CRP, MDA, or GSH levels [558]. The findings were limited by the short treatment duration and the lack of evaluation of cytokine and transcription factor expression. Moreover, variations in sample size, curcumin dosage, formulation type, and bioavailability may influence the clinical efficacy. Therefore, more comprehensive studies with optimized designs are required in the future to further elucidate the therapeutic potential of curcumin.

Systematic reviews and meta‐analyses provide evidence for the blood pressure‐lowering effect of curcumin. A systematic review and meta‐analysis of 20 RCTs showed that nano‐curcumin supplementation significantly improved waist circumference, fasting blood glucose, HbA1c, QUICKI, total cholesterol, LDL‐C, HDL‐C, diastolic blood pressure, TAC, MDA, TNF‐α, IL‐6, and hs‐CRP in patients with metabolic syndrome [559]. In a 12‐week double‐blind, randomized clinical trial involving 104 infertile women with polycystic ovary syndrome, a DASH diet combined with curcumin (500 mg twice daily) significantly reduced insulin levels and insulin resistance, but had no significant effect on fasting blood glucose or insulin sensitivity [560].

### 6.5 Inflammatory Conditions

Several meta‐analyses have confirmed that curcumin, when used as an adjunctive therapy, can significantly improve clinical remission rates and endoscopic improvement rates in patients with UC. Furthermore, the combination of curcumin and piperine has been shown to improve body composition in patients with IBD [561]. A meta‐analysis published in 2025, which synthesized 13 placebo‐controlled randomized clinical trials, confirmed that curcumin, when used as an adjunctive therapy, significantly improves clinical remission and response rates in patients with UC, with no significant difference in adverse events compared with the placebo group [562]. As a novel local delivery strategy, the enema formulation, when used in combination with mesalazine suppositories in a 2025 randomized, open‐label trial of patients with distal UC, resulted in significantly higher clinical remission rates (60%) and endoscopic healing rates (53.3%) compared with the group treated with mesalazine alone (remission rate: 36.7%; healing rate: 30%) [37]. A 2025 RCT showed that daily oral administration of 1000 mg of curcumin combined with 10 mg of piperine for 12 weeks significantly improved mild‐to‐moderate inflammatory bowel disease and patients’ body composition, while increasing muscle mass [563]. An RCT on functional dyspepsia showed that curcumin is as effective as omeprazole in significantly reducing pain and bloating [564]. In addition, a Phase IIb clinical trial of Meriva (a curcumin–phospholipid complex) is investigating the role of curcumin in preventing gastric cancer in patients with chronic atrophic gastritis. A 2025 RCT involving ICU patients with sepsis (*n* = 66) demonstrated that the combination of curcumin and piperine significantly reduced inflammatory markers such as CRP and ESR within 7 days, and improved laboratory parameters such as bilirubin and hemoglobin [565]. Another study showed that curcumin nanomicelles can significantly reduce serum levels of IL‐17 and IL‐22, as well as SOFA and MODS scores, in patients with sepsis, suggesting their potential for modulating the immune response and improving organ function [566]. A 2025 triple‐blind RCT (*n* = 144) involving patients with mild COVID‐19 found that oral curcumin (500 mg per dose, three times daily) was similarly effective to ginger extract in significantly reducing CRP and ESR levels, confirming their value in regulating inflammation [567].

The accumulated evidence provides a basis for the clinical application of curcumin in the treatment of neurological disorders, cardiovascular diseases, cancer, inflammatory conditions, and autoimmune diseases. Current delivery methods primarily involve oral administration, frequently as an adjunct to standard pharmacological therapies. To address issues regarding bioavailability, some studies have utilized nanoformulations. Curcumin has shown potential in alleviating disease symptoms, delaying disease progression, and improving survival rates, suggesting that it represents a promising candidate for enhancing treatment outcomes in neurological disorders, cardiovascular diseases, cancer, inflammatory conditions, and autoimmune diseases. However, most research to date has focused on the use of curcumin as a therapeutic intervention rather than as a preventive agent. Notably, studies investigating its prophylactic potential remain limited. Nevertheless, in vitro studies involving curcumin pretreatment in cell models have demonstrated that it can delay occurrence of disease by reducing oxidative stress, decreasing proinflammatory factor release, and improving mitochondrial function. This evidence supports the potential use of curcumin as an early preventive intervention strategy for neurological disorders, cardiovascular diseases, cancer, inflammatory conditions, and autoimmune diseases. warranting further in vivo and clinical studies to validate its efficacy as a preventive agent. Tables 3 summarizes the therapeutic effects observed in clinical trials.

**TABLE 3:** Clinical application of curcumin in the treatment of various diseases.

Disorder	Compound	Sample size	Gender/age	Treatment	Outcome	References
Alzheimer's disease	Curcumin powder capsules	3	Male/female 79–85 years old	764 mg/pd for 12 weeks	MMSE↑, NPI‐Q↓, QOL↑, ADL↑	[531]
	Theracurmin	40	Male/female 51–84 years old	90 mg/bid for 18 weeks	Short‐term memory↑, cognitive ability↑, Aβ↓, pTau↓	[532]
Depression	Curcumin extract and saffron	123	Male/female 18–65 years old	500 mg/bid with saffron 15 mg/bid for 12 weeks	STAI↓, IDS‐SR 30↓	[533]
	Curcumin capsules	108	Male 31–59 years old	Two capsules daily for 6 weeks	HDRS‐17↓, MADRS↓, IL‐1β↓, TNF‐α↓, CORT↓ BDNF↑	[534]
	Curcuminoids‐piperine combination	111	Male/female, 18–65 years	1000 mg + 10 mg/pd for 6 months	HADS↑, BDI‐II↑	[535]
	Curcumin capsules	56	Male/female, 18–65 years old	500 mg/bid for 8 weeks	IDS‐SR 30↓, STAI↓	[536]
Ischemic stroke	Curcumin–piperine tablets	66	Male/female, 20–65 years old (mean age, 59.80 ± 4.25 years)	505 mg/pd for 12 weeks	Serum hs‐CRP↓, TC↓, TG↓, TAC↑, frequency and duration of headache attacks↓	[537]
Parkinson's disease	Curcumin phospholipid complex	40	Male/female, 70–74 years old	1 g/bid for 12 months	COMPASS‐31↓, NMSS↓, α‐Syn↓	[538]
Multiple sclerosis	Sina Curcumin	60	Male/female, >30 years old	80 mg/pd for 9 months	MDS‐UPDRS↓, improved life quality	[539]
	Nanocurcumin capsules (curcumin‐containing nano‐micelles)	85	Male/female, 28–51 years old	for 4 months	Therapeutic effect: restores abnormal miRNA expression in RRMS patients, exerts immunomodulatory effects, and has no systemic adverse reactions	[540]
Amyotrophic lateral sclerosis	Nanocurcumin (Sina curcumin) + riluzole	54	Male/female, 51.5 ± 13.1 years old	80 mg/bid for 12 months	Therapeutic effect: significantly improves the survival rate of ALS patients (3.7 vs. 22.2%), shows greater effectiveness in patients with bulbar symptoms, and has no serious adverse reactions	[541]
	Brainoil (containing 600 mg curcumin + 100 mg coenzyme Q10 + 300 mg *Bacopa monnieri* + 250 mg *Withania somnifera* + 250 mg *Centella asiatica* + 1 mg piperine)	58	Male/female, 39–83 years old	600 mg/qd for 6 months	FRAP↑, T‐SH↑, AOPPs↓, lactate↓ Therapeutic effect: stabilizes the ALS‐FRS‐r score, slows disease progression, and improves oxidative damage and aerobic metabolism	[542]
	Liposomal curcumin + resveratrol + dutasteride	61	Male/female, 56.74 ± 10.47 years old	200 mg/qd for 2 months	Therapeutic effect: enhances the basic electrical activity of upper limb muscles, stabilizes fasciculations, and improves muscle function	[543]
Colon cancer	Nano‐curcumin	60	31 male (51.7%), mean age 51.45 ± 12.58 years	40 mg three times daily for 60 days	FEV1 no significant difference; ACT and AQLQ improved but not significantly different from placebo	[544]
	Curcumin C3 complex	22	Male 59%, median age 60 years	Curcumin 4 g orally twice daily during chemoradiation and for 6 weeks post‐CRT	pCR rate unchanged, plasma/tissue curcumin low, downstaging/local failure/survival unaffected	[545]
Rheumatoid arthritis	Curcumin (500 mg/day; 95% turmeric extract)	44	Female; mean age ∼50 years in both groups	Curcumin 500 mg once daily versus placebo for 8 weeks	Tender joint count↓, swollen joint count↓, VAS pain score↓, DAS‐28↓, serum TAC↑ antioxidant capacity and reduced disease activity in women with RA	[547]
	Curcumin/curcuma longa extract	252	Mixed (male: 0–54%); mean age range: 38.9–55.0 years	Curcumin/curcuma longa extract versus placebo or diclofenac sodium (1 study)	Limited and inconsistent anti‐inflammatory effects; high heterogeneity across all outcomes	[548]
Systemic lupus erythematosus	Curcumin 1000 mg/day for 10 weeks	62	Curcumin group: 28 females, 2 males; placebo group: 30 females, 2 males; age 39–41 years	Curcumin 1000 mg/day (two 500 mg capsules) versus placebo for 10 weeks	Anti‐dsDNA↓, IL‐6↓	[549]
	Curcumin/curcuma longa extract	Intervention: 30; control: 32 (total: 62)	Mean age: 39.4 years; male: 6.4%	Curcumin 1000 mg/day for 10 weeks	No significant effect on inflammatory biomarkers (part of pooled DAS‐28, ESR, and CRP analysis); limited to a single trial, precluding disease‐specific subgroup meta‐analysis	[548]
Psoriasis	Curcumin–niosome topical gel	5	Age 18–60 years	CUR–NIO gel versus placebo applied twice daily for 4 weeks on symmetrical lesions	Redness↓, scaling↓, IL‐17↓, IL‐23↓, IL‐22↓, TNF‐α↓, S100A7↓, S100A12↓, Ki67↓	[550]
Osteoarthritis	Curcumin extract	101	50 males, 51 females; age 45–70 years	Curcugen 500 mg twice daily versus placebo for 8 weeks	KOOS pain↓, pain rating↓, TUG↓, 6‐min walk↑, JOA↑; analgesic use↓ 37 versus 13%	[551]
Atherosclerosis/CHD	Curcumin complex or curcumin–boswellic acid combination	201	13 males, 188 females; age 40–77 years	Curamin (350 mg curcuminoids + 150 mg boswellic acid) or CuraMed (333 mg curcuminoids) 500 mg tid versus placebo for 12 weeks	WOMAC total↓, pain index↓, 30 s‐CST↑, 40 min walk↑, TUG↓, SCT↓	[552]
Atherosclerosis/CSFP	Nano‐curcumin	42	Male/female 35–70 years old	80 mg/day, 12 weeks	Improved SAQ domains; TG↓, TG/HDL, VAI, WTI; no effect on endocan, adropin, Hcy	[556]
Atrial fibrillation	Nano‐curcumin	234	Male/female 18–82 years old	240 mg/day (3 d pre‐ to 4 d postsurgery)	No reduction in POAF; no change in hs‐CRP, MDA, GSH	[559]
Hypertension	High‐absorption curcumin	142	Male/female 20–82 years old	90 mg twice daily, 24 weeks	No change in E/E'; BNP↓; more pronounced in patients <65 years	[560]
Enteritis	Curcumin	pooled analysis	Most of them are patients with mild to moderate ulcerative colitis (UC)	The dosage range is wide (0.1–10 g per day), and it lasts for 1–6 months	Clinical remission rate↑, endoscopic improvement rate↑	[561, 562]
	Curcumin	pooled analysis	Distal UC patients	—	Clinical remission rate↑, endoscopic healing rate↑	[37]
	Curcumin + piperine	51	The average age was 47.7 ± 16.2 years old. 62.7% female	1000 mg curcumin + 10 mg piperine per day for a total of 12 weeks	Inflammation↓, body composition improved↑, muscle mass↑	[563]
Gastritis	Curcumin	206	The average age was 49.7 ± 11.9 years old. 73.4% female	250 mg curcumin capsules, four times a day for a total of 28 days	Pain↓, fullness↓	[564]
Sepsis	Curcumin + piperine	66	The average age was 51.66 ± 19.48 years (intervention group).	Take two tablets daily (each containing 500 mg of curcumin and 5 mg of piperine) for a total of 7 days.	CRP↓, ESR↓, bilirubin/hemoglobin parameters improved↑	[565]
Pneumonia	Turmeric	144	The average age was 37.3 ± 15.1 years old.	500 mg per tablet, three times a day, for a total of 5 days	CRP↓, ESR↓	[567]

Abbreviations: ADL, activities of daily living; ASCVD, atherosclerotic cardiovascular disease; BDI‐II, beck depression inventory‐II; bid, twice per day; BNP, brain natriuretic peptide; CHD, coronary heart disease; COMPASS‐31, composite autonomic symptom score 31; CSFP, coronary slow flow phenomenon; DBP, diastolic blood pressure; DBP, diastolic blood pressure; FBG, fasting blood glucose; GSH, glutathione; HADS, Hospital Anxiety and Depression Scale; HbA1c, glycated hemoglobin; Hcy, homocysteine; HDL C, high density lipoprotein cholesterol; HDRS‐17, Hamilton Depression Rating Scale‐17; hs‐CRP, high‐sensitivity C‐reactive protein; IDS‐SR 30, inventory of depressive symptomatology‐self‐report 30; IL, interleukin; LDL C, low‐density lipoprotein cholesterol; MADRS, Montgomery–Asberg Depression Rating Scale; MDA, malondialdehyde; MDS‐UPDRS, Movement Disorder Society‐Unified Parkinson's Disease Rating Scale; MMSE, mini‐mental state examination; NMSS, Nonmotor Symptoms Scale; NPI‐Q, neuropsychiatric inventory—brief questionnaire; PCI, percutaneous coronary intervention; pd, once per day; POAF, postoperative atrial fibrillation; PWV, pulse wave velocity; QOL, quality of life; QUICKI, quantitative insulin sensitivity check index; RCT, randomized controlled trial; SAQ, Seattle Angina Questionnaire; SBP, systolic blood pressure; sCD40L, soluble CD40 ligand; sdLDL C, small dense low density lipoprotein cholesterol; STAI, Spielberger state‐trait anxiety inventory; T2DM, Type 2 diabetes mellitus; TAC, total antioxidant capacity; TC, total cholesterol; TG, triglycerides; TNF‐α, tumor necrosis factor alpha; VAI, visceral adiposity index; WC, waist circumference; WTI, waist triglyceride index.

## 7 Conclusion and Prospects

Neurological disorders, cardiovascular diseases, cancer, inflammatory conditions, and autoimmune diseases, are regarded as complex multifactorial disorders. These impairments adversely affect health and quality of life. Increasing evidence supports that persistent neuroinflammation, oxidative stress, mitochondrial dysfunction, and progressive neuronal degeneration play critical roles in the onset and progression of these diseases. Activation of the TLR4 inflammasome can trigger inflammatory responses and induce the production of proinflammatory cytokines as well as other mediators involved in inflammatory activation [568].

Given the key role of mitochondria in the regulation of cell survival and death, these organelles are often considered potential therapeutic targets in a variety of diseases. In multiple diseases, oxidative stress mediated by mitochondrial dysfunction can impair the ability of cells to produce sufficient ATP to maintain homeostasis, ultimately leading to apoptosis and the accumulation of protein aggregates [569]. Furthermore, neuroplasticity is a fundamental characteristic of the nervous system. Following neuronal injury in the CNS, functional recovery is achieved through neuroplasticity, which facilitates the formation of new memories and learning processes [570]. Understanding these mechanisms provides crucial insights for targeted therapeutic strategies in the management of diseases.

Current clinical pharmacological strategies for neurological disorders, cardiovascular diseases, cancer, inflammatory conditions, and autoimmune diseases, primarily prioritize symptom management rather than addressing the underlying pathological mechanisms [571]. These conventional approaches are often associated with significant adverse effects and have limited efficacy in halting disease progression. Moreover, these therapies fail to modulate the key signaling pathways that are increasingly recognized as drivers of neurological disorders, cardiovascular diseases, cancer, inflammatory conditions, and autoimmune diseases. In contrast, extensive preclinical research has established that curcumin and its derivatives have the potential to serve as neuroprotective agents capable of targeting signaling pathways associated with neurological disorders, cardiovascular diseases, cancer, inflammatory conditions, and autoimmune diseases [572]. Mechanistic studies on neurological disorders, cardiovascular diseases, cancer, inflammatory conditions, and autoimmune diseases indicate that the therapeutic advantages of curcumin stem from its ability to modulate multiple signaling pathways, including the TLR4/MyD88/NF‐κB, JAK/STAT, and PI3K/AKT pathways, as well as Nrf2/ARE‐mediated antioxidant responses. Notably, curcumin can enhance neuroplasticity by modulating neuro‐signaling cascades, thus increasing dendritic spine density in the hippocampus, mediated through pathways such as BDNF/TrkB/CREB, cAMP/PKA, and Wnt/β‐catenin. Additionally, curcumin exhibits low toxicity, with long‐term usage eliciting significantly fewer side effects than traditional medications [7], and is characterized by a favorable safety profile.

Despite the considerable therapeutic potential of curcumin for neurological disorders, cardiovascular diseases, cancer, inflammatory conditions, and autoimmune diseases, significant challenges remain in translating discoveries from basic research to clinical applications, particularly those stemming from its low bioavailability, which greatly limits therapeutic efficacy. Nanotechnology‐based drug delivery systems offer strategies for enhancing curcumin bioavailability. Compared with free curcumin, nanoformulations demonstrate improved stability and biocompatibility, increased blood–brain barrier permeating ability, and significantly enhanced solubility, which together facilitate the specific delivery of curcumin to the brain. Owing to the numerous advantages of nanocarriers, nanoparticles have been widely employed in the development of curcumin nano‐delivery systems. Common carriers include PRO–POL complexes, PLGA, SLNs, and AuNPs, which significantly improve stability and enable controlled drug release. Furthermore, novel nanoformulations such as liposomes, NEs, polymeric micelles, and exosomes have shown marked efficacy in enhancing the pharmacokinetics of curcumin. These delivery strategies circumvent the limitations of the chemical properties of curcumin, potentially avoiding neurotoxicity and enhancing neuronal survival, thereby exhibiting more robust neuroprotective characteristics.

Supported by advancements in scientific technology, substantial progress has been made in curcumin research. Despite the extensive accumulation of in vitro, in vivo, and clinical data, clinical translation remains constrained by the limitations of human studies. For instance, many current clinical trials have inadequate sample sizes and short follow‐up durations, generating uncertainty regarding the long‐term efficacy of curcumin for patients with neurological disorders. During clinical application, potential drug–drug interactions resulting from the combination of curcumin with other medications have attracted increasing attention, particularly among patients receiving pharmacological treatments. For example, warfarin, a commonly used anticoagulant, has a narrow therapeutic window and its efficacy and safety can be influenced by alterations in drug metabolism and pharmacodynamic responses. Studies have suggested that curcumin may affect the pharmacokinetics of warfarin by modulating CYP450‐related metabolic pathways [573]. However, clinical studies have shown that the combined use of curcumin and warfarin does not result in reduced coagulation function, suggesting that the clinical significance of potential interactions between curcumin and conventional medications requires further investigation over longer periods [574]. Furthermore, in a randomized clinical trial, researchers evaluated the safety and efficacy of oral curcumin combined with conventional chemotherapeutic agents as an adjuvant therapy in cancer patients. The results demonstrated that, compared with patients receiving standard chemotherapy alone, those who received curcumin as an adjunctive treatment achieved higher response rates and improved clinical outcomes, suggesting that curcumin may exert synergistic therapeutic effects when combined with chemotherapeutic agents [575]. However, the safety of combining curcumin with different therapeutic agents requires further validation. Therefore, broader and better‐designed clinical trials are needed to determine the optimal dosing regimens and assess the long‐term safety of curcumin‐based therapies.

## Author Contributions

Xiao‐Man Lv, Yi‐Shu Chen, and Dong‐Dong Qin conceived the manuscript. Lin Wang, Qi‐Fei Pan, Wen‐Juan Long, Xin‐Yao Li, Jian‐Qin Mao, Si‐Tong Zhou, Zi‐Hui Wang, Xue‐Lian Zou, Yu‐Qing She, Ming‐Qin Shi, Yang Zhou, Jia‐Rong Du, and Zhong‐Yang Hu reviewed the literature, drafted the manuscript, and drew the figures. Xiao‐Man Lv, Yi‐Shu Chen, and Dong‐Dong Qin supervised and revised the manuscript. Lin Wang, Qi‐Fei Pan, Wen‐Juan Long, Xin‐Yao Li, and Dong‐Dong Qin revised the manuscript according to the editor's and reviewers’ suggestion. All authors read and approved the final manuscript.

## Funding

This study was supported by the National Natural Science Foundation of China (82661067, 82374425, 82260929); Applied Basic Research Programs of Science and Technology Commission Foundation of Yunnan Province (202301AS070053); Key Laboratory of Traditional Chinese Medicine for Prevention and Treatment of Neuropsychiatric Diseases, Yunnan Provincial Department of Education; Scientific Research Projects for High‐level Talents of Yunnan University of Chinese Medicine (2019YZG01); Young Top‐Notch Talent in 10,000 Talent Program of Yunnan Province (YNWR‐QNBJ‐2019‐235).

## Conflicts of Interest

The authors declare no conflicts of interest.

## Ethics Statement

The authors have nothing to report.

## Data Availability

The data that support the findings of this study are available in the paper.
